# RNF112-mediated FOXM1 ubiquitination suppresses the proliferation and invasion of gastric cancer

**DOI:** 10.1172/jci.insight.166698

**Published:** 2023-06-08

**Authors:** Shengwei Zhang, Jing Wang, Weichao Hu, Lijiao He, Qingyun Tang, Jie Li, Mengmeng Jie, Xinzhe Li, Cheng Liu, Qin Ouyang, Shiming Yang, Changjiang Hu

**Affiliations:** 1 Department of Gastroenterology, Xinqiao Hospital, Third Military Medical University, Chongqing, China.; 2 Medical Research Institute, Southwest University, Chongqing, China.; 3 Department of Pharmaceutical Chemistry, College of Pharmacy and Laboratory Medicine, Third Military Medical University, Chongqing, China.; 4 Chongqing Municipality Clinical Research Center for Gastroenterology, Chongqing, China.

**Keywords:** Gastroenterology, Oncology, Cancer gene therapy, Gastric cancer, Oncogenes

## Abstract

Forkhead box M1 (FOXM1) plays a critical role in development physiologically and tumorigenesis pathologically. However, insufficient efforts have been dedicated to exploring the regulation, in particular the degradation of FOXM1. Here, the ON-TARGETplus siRNA library targeting E3 ligases was used to screen potential candidates to repress FOXM1. Of note, mechanism study revealed that RNF112 directly ubiquitinates FOXM1 in gastric cancer, resulting in a decreased FOXM1 transcriptional network and suppressing the proliferation and invasion of gastric cancer. Interestingly, the well-established small-molecule compound RCM-1 significantly enhanced the interaction between RNF112 and FOXM1, which further promoted FOXM1 ubiquitination and subsequently exerted promising anticancer effects in vitro and in vivo. Altogether, we demonstrate that RNF112 suppresses gastric cancer progression by ubiquitinating FOXM1 and highlight the RNF112/FOXM1 axis serves as both prognosis biomarker and therapeutic target in gastric cancer.

## Graphical abstract

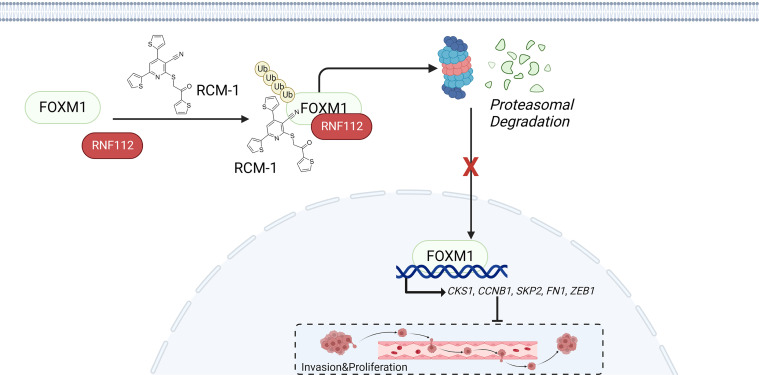

## Introduction

Gastric cancer ranks fifth in terms of its prevalence among all cancers globally and stands as the fourth highest cause of cancer-associated fatalities (1, 2). Although there have been many therapeutic options, including surgical intervention, radiation therapy, chemotherapeutic treatment, and immunological therapy, the therapeutic efficacy of gastric cancer remains unsatisfactory due to the unclear molecular mechanisms involved (3–5). Forkhead box M1 (FOXM1) is a member of the evolutionarily conserved forkhead box family, which is characterized by a winged-helix DNA-binding domain (6). FOXM1 contributes to the occurrence and progression of various types of human malignancies, and it is significantly associated with the poor clinical prognosis of patients with cancer (7–9). Biologically, FOXM1 controls the G1/S and G2/M transition by transcriptionally activating cell cycle–related genes, including *CCNB1*, *CENPA*, and *CENPB* (10). A recent study demonstrated that hyperactivation of FOXM1 can maintain low levels of ROS in gastric cancer, which, in turn, ensures the survival of stem cells present in gastric cancer and induces chemotherapy resistance (11). Moreover, FOXM1 mediates the ferroptosis resistance in melanoma cells in response to anticancer drugs treatment (12). On the other hand, FOXM1 inhibition was found to result in a concomitant reduction in proliferation and glucose metabolism in liver cancer cells (13). Given the significant contribution of FOXM1 in tumor initiation and progression, it is regarded as an attractive target for cancer therapy.

Because there is a large interaction interface but no precisely defined drug-binding regions, transcription factors such as FOXM1 are often considered “undruggable” (14). Previous research primarily focused on disrupting the interaction between FOXM1 and its target genes to suppress the transcriptional activity of FOXM1. For example, siomycin A and thiostrepton have been developed as FOXM1 inhibitors by suppressing the transcriptional activity of FOXM1 (15, 16). In addition, FDI-6 was validated to be a FOXM1 inhibitor by interfering the interaction of FOXM1/DNA, although its efficacy requires a very high concentration (17). It is noteworthy that safety concerns regarding these inhibitors have been raised due to insufficient pharmacological specificity. Furthermore, the lack of cocrystal structure makes it more challenging to explore FOXM1-specific inhibitors.

Therefore, studying the regulatory mechanism of FOXM1 would provide important potential strategies for the pharmacological perturbation of FOXM1. Previous studies suggested that Gli1, STAT3, and LXRα directly bind to the promoter region of *FOXM1* and serve as transcriptional regulators (18–20). In addition, the noncoding RNAs miR-320a, miR-4521, miR-34a, and miR-873-3p have been identified as able to modulate FOXM1 expression posttranscriptionally by directly targeting its 3′-UTR (10, 21–23). The m6A demethylase ALKBH5 controls the expression of FOXM1 by facilitating the demethylation of its pre-mRNA and thereby maintains its stability (24). Moreover, FOXM1 could be modified by phosphorylation, ubiquitination, SUMOylation, and acetylation, which modulate its subcellular localization, protein stability, and transcriptional activity (25). In particular, ubiquitin-mediated protein degradation, due to its high specificity, provides an attractive approach for the precise inhibition, especially for undruggable proteins like FOXM1. Although several E3 ubiquitin ligases targeting FOXM1 have been reported (26–31), further investigation is required to explore the prospect of FOXM1 as an anticancer target in gastric cancer, particularly with respect to its degradation via the ubiquitin-proteasome system.

In this study, we aimed to screen E3 ubiquitin ligases targeting FOXM1 with the ON-TARGETplus siRNA library. RING finger protein 112 (RNF112) was validated as an E3 ubiquitin ligase that directly ubiquitinates FOXM1. Furthermore, RNF112 was proven to serve as a tumor repressor by mediating the proteasomal degradation of FOXM1 and inhibiting its transcriptional network in gastric cancer. Moreover, a small-molecule drug, Robert Costa Memorial drug-1 (RCM-1), a previously identified FOXM1 inhibitor, was verified to dramatically enhance the FOXM1-RNF112 interaction by promoting the cytoplasmic localization of FOXM1. Our results shed light on the mechanism by which RNF112 facilitates the ubiquitination and subsequent degradation of FOXM1. Taken together, these findings suggest that the RNF112/FOXM1 axis could potentially serve as a diagnostic and therapeutic biomarker for gastric cancer.

## Results

### Screen for E3 ligase targeting FOXM1 degradation in gastric cancer.

**Figure 1:**
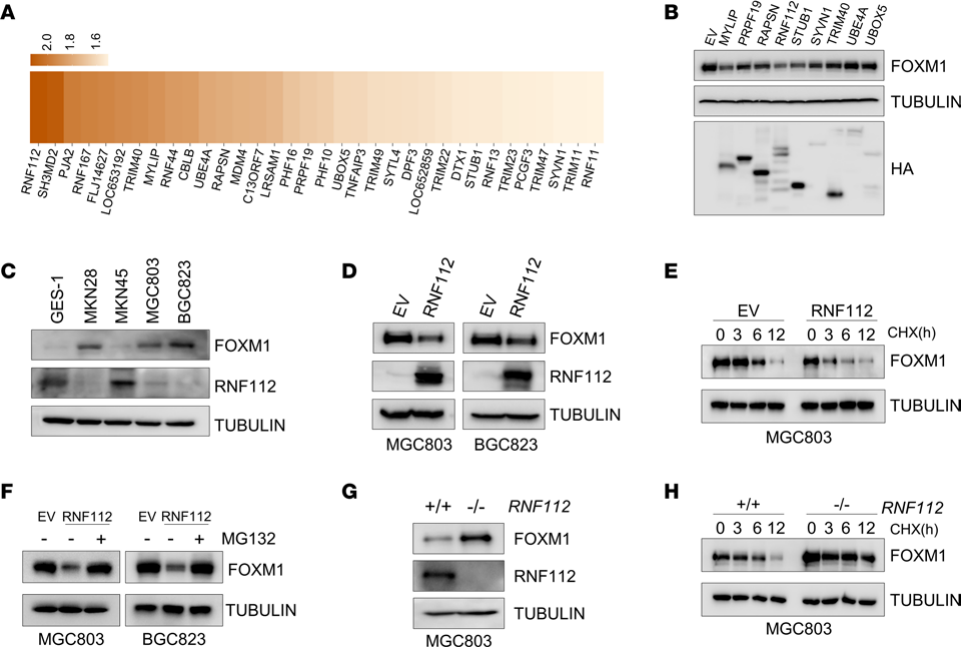
Screen and identification of an E3 ligase targeting FOXM1. (**A**) Heatmap of the fold change in endogenous FOXM1 protein expression after the transfection with siRNA targeting E3 ligases in HEK293T cells. (**B**) Immunoblot detection of FOXM1 in HEK293T cells after transfection with the indicated plasmids. (**C**) Immunoblot analysis of endogenous expression of FOXM1 and RNF112 in GES-1 cells and a panel of human gastric cancer cell lines. (**D**) Immunoblot analysis of endogenous FOXM1 in MGC803 and BGC823 cells after transfection with RNF112-HA plasmids and EV for 48 hours. (**E**) Immunoblot analysis of endogenous FOXM1 after ectopic RNF112 expression in MGC803 cells in the presence of CHX (100 μg/mL) for the indicated time. (**F**) Immunoblot analysis of endogenous FOXM1 expression after ectopic RNF112 expression in MGC803 and BGC823 cells treated with 10 μM MG132 for 8 hours. (**G**) FOXM1 and RNF112 were detected by immunoblotting in WT and *RNF112*-depleted MGC803 cells. (**H**) Immunoblot analysis of endogenous FOXM1 in WT and *RNF112*-depleted MGC803 cells in the presence of CHX (100 μg/mL) for the indicated time. Complete unedited blots are listed in the supplemental material.

The workflow that identifies E3 ubiquitin ligases targeting FOXM1 degradation is shown in Supplemental Figure 1; supplemental material available online with this article; https://doi.org/10.1172/jci.insight.166698DS1 After the transfection of the ON-TARGETplus siRNA library targeting 386 E3 ligases into 293T cells for 48 hours, endogenous FOXM1 expression was assessed. Of note, 34 E3 ligase candidates were identified to decrease FOXM1 abundance more than 1.5-fold (Figure 1A and Supplemental Figure 2; note only those with apparent changes are listed). Subsequently, the E3 ligase prediction tools UbiBrowser (http://ubibrowser.ncpsb.org.cn/ubibrowser/) (32) and UbiNet 2.0 (http://awi.cuhk.edu.cn/~ubinet/ index.php) (33) combined with the gene expression analysis tools Gene Expression Profiling Interactive Analysis (GEPIA; http://gepia.cancer-pku.cn/) and University of Alabama at Birmingham Cancer Data Analysis Portal (UALCAN; http://ualcan.path.uab.edu/) further indicated 77 E3 ligase candidates potentially degrade FOXM1 in gastric cancer. In combination with these findings, only 9 E3 ligases were further studied. Among these ligases, RNF112 was characterized as 1 of the strongest candidates to decrease FOXM1 abundance, via the gain-of-function studies (Figure 1B).

Because FOXM1 could be transcriptionally upregulated by itself (34), we further analyzed the correlation between *RNF112* and *FOXM1* in The Cancer Gene Atlas (TCGA) database. Notably, a marked inverse association between *RNF112* and *FOXM1* was noticed in gastric cancer compared with the other 32 tumor settings, indicating the RNF112/FOXM1 axis is relatively more important in gastric cancer (Supplemental Figure 3A). In the meantime, *RNF112* and *STUB1* expression was significantly suppressed in gastric cancer compared with adjacent paracancer tissues revealed by TCGA data (Supplemental Figure 3B). Interestingly, *RNF112* was most negatively associated with *FOXM1* in gastric cancer tissues (Supplemental Figure 3C).

Next, we evaluated the expression of *RNF112* and the previously reported E3 ubiquitin ligases of FOXM1 in TCGA data, including *APC/C-CDH1*, *VPRBP*, *FBOX31*, *FBXL2*, *FBXW7*, and *RNF168* (26–31). Interestingly, among these E3 ubiquitin ligases, *RNF112* was decreased most dramatically in gastric cancer settings (Supplemental Figure 3D). Furthermore, *RNF112* exhibited the strongest negative association with *FOXM1* in 5 independent gastric cancer cohorts derived from the NCBI Gene Expression Omnibus (GEO) and TCGA (Supplemental Figure 3E). Survival analysis indicates that patients with high *RNF112*/low *FOXM1* have a better outcome than those with low *RNF112*/high *FOXM1*, whereas no noticeable difference was observed on the basis of *FOXM1* or *RNF112* expression alone (Supplemental Figure 3F). The distribution of the patients with gastric cancer based on *FOXM1* and *RNF112* expression was revealed by t-distributed stochastic neighbor embedding analysis (Supplemental Figure 3G).

### RNF112 suppresses FOXM1 protein expression and stability in gastric cancer.

To investigate the potential association between RNF112 and FOXM1 in gastric cancer, FOXM1 and RNF112 expression was detected in GES-1 and gastric cancer cell lines. Interestingly, FOXM1 expression was negatively correlated with that of RNF112 (Figure 1C). Furthermore, ectopic expression of RNF112 dramatically downregulated FOXM1 protein abundance in MGC803 and BGC823 cells (Figure 1D), coupled with a significantly shortened half-life of endogenous FOXM1 protein (Figure 1E). MG132 significantly rescued RNF112-inhibited FOXM1 protein abundance, suggesting that RNF112 suppressed FOXM1 via proteasome-mediated degradation (Figure 1F). On the other hand, FOXM1 expression and protein stability were markedly increased upon *RNF112* depletion via a CRISPR/Cas9–based editing approach in MGC803 cell lines (Figure 1, G and H).

### FOXM1 and RNF112 are both associated with the proliferation and invasion signaling pathways in gastric cancer.

To assess the potential significance of RNF112 in gastric cancer, we examined the downstream mRNA networks of RNF112 and FOXM1 by RNA-Seq. MGC803 cells with *FOXM1* depletion or RNF112 overexpression were analyzed by RNA-Seq (Supplemental Figure 4, A and B). Supplemental Figure 4, C and D reveal the differentially expressed mRNAs in the MGC803 cells with depleted *FOXM1* expression or RNF112 overexpression. Furthermore, Supplemental Figure 4, E–G clearly illustrate that pathways involved in cell proliferation and migration were present in both *FOXM1* deletion and RNF112 overexpression groups (7).

Gene set enrichment analysis was next applied, which revealed that the signaling pathways associated with cell cycle and proliferation (DNA repair, E2F targets, G2M checkpoint) and cell migration and invasion (mTORC1 signaling, Myc targets1, Myc targets2) were dramatically inhibited in patients with high *RNF112* expression, but highly activated in those with high *FOXM1* expression (Supplemental Figure 4H). Collectively, these data suggest that RNF112 may be a potential E3 ubiquitin ligase negatively regulating the FOXM1 transcriptional network.

### RNF112 suppresses the malignancies of gastric cancer cells by suppressing the FOXM1 transcriptional network in vitro.

**Figure 2:**
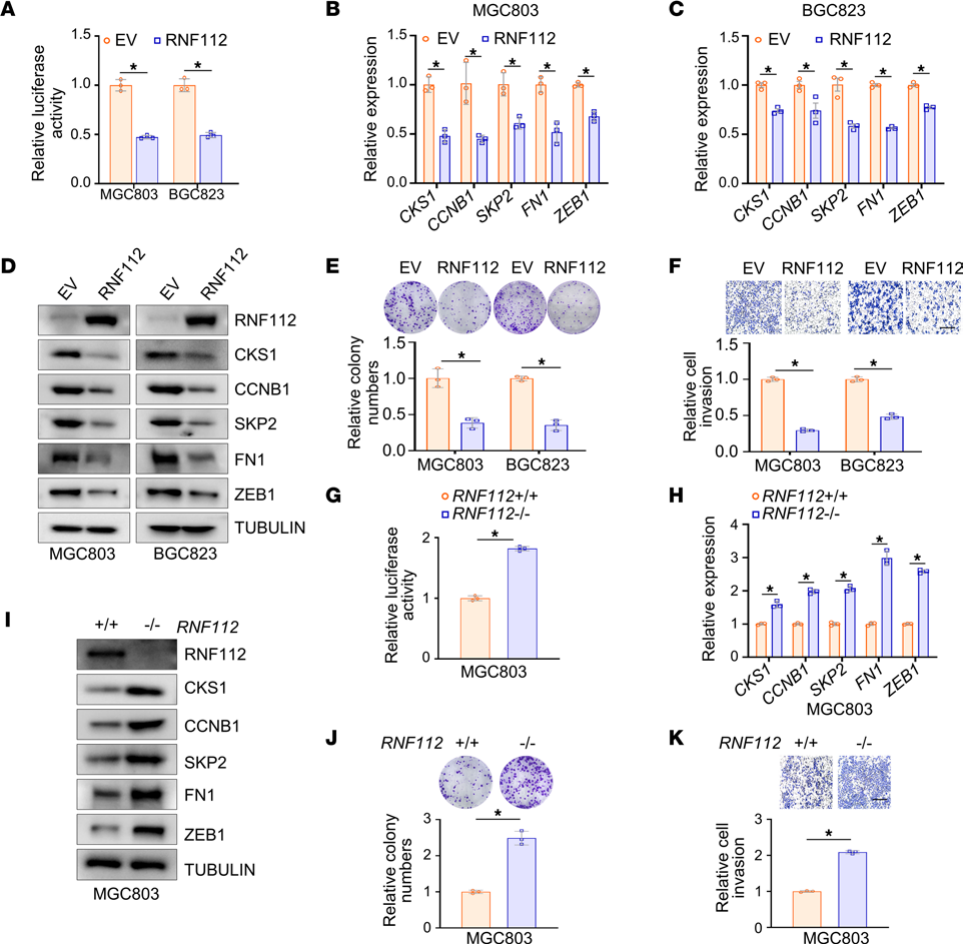
RNF112 suppresses FOXM1 transcriptional targets and inhibits the malignancy of gastric cancer cells in vitro. (**A**) The luciferase activity of 6×FKH luciferase reporter after RNF112 overexpression. MGC803 and BGC823 cells in 24-well plates were transfected with 6×FKH luciferase reporter and pRL-TK, together with RNF112 plasmids or EV, respectively. The luciferase activity was measured 24 hours later (*n* = 3). (**B**–**D**) Quantitative reverse transcription–PCR (qRT–PCR) analysis (**B** and **C**) and immunoblot analysis (**D**) of the expression of FOXM1 target genes after transfection with RNF112-HA plasmids and EV for 48 hours in MGC803 and BGC823 cells (*n* = 3). (**E** and **F**) Representative images of colony-formation assays (**E**) and Transwell invasion assays (**F**) using stably overexpressing EV or RNF112 MGC803 and BGC823 cells. The relative number of colonies and invasive cells was normalized and plotted (*n* = 3). Scale bar: 400 μm. (**G**) The luciferase activity of 6×FKH luciferase reporter in WT and *RNF112*-depleted MGC803 cells (*n* = 3). (**H** and **I**) qRT–PCR analysis (**H**) and immunoblot analysis (**I**) of the expression of FOXM1 target genes in WT and *RNF112*-depleted MGC803 cells (*n* = 3). (**J** and **K**) Representative images of colony-formation assays (**J**) and Transwell invasion assays (**K**) using WT and *RNF112*-depleted MGC803 cells (*n* = 3). Scale bar: 400 μm. Data are presented as mean ± SD. Statistical significance was calculated using Student’s *t* test (**A**–**C**, **E**–**H**, **J**, and **K**). *P < 0.05. Complete unedited blots are in the supplemental material.

Because FOXM1 functions as a critical transcription factor, we asked whether RNF112 suppresses the transcriptional network of FOXM1. In 5 independent gastric and colorectal cancer cohorts obtained from GEO and TCGA, *RNF112* was negatively correlated with *FOXM1* downstream genes, including *CKS1*, *CCNB1*, *SKP2*, and *FN1* (Supplemental Figure 5A). Next, a luciferase reporter containing 6 copies of the FKH binding sequence was constructed (35) (Supplemental Figure 5B). Its activity was significantly downregulated upon ectopic expression of RNF112 (Figure 2A), coupled with reduced expression of FOXM1 downstream genes related to cell proliferation and invasion, including *CKS1*, *CCNB1*, *SKP2*, *FN1*, and *ZEB1* (Figure 2, B–D) (36, 37). Because of the carcinogenic roles of FOXM1 in gastric cancer, ectopic expression of RNF112 markedly inhibited the proliferation and invasion of MGC803 and BGC823 cells (Figure 2, E and F). On the other hand, the activity of the 6×FKH luciferase reporter and expression of downstream genes of FOXM1 were significantly activated upon *RNF112* depletion (Figure 2, G–I). As a result, cell growth and invasion were remarkably enhanced after *RNF112* depletion in MGC803 cells (Figure 2, J and K). To exclude possible off-target effects, we reintroduced exogenous RNF112 in *RNF112*-deficient cells (Supplemental Figure 5, C and D). The results showed that the tumor-promoting effect of *RNF112* depletion was dramatically rescued via ectopic RNF112 expression in *RNF112* KO cells (Supplemental Figure 5, E and F).

To test whether RNF112 inhibits malignant behaviors of gastric cancer by targeting FOXM1, MGC803 and BGC823 cells with RNF112 overexpression were further infected with FOXM1-expressing lentivirus. Supplemental Figure 6, A–C shows that ectopic FOXM1 dramatically restored the RNF112-suppressed FOXM1 downstream genes. More interestingly, FOXM1 restoration also partially rescued RNF112-inhibited proliferation and invasion of gastric cancer (Supplemental Figure 6, D and E). Consistent with this finding, an obvious reversal of FOXM1 downstream genes, cell proliferation, and invasion were noticed in RNF112-depleted MGC803 cells after FOXM1 knockdown (Supplemental Figure 6, F–I). Overall, the above results suggested that the anticancer effect of RNF112 was at least partially owed to FOXM1 degradation.

### RNF112-inhibited FOXM1 expression suppresses gastric cancer malignant behaviors in vivo.

**Figure 3:**
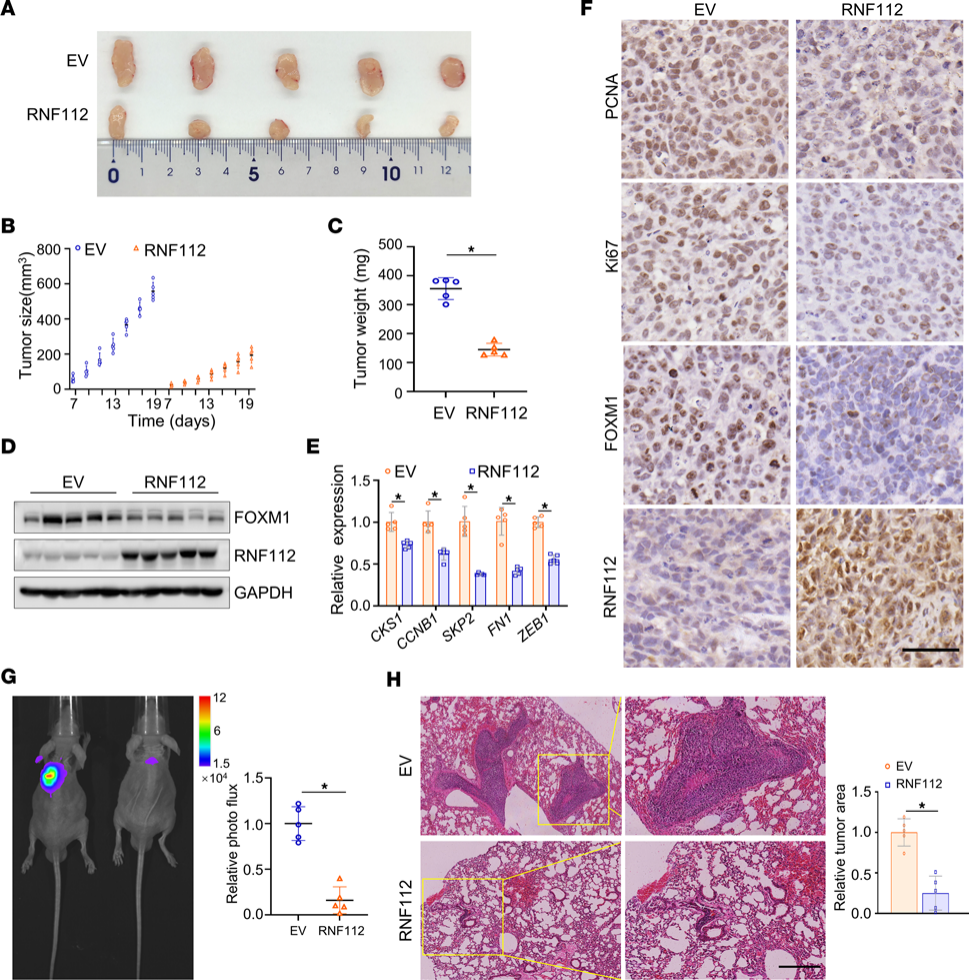
RNF112 suppresses gastric cancer malignant behaviors in vivo. (**A**) Tumors were harvested after s.c. injection of MGC803 cells with stable overexpression of RNF112 in nude mice. On day 19, tumors were removed from the sacrificed mice and used for subsequent analysis (*n* = 5). (**B**) Tumor growth curves of xenograft of the EV and RNF112 overexpression groups (*n* = 5). (**C**) Tumor weight was measured after the mice were sacrificed (*n* = 5). (**D**) Immunoblot analysis of FOXM1 and RNF112 expression of the tumors. (**E**) Quantitative reverse transcription–PCR analysis of FOXM1 downstream genes in the above tumors (*n* = 5). (**F**) IHC staining of PCNA, Ki67, FOXM1, and RNF112 in the indicated groups. (**G**) Bioluminescence imaging of representative mice and statistical analysis of the luminescence intensity (*n* = 5). (**H**) H&E staining of the representative metastatic lesions in the lung of nude mice and the statistical analysis of the relative tumor area (*n* = 5). Scale bar: 50 μm. Data are presented as mean ± SD. Statistical significance was calculated using Student’s *t* test (**C**, **E**, **G**, and **H**). *P < 0.05. Complete unedited blots are in the supplemental material.

The role of RNF112 in vivo was then studied using xenograft tumor models. We observed that RNF112 noticeably decreased tumor growth and weight in vivo (Figure 3, A–C), coupled with the decreased FOXM1 and its downstream genes’ expression (Figure 3, D and E). Consistent with this, FOXM1, Ki67, and PCNA levels were markedly decreased upon RNF112 stable expression in vivo (Figure 3F). Likewise, *RNF112* depletion readily increased tumor growth and weight, coupled with elevated expression of FOXM1 and its downstream target genes, as well as that of Ki67 and PCNA in vivo (Supplemental Figure 7, A–F). These findings together demonstrate that RNF112 plays tumor suppressor roles via disturbing FOXM1 in gastric cancer.

A tail vein–lung metastasis nude mice model was subsequently established to assess the metastatic ability of cancer cells. Bioluminescence imaging and H&E staining clearly demonstrated that RNF112 dramatically repressed lung metastatic lesions in vivo (Figure 3, G and H).

### RNF112 physically interacts with and ubiquitinates FOXM1.

**Figure 4:**
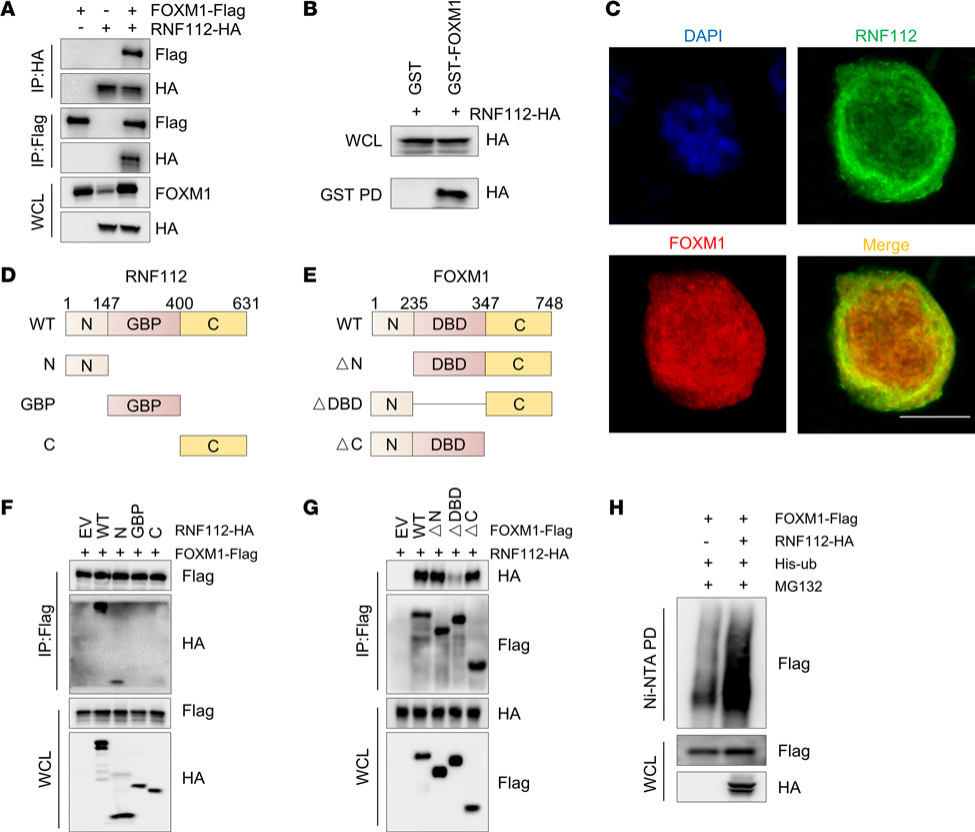
RNF112 physically interacts with and ubiquitinates FOXM1. (**A**) Co-IP analysis of the interaction between RNF112 and FOXM1. HEK293T cells were harvested 48 hours after transfection with FOXM1-FLAG and RNF112-HA, and the cell lysates were used for IP with anti-HA or anti-FLAG agarose beads. Then, HA, FLAG, and FOXM1 were detected by immunoblotting in whole-cell lysates and corresponding precipitates. (**B**) Immunoblot analysis of whole-cell lysates (WCL) and GST pull-down (PD) products derived from HEK293T cells transfected with RNF112-HA plasmid. (**C**) Immunofluorescence of RNF112 and FOXM1 in HEK293T cells transfected with RNF112 and FOXM1 expression vectors. Scale bar: 10 μm. (**D** and **E**) Schematic graph of truncated RNF112 (**D**) and FOXM1 protein (**E**). GBP, guanylate-binding protein family domain; DBD, DNA-binding domain. (**F**) Co-IP analysis of the interaction between truncated RNF112 and full-length FOXM1 in HEK293T cells. (**G**) Co-IP analysis of the interaction between truncated FOXM1 mutants and full-length RNF112 in HEK293T cells. (**H**) Ubiquitination of FOXM1 was tested 48 hours after transfection with RNF112-HA or control in HEK293T cells in the presence of 10 μM MG132 for 8 hours. Complete unedited blots are in the supplemental material.

To assess whether the inhibitory effects of RNF112 on FOXM1 were mediated by the ubiquitination-dependent pathway, Co-IP and GST-pull-down assays were used to detect FOXM1 and RNF112 interaction (Figure 4, A and B). Immunofluorescence analysis showed that RNF112 and FOXM1 mainly colocalized in the cytoplasm, with minor colocalization observed in the nucleus (Figure 4C). To map the binding domains between RNF112 and FOXM1, Co-IP was performed with truncated mutants. As a result, the interaction of the N-terminus of RNF112 (1–147 aa) with the DNA binding domain of FOXM1 (235–347 aa) was validated (Figure 4, D–G). Moreover, a readily increased FOXM1 ubiquitination level was detected after RNF112 overexpression (Figure 4H).

**Figure 5:**
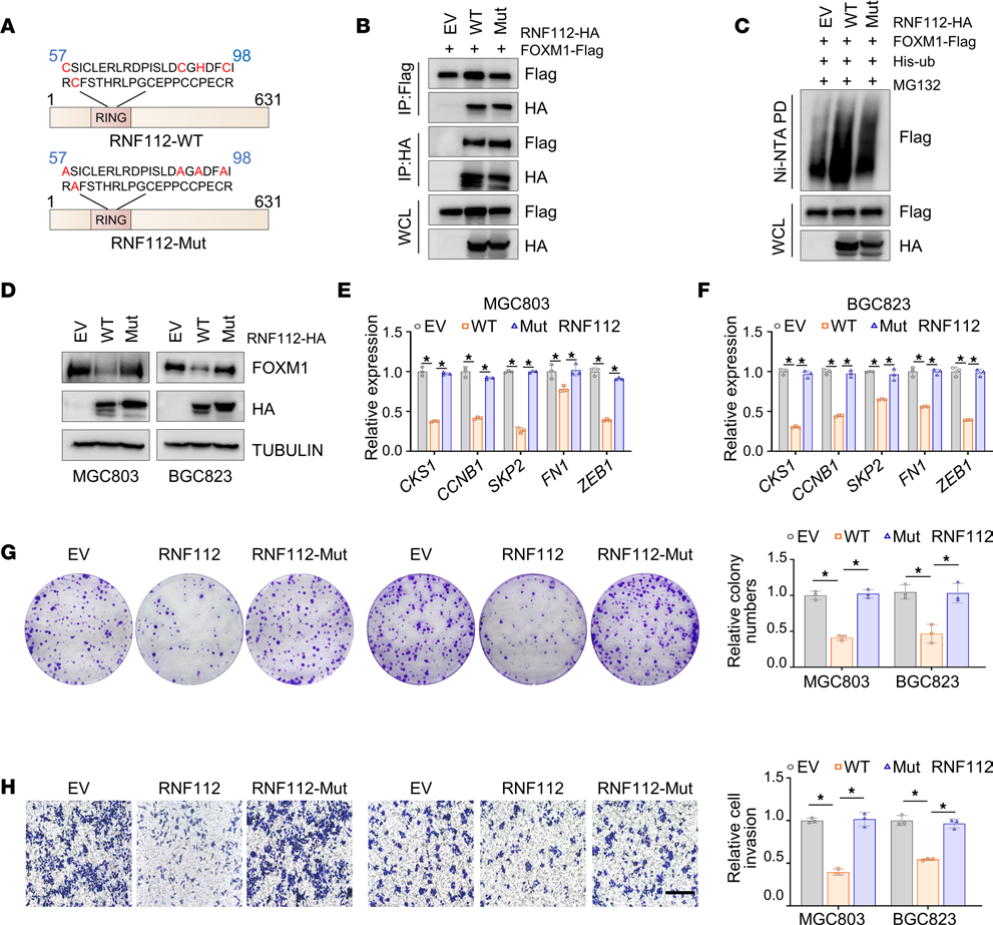
Ubiquitin ligase activity of RNF112 is responsible for FOXM1 ubiquitination and degradation. (**A**) Schematic graph of the RNF112-Mut lacking E3 ubiquitin ligase activity. (**B**) Co-IP assays between RNF112-WT (or RNF112-Mut) and FOXM1 in HEK293T cells. (**C**) Ubiquitination of FOXM1 was tested 48 hours after transfection with RNF112, RNF112-Mut, or EV in HEK293T cells in the presence of 10 μM MG132 for 8 hours. (**D**) Immunoblot analysis of FOXM1 expression in MGC803 and BGC823 cells with EV, RNF112-WT, and RNF112-Mut overexpression. (**E** and **F**) Quantitative reverse transcription–PCR analysis of FOXM1 downstream genes in MGC803 (**E**) and BGC823 (**F**) cells with EV, RNF112-WT, and RNF112-Mut overexpression (*n* = 3). (**G** and **H**) Colony-formation assays (**G**) and Transwell invasion assays (**H**) of the indicated stable overexpression cells (*n* = 3). Scale bar: 400 μm. Data are presented as mean ± SD. Statistical significance was calculated using 1-way ANOVA (**E**–**H**). *P < 0.05. Complete unedited blots are in the supplemental material.

Given that RNF112 belongs to the RING-type E3 ubiquitin ligase, a catalytic dead RNF112 was generated (RNF112-Mut) by mutating the RING domain (Figure 5A) (38). Although the binding affinity between RNF112 and FOXM1 was not affected (Figure 5B), compared with the intact form, RNF112-Mut failed to ubiquitinate and degrade FOXM1 (Figure 5, C and D). As a result, RNF112-Mut was unable to interfere with FOXM1 downstream genes (Figure 5, E and F) and almost lost its tumor suppressor roles in gastric cancer (Figure 5, G and H). Collectively, these findings suggest that the ubiquitin ligase activity of RNF112 is responsible for the FOXM1 ubiquitination and degradation.

### RCM-1 enhances the interaction between RNF112 and FOXM1.

Because RCM-1 was reported to be a novel FOXM1 inhibitor that promotes proteasome-mediated degradation (39), we then asked whether RCM-1 pursued its function via regulating the RNF112-FOXM1 interaction. To this end, network pharmacology–based analysis was first used to investigate the potential targets of RCM-1 in gastric cancer. The predictive targets of RCM-1 were obtained from the SuperPred (40), SwissTargetPrediction (41), and Similarity Ensemble Approach (SEA) databases (42). Then, key genes in gastric cancer were obtained from the GeneCards database (https://www.genecards.org/) (43). After we obtained the shared genes, they were subjected to component-target-disease model construction and gene enrichment analysis (Supplemental Figure 8, A and B). Gene Ontology and Kyoto Encyclopedia of Genes and Genomes analysis indicated that RCM-1 was associated with the protein stability and ubiquitin-related signaling pathways in gastric cancer (Supplemental Figure 8, C–F). These results suggest that RCM-1 may inhibit gastric cancer progression via modulating the protein ubiquitination levels.

**Figure 6:**
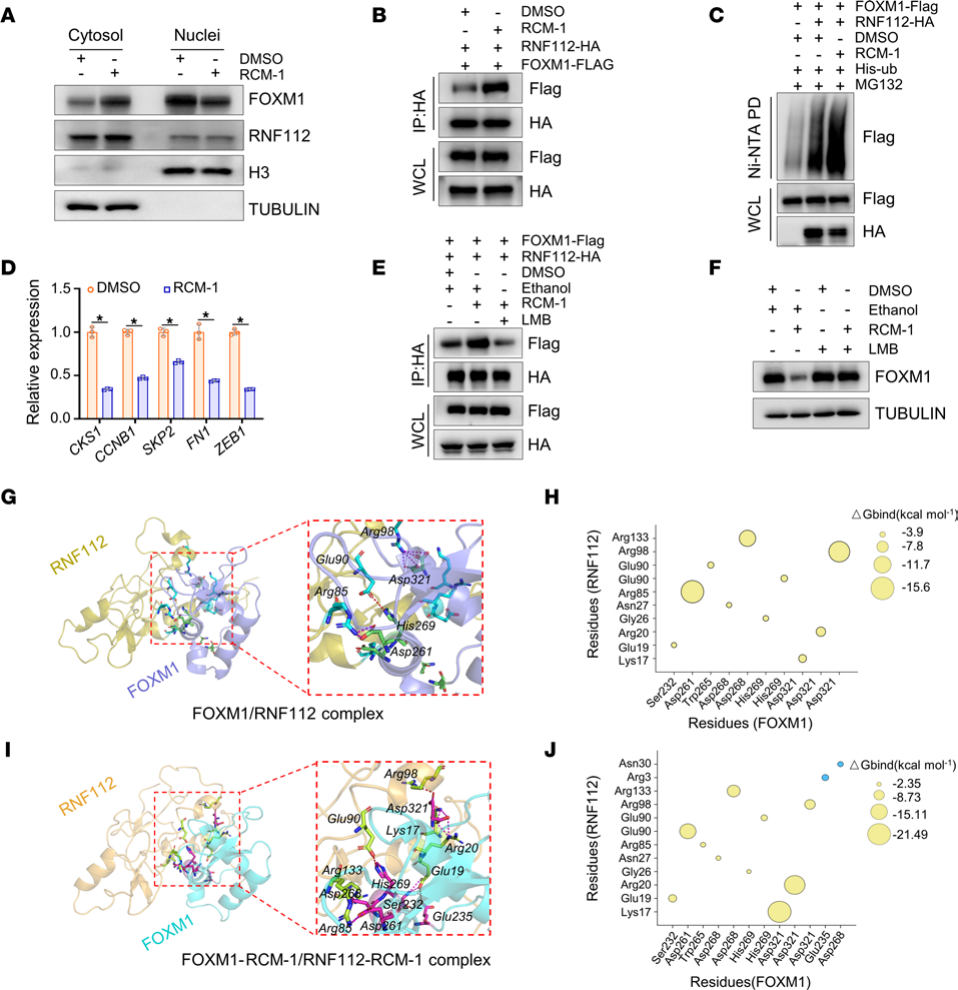
RCM-1 enhances the interaction between RNF112 and FOXM1. (**A**) Immunoblot analysis of the cellular distribution of FOXM1 and RNF112 in MGC803 cells after DMSO or RCM-1 treatment (10 μM). H3 and tubulin were used as markers of nuclear and cytoplasmic fractions, respectively. (**B**) Co-IP analysis of the interaction between FOXM1-FLAG and RNF112-HA in HEK293T cells in the presence of DMSO or RCM-1 (10 μM). (**C**) Ubiquitination of FOXM1 was analyzed after transfection with RNF112 or control in HEK293T cells treated with DMSO or RCM-1 (10 μM). (**D**) Quantitative reverse transcription–PCR analysis of FOXM1 target genes in MGC803 cells treated with DMSO or RCM-1 (10 μM) (*n* = 3). (**E**) Co-IP analysis of RNF112-FOXM1 interaction in HEK293T cells treated with RCM-1 (10 μM) and LMB (25 nM) for 24 hours. (**F**) Immunoblot analysis of endogenous FOXM1 expression in MGC803 cells treated with RCM-1 (10 μM) and LMB (25 nM) for 24 hours. Ethanol and DMSO were used as vehicle controls. (**G**) Docking model of the FOXM1/RNF112 complex. Red and purple dashed lines indicate hydrogen bonds and salt bridges, respectively. (**H**) Bubble plot of binding free energies (ΔGbind) between the key residues of FOXM1 and RNF112. The bubble size corresponds to the absolute value of the binding free energy. (**I**) Docking model of FOXM1/RCM-1–RNF112/RCM-1 complex (RCM-1 is not shown). (**J**) Bubble plot of binding free energies between the key residues of FOXM1 and RNF112 in the presence of RCM-1. Blue bubbles indicate the additional key residues. Statistical significance was calculated using Student’s *t* test (**D**). *P < 0.05. Complete unedited blots are in the supplemental material. WCL, whole-cell lysate.

As shown in Figure 6A and Supplemental Figure 9A, RCM-1 dramatically enhanced the cytoplasmic distribution of intracellular FOXM1 and its colocalization with RNF112 in the cytoplasm. Figure 6B shows that RCM-1 remarkably promoted the interaction between RNF112 and FOXM1. In keeping with this finding, Figure 6, C and D illustrate that FOXM1 underwent apparent ubiquitin modifications upon RCM-1 treatment, accompanied by repressed FOXM1 downstream target genes. Next, the nuclear export inhibitor leptomycin B (LMB) was used to block FOXM1 cytoplasmic translocation. Figure 6E demonstrates that LMB noticeably alleviated the RCM-1–enhanced RNF112-FOXM1 protein interaction. Consistently, the inhibitory effect of RCM-1 on FOXM1 was markedly reversed in response to LMB treatment (Figure 6F). These data demonstrate that RCM-1 enhanced the RNF112-FOXM1 interaction via inducing the cytoplasmic localization of FOXM1.

### Potential binding modes of FOXM1, RNF112, and RCM-1.

To further explore the potential binding modes of FOXM1, RNF112, and RCM-1, we modeled the 3-dimensional structure of RNF112 (1–147 aa) by homological modeling using Modeller 10.3, and the Ramachandran plot indicated its rationality (Supplemental Figure 9B). The structure of the FOXM1 DNA-binding domain (DBD) was accessed through Protein Data Bank (44). The FOXM1/RNF112 complex was then constructed by ZDOCK 3.0.1. The root mean square deviation (RMSD) plot showed the conformation of FOXM1/RNF112 stabilized at 7 Å after 80 ns, and the average structure was extracted for the last 20 ns (Supplemental Figure 9C). We docked the RCM-1 into the stabilized FOXM1/RNF112 complex using Surflex-dock module in SYBYL-2.0. For a 100-ns molecular dynamics (MD) simulation, FOXM1/RNF112, RCM-1/FOXM1, and RCM-1/RNF112 reached stable states, and the RMSD fluctuated around ~5.0 Å, ~3.0 Å, and ~1.5 Å after 50 ns, respectively (Supplemental Figure 9D). The binding free energies for the FOXM1/RNF112 and FOXM1-RCM-1/RNF112-RCM-1 complexes were estimated by molecular mechanics/Poisson Boltzmann surface area (MM/PBSA; –24.01 vs. –38.02 kcal/mol), which indicated RCM-1 could markedly promote potential binding affinity between FOXM1 and RNF112 (Supplemental Figure 9E).

To gain an insight into the binding modes and identify key residues, we performed energy decomposition for 2 complexes by the molecular mechanics/generalized Born surface area (MM/GBSA) method. For the FOXM1/RNF112 complex, 3 salt bridges (Asp321–Arg98 [~2.9 Å and ~2.7 Å], Asp261–Arg85 [~2.8 Å]) and 1 hydrogen bond (His269–Glu90 [~3.4 Å]) were formed among key resides located in the interface between FOXM1 and RNF112 (Figure 6G). Figure 6H shows the binding free energies between crucial residues. When RCM-1 was added to the FOXM1/RNF112 complex, 3 salt bridges (Asp321–Lys17 [~1.7 Å], Asp321–Arg20 [~2.8 Å], and Asp261–Arg85 [~3.2 Å]) and 7 hydrogen bonds (Asp321–Lys17 [~2.8 Å], Asp321–Arg98 [~2.8 Å], His269–Glu90 [~3.0 Å], Asp268–Arg133 [~3.8 Å], Glu235–Glu19 [~3.9 Å], Ser232–Glu19 [~2.9 Å and ~3.0 Å]) were found between different key residues of FOXM1 and RNF112, which could promote their potential affinity (Figure 6I). Consistently, the binding free energies between key residues were significantly decreased. Furthermore, 2 pairs of additional key residues (blue bubbles in Figure 6J) were observed when RCM-1 was added, which indicated that the binding modes between FOXM1 and RNF112 were precisely changed and explained the altered binding free energies (Figure 6J).

Interestingly, RCM-1 presence enhanced the thermal stability of both FOXM1 and RNF112 as validated by the cellular thermal shift assay (Supplemental Figure 10, A and B), suggesting that RCM-1 binds to FOXM1 and RNF112 proteins. Our models illustrated that RCM-1 could bind to FOXM1 through hydrogen bonds with Tyr317 (~3.7 Å) and Lys255 (~3.8 Å), and it could form 1 hydrogen bond with Glu96 of RNF112, which was favorable for FOXM1-RNF112 interaction. Additionally, RCM-1 binds to key residues of RNF112 through hydrogen bonds (Leu110 [~3.6 Å], Pro124 [~4.0 Å], and Ser28 [~4.1 Å]), and hydrophobic interaction (Phe24 [~3.5 Å], Leu110 [~3.7 Å]) (Supplemental Figure 10C). The interaction energy between RCM-1 and key residues and its decomposition are shown in Supplemental Figure 10, D and E, and might guide one to screen potential ligands that promote binding between FOXM1 and RNF112.

### RCM-1 inhibits the expression of FOXM1 partially via an RNF112-dependent mechanism.

**Figure 7:**
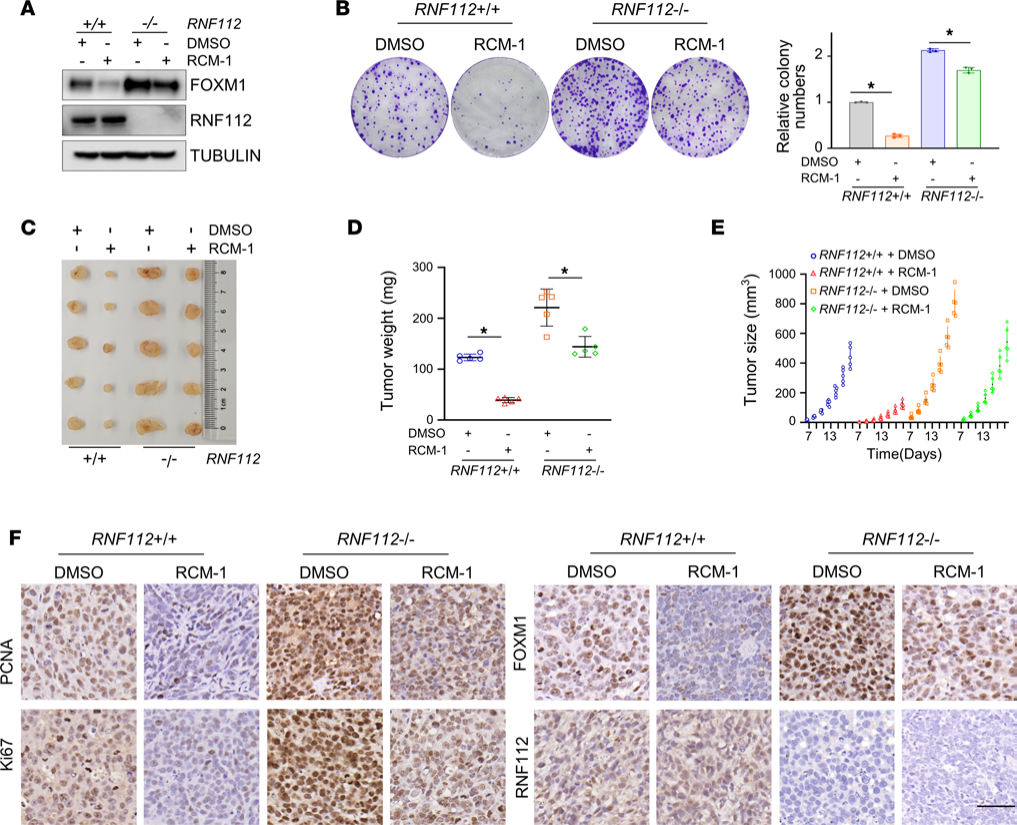
RCM-1 inhibits gastric cancer proliferation partially dependent on RNF112. (**A**) Immunoblot analysis of FOXM1 in *RNF112*^+/+^ and *RNF112*^–/–^ MGC803 cells after treatment with DMSO or 10 μM RCM-1. (**B**) Quantitative reverse transcription–PCR analysis of FOXM1 target genes in MGC803 cells treated with DMSO or RCM-1 (10 μM) (*n* = 3). (**C**) Tumors harvested from the RCM-1–treated xenograft mouse model. The mice were administered DMSO or RCM-1 i.p. from day 7 to the sacrifice day (*n* = 5). (**D** and **E**) Tumor weight (**D**) and growth curves (**E**) of the above RCM-1–treated xenograft mouse model. (**F**) IHC staining of PCNA, Ki67, FOXM1, and RNF112 from the xenograft tumors (*n* = 5). Scale bar: 50 μm. Data are presented as mean ± SD. (**B** and **D**) Statistical significance was calculated using Student’s *t* test. *P < 0.05. WCL, whole-cell lysate.

To further investigate whether the inhibitory effect of RCM-1 on FOXM1 is RNF112 dependent, we treated both WT and *RNF112*-KO MGC803 cells with RCM-1. Our results showed that RCM-1 exhibited a significantly weaker inhibition of FOXM1 expression and cell proliferation upon RNF112 depletion (Figure 7, A and B), indicating RCM-1–mediated FOXM1 suppression is at least partially dependent on RNF112 presence. To echo this finding, we observed that administration of RCM-1 largely attenuated intact but not *RNF112*-deleted gastric cancer growth in a xenograft mouse model (Figure 7, C–F). Altogether, the above results suggest that RCM-1 may promote the interaction between RNF112 and FOXM1 to perform antitumor functions in gastric cancer.

### FOXM1 exhibits inverse correlation with RNF112 in gastric cancer tissues.

**Figure 8:**
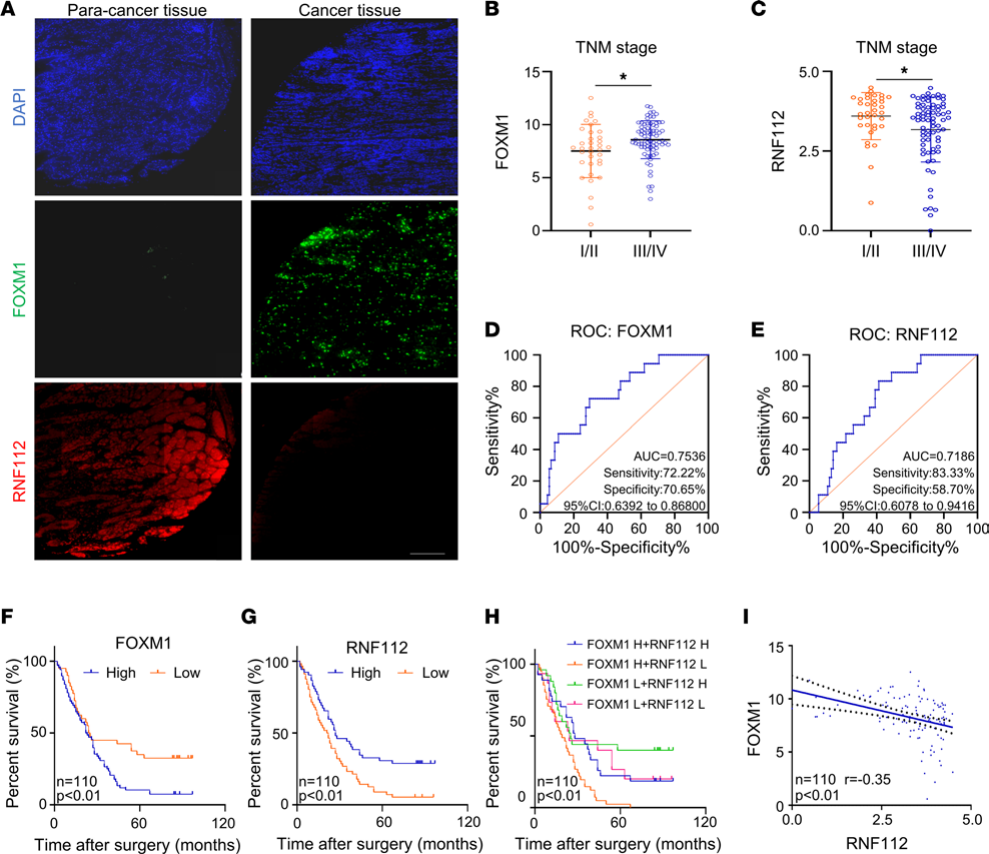
FOXM1 exhibits inverse correlation with RNF112 in gastric cancer tissues. (**A**) Representative multicolor immunofluorescence pictures of FOXM1 and RNF112 in gastric cancer tissues and the corresponding normal tissues. Scale bar: 200 μm. (**B** and **C**) The association between the expression of FOXM1 (**B**) or RNF112 (**C**) and TNM stages in patients with gastric cancer (*n* = 110). (**D** and **E**) The ROC curves for determining the cutoff values of survival analysis of FOXM1 (**D**) and RNF112 (**E**). (**F**–**H**) Kaplan-Meier survival analysis according to the expression of FOXM1 (**F**) or RNF112 (**G**) alone, or the combination of the above 2 indices (**H**) (*n* = 110). (**I**) Correlation of the expression levels between FOXM1 and RNF112 in the gastric cancer tissues (*n* = 110). Data are presented as mean ± SD. Statistical significance was calculated using Mann-Whitney *U* test (**B** and **C**), log-rank test (**F**–**H**), or Pearson’s test (**I**). *P < 0.05.

Then, we explored the clinical relevance of FOXM1 and RNF112 in gastric tissue microarrays (TMAs) (the clinicopathologic features of patients are provided in Supplemental Table 3). Multicolor immunofluorescence analysis revealed that FOXM1 was expressed more highly in cancer tissues and was accompanied by lower RNF112 expression (Figure 8A). Notably, in cancer tissues, prominent colocalization between FOXM1 and RNF112 was observed (Supplemental Figure 11A). Furthermore, FOXM1 was highly expressed in patients with advanced TNM stage, whereas RNF112 levels were lower in those patients (Figure 8, B and C). The receiver operating characteristic (ROC) curves in Figure 8, D and E show that FOXM1 and RNF112 could be good candidates for predicting patients’ survival. Interestingly, patients with gastric cancer with high FOXM1/low RNF112 displayed the shortest survival, indicating that the combination of the 2 indices could predict the survival of patients more sensitively (Figure 8, F–H). Figure 8I identifies the inverse association between RNF112 and FOXM1 in gastric cancer tissues. We next investigated the protein expression of FOXM1, RNF112, CKS1, and ZEB1 in 12 pairs of gastric cancer tissues and corresponding noncancerous, adjacent tissues (Supplemental Figure 11B). The results revealed that RNF112 protein expression was negatively correlated with that of FOXM1, CKS1, and ZEB1 (Supplemental Figure 11C), suggesting the presence of the RNF112/FOXM1 pathway in vivo.

## Discussion

Because of the significance of FOXM1 in cancers, upstream regulation has drawn more attention in recent years, including the exploration of its E3 ligases. Although several E3 ligases have been identified, here, via ON-TARGETplus siRNA library–based approaches, an E3 ubiquitin ligase, RNF112, was identified as promoting the ubiquitination and degradation of FOXM1 in gastric cancer. Further studies demonstrate that RNF112 plays a tumor-suppressor role by targeting FOXM1, whose expression is negatively associated with that of FOXM1 in gastric cancer. Moreover, RNF112 exhibits low expression in gastric cancer tissues, and patients with low RNF112 expression had a poor prognosis. To some extent, RCM-1 inhibits the growth of tumors by facilitating RNF112 and FOXM1 interaction and subsequent FOXM1 ubiquitination.

H_2_O_2_-induced ROS accumulation increases RNF112 expression and its nuclear translocation, which induces the expression of cellular antioxidants and prevents differentiated Neuro-2a cells from oxidative-stress damage (45, 46). Moreover, RNF112 induces the inhibition of soluble TDP-43 and accelerates the clearance of insoluble TDP-43 aggregates in neurodegenerative TDP-43–related diseases by mediating its ubiquitination (38). Nonetheless, there is limited understanding of the potential effect of RNF112 on tumor progression. Here, we found that besides the brain, RNF112 is expressed in gastric tissues according to TCGA data and gastric cancer TMAs. We demonstrate that RNF112 abundance is significantly inhibited in gastric cancer tissues. Furthermore, we illustrate that RNF112 mediates the ubiquitination of FOXM1 in MGC803 and BGC823 cells, which reveals what we believe is a new mechanism for the degradation of FOXM1.

Many transcription factors were historically viewed as undruggable in conventional pharmacological investigations because of the absence of a ligand-binding domain serving as a drug target (47). Substantial efforts have been devoted to developing inhibitors of transcription factors (48), among which proteolysis-targeting chimera (PROTAC) technology is an attractive approach comprising 2 ligands targeting proteins of interest (POIs) and E3 ligase separately (49), which is more specific, and all functions of target proteins can be effectively interfered with (50). Recent studies have reported several PROTACs that directly target transcription factors for degradation. PROTAC SD-36 is designed to promote STAT3 degradation and suppress cell proliferation in lymphoma and leukemia (51). An effective CRBN-recruiting PROTAC, 17d, exerted a significant antitumor effect by functioning as a FOXM1 degrader in triple-negative breast cancer (52). Identification of the novel E3 ubiquitin ligase specifically targeting the POI is crucial for generating new PROTAC molecules. RCM-1 was initially found to be a FOXM1 inhibitor that suppressed the expression of FOXM1 by upregulating its ubiquitination level and promoting its shuttling from nucleus to cytoplasm. Its functions in antitumorigenesis have been illustrated in different cancer settings (39, 53). However, the underlying mechanism of RCM-1 inhibiting FOXM1 expression by affecting its proteasomal translocation remains unclear. In our study, we found that RCM-1 plays a PROTAC-like function by directly enhancing FOXM1 binding to its E3 ligase RNF112 to promote FOXM1 ubiquitination and, in turn, degradation.

Taken together, our results demonstrate that RNF112, a neurodevelopment-related protein, plays a promising antitumor role in gastric cancer by inducing the ubiquitination and degradation of oncoprotein FOXM1. Thus, the RNF112/FOXM1 axis not only could be considered a potential biomarker for predicting patients’ prognosis but also could be developed as a therapeutic target to combat gastric cancer.

## Methods

### Cell culture and cell lines.

The HEK293T and BGC823 cell lines were obtained from American Type Culture Collection. MGC803 cells were purchased from cell banks at Shanghai Fuheng Technology Co., Ltd. All cells were cultured in DMEM supplemented with 10% FBS and 1% penicillin-streptomycin at 37°C in 5% CO_2_.

### siRNA library screening for E3 ligase targeting FOXM1 protein.

The ON-TARGETplus siRNA library containing 386 E3 ligase siRNA pools was purchased from Dharmacon (GE Healthcare, catalog G-105635- 01; Human ON-TARGETplus siRNA Library-Ubiquitin Conjugation Subset 3-SMARTpool). HEK293T cells were plated in 96-well plates (4 × 10^3^ cells/well). The transfection reagent (Lipofectamine 3000 Transfection Reagent) was diluted in Opti-MEM and transferred to each well of the siRNA pool. After 15 minutes of incubation, the siRNA (50 nM)/Opti-MEM/Lipo3000 mixture was transferred to the HEK293T cells. At 48 hours after transfection, the cells were harvested and the lysates were used for immunoblotting analysis of endogenous FOXM1 expression. The band intensities were measured by ImageJ (NIH). The siRNA sequences for E3 ligases are provided in Supplemental Table 1.

### Western blotting and Abs.

After extracting the protein with RIPA buffer containing protease inhibitor (Roche), the determination of protein concentrations was performed with an enhanced BCA protein assay kit (Beyotime), followed by Western blotting analysis as described previously (54). Anti-HA (catalog H6908) was from Sigma-Aldrich. Anti-CKS1 (catalog sc-376663) was purchased from Santa Cruz. Anti-SKP2 (catalog A4046), anti-FN1 (catalog A16678), anti-CCNB1 (catalog A19037), and anti-H3 (catalog A22348) Abs were purchased from Abclonal. Anti-FOXM1 (catalog 20459S), anti-ZEB1 (catalog 83243SF), and anti-GAPDH (catalog 2118S) were purchased from Cell Signaling Technology. Anti-FLAG (catalog 20543-1-AP) and anti-RNF112 Ab (catalog 25165-1-AP) were purchased from Proteintech. Anti-tubulin (catalog AT819) was purchased from Beyotime.

### RNA extraction and quantitative reverse transcription PCR.

Total RNA was isolated from cells and tissues via RNAiso Plus reagent (Takara). The PrimeScript RT Reagent Kit (Takara) was used to reverse transcribe RNA to cDNA. The cDNAs were measured by quantitative reverse transcription–PCR using SYBR Premix Ex Taq II (Takara) with the ABI 7500 StepOnePlus system (Applied Biosystems). The primer sequences are provided in Supplemental Table 2.

### RNA-Seq analysis.

After extraction of total RNA of MGC803 cells, the quality of the sample was controlled using NanoDrop ND-1000 (NanoDrop). Then the poly(A) RNA was fragmented into small fragments and used to synthesize cDNA by reverse transcription. Next, the RNA strands in the DNA/RNA duplexes were replaced with DNA to form DNA/DNA duplexes. The dsDNA was digested with UDG enzyme, and PCR was performed to create a library with fragment sizes of 300 bp ± a SD of 50 bp. Finally, the library was subjected to paired-end sequencing using an Illumina NovaSeq 6000 (LC-Bio Technology CO., Ltd.) with a sequencing mode of PE150.

### Co-IP.

Co-IP was performed as previously described (55). Briefly, the cells were harvested with EBC buffer (50 mM Tris, pH 7.5, 120 mM NaCl, 0.5% NP-40) supplemented with protease inhibitors. For Co-IP analysis of RNF112 and FOXM1 interaction, cells were incubated with 10 μM MG132 for 8 hours prior to harvest. Then, the lysates were incubated with anti-HA or anti-FLAG agarose beads. After 3 hours of incubation at 4°C, the beads were washed 4 times with NETN buffer (20 mM Tris, pH 8.0, 150 mM NaCl, 1 mM EDTA, and 0.5% NP-40), resolved by SDS–PAGE, and analyzed by immunoblotting.

### Generation of the CRISPR-mediated RNF112-KO cell line.

The *RNF112*-KO cell line was generated as previously described (56). The sgRNA (5′-TTGATGCGAACCAGCAGCAG-3′) was designed to target *RNF112* exon 4 using the Benchling online design website (https://www.benchling.com/crispr/) and inserted into the lenti-CRISPR-V2 vector. MGC803 cells were transfected with sgRNA, followed by puromycin selection after 48 hours of transfection. Selected single cells were then plated into 96-well plates. The genomic DNA of individual clones was isolated and used as the template to amplify RNF112 exon 4, which was subsequently subjected to sequencing analysis.

### Colony-formation assays.

We plated 600 cells in 6-well plates and incubated them for 10 days. Once colonies of appropriate size had developed, the culture medium was discarded. The fixation and staining of the colonies were performed using 4% paraformaldehyde and crystal violet. The colonies were then photographed and quantified after the plates were washed and dried.

### Invasion assays.

Transwell chambers coated with Matrigel (Millipore) were inserted in a 24-well plate with 600 μL of serum-containing medium. The indicated cells were gathered in medium devoid of serum and then seeded in chambers (3 × 10^4^ cells/chamber). After incubation for 24 hours, fixation and staining were performed using 4% paraformaldehyde and crystal violet. The cells that penetrated to the lower chambers were photographed and quantified under a microscope.

### Immunofluorescent staining.

The sections were immobilized using 4% paraformaldehyde and permeabilized for 20 minutes with 0.5% Triton X-100. Next, the sections were blocked with 5% BSA for 1 hour at room temperature and incubated with primary anti-FOXM1 (1:50; catalog sc-271746, Santa Cruz) and anti-RNF112 (1:50; catalog A15333, Abclonal) overnight at 4°C. Finally, the sections were incubated with secondary Abs (1:300; Invitrogen) for 1 hour at 37°C. Cell nuclei were stained with DAPI (Beyotime). All images were collected by confocal laser microscopy (TCS-TIV, Leica).

### Immunohistochemistry.

Paraffin-embedded tissue sections were deparaffinized and dehydrated, followed by heat-induced epitope retrieval. Next, the sections were blocked in 3% H_2_O_2_. Subsequently, the slides were exposed to primary Abs overnight at 4°C and secondary Abs (Beijing Zhongshan Golden Bridge Biotechnology Co. Ltd.) for 1 hour at 37°C. Finally, staining was performed with 3,3′-diaminobenzidine (Beijing Zhongshan Golden Bridge Biotechnology Co. Ltd.), followed by counterstaining with hematoxylin, then dehydration and mounting. Anti-FOXM1 Ab (catalog ab207298) was purchased from Abcam, anti–Ki-67 Ab (catalog AF1738) from Beyotime, anti–PCNA Ab (catalog 60097-1-Ig) from Proteintech, and anti–RNF112 Ab (catalog PA5-53402) was from Thermo Fisher Scientific.

### Luciferase reporter assays.

Luciferase reporter assays were performed as previously described (57). The pGL4.26 [luc2/minP/Hygro] plasmid was used to generate a reporter containing 6 copies of the FKH-binding consensus (5′-AAACAAACAAAC-3′) (35). For reporter assays, MGC803 or BGC823 cells were cotransfected with pGL4.26 (6×FKH) constructs and pRL-TK plasmid. Twenty-four hours after transfection, luciferase activity of cell lysis was assayed for using the Dual-Luciferase Reporter Assay System (Promega).

### Ubiquitination assays.

Ubiquitination assays were performed as previously described (56). His-ubiquitin, together with the indicated plasmids, was introduced into HEK293T cells for 36 hours, and 10 μM MG132 was added 8 hours before harvest. Buffer A was used to collect cells, which were then sonicated on ice. The lysates were incubated with nickel–nitrilotriacetic acid matrices (QIAGEN) for 3 hours at room temperature. The lysates were sequentially washed with buffer A, buffer A/TI (at a buffer A/buffer TI ratio of 3:1), and buffer TI, resolved by SDS–PAGE, and used for immunoblotting analysis.

### Network pharmacology–based analysis.

The simplified molecular-input line-entry system (SMILES) format of RCM-1 was obtained from PubChem (https://pubchem.ncbi.nlm.nih.gov/) and entered into the SuperPred database (http://prediction.charite.de) (40), the SwissTargetPrediction database (http://swisstargetprediction.ch/) (41), and the SEA database (https://sea.bkslab.org/) (42) to obtain the predictive RCM-1 targets. Then key genes in gastric cancer were retrieved from the GeneCards database (https://www.genecards.org/) (43). Common genes were obtained and then subjected to enrichment analysis. Cytoscape software (58) was used to establish component-target-disease model.

### Cell thermal shift assay.

The cell thermal shift assay was performed as previously described (59). Briefly, MGC803 cells were collected and the final concentration of the lysates was adjusted to 3 mg/mL. Next, the lysates were incubated with 10 μM RCM-1 or DMSO in a dry bath at various temperatures for 4 minutes and then analyzed by immunoblotting for FOXM1 and RNF112.

### Molecular docking and MD simulation.

We retrieved the crystal structure of FOXM1 from the Protein Data Bank (http://www.rcsb.org; ID: 3G73) and modeled the 3-dimensional structure of RNF112 (1–147 aa) by Modeller 10.3 (https://salilab.org/modeller/10.3/release.html). Then, we constructed the FOXM1 and RNF112 complex by ZDOCK and the rational conformation was validated by Ramachandran plots. Then, a 100-ns MD simulation was performed using the AMBER16 package with ff14SB force field (60, 61). The stable conformation of the complex was achieved while the RMSD fluctuated stably for more than 20 ns. Two ligands (RCM-1) formed a complex with the stabilized FOXM1/RNF112 complex (named the FOXM1-RCM-1/RNF112-RCM-1 complex) were prepared using the Surflex-dock module of SYBYL-2.0 (Tripos Inc.). Then, MD simulation was performed as described above except for the GAFF force fields applied to RCM-1. For the latest 20-ns sampled conformations, we calculated their binding free energy and performed energy decomposition using MM/PBSA and MM/GBSA, respectively (62). The binding visualization was generated using PyMOL 2.4 (Schrodinger, LLC).

### Survival analysis.

The cutoff value was determined by maximizing the sum of sensitivity and specificity according to ROC curves, which were applied to determine the high or low expression of FOXM1 and RNF112. The analysis was performed as previously described (63).

### Animal experiments.

Nude mice (male, 6 weeks old) were obtained from Huafukang Biotechnology Company. MGC803 cells (2 × 10^6^; stable RNF112 overexpression or RNF112-KO cell lines) were collected in 100 μL of PBS and inoculated into nude mice s.c. (*n* = 5). Once palpable tumors were established, tumor sizes were monitored every 2 days. The following formula was used to calculate the tumor volume: volume = length × width^2^ × 0.5. On day 7 after injection, mice in the RCM-1 treatment group were administered either RCM-1 (20 mg/kg) or DMSO i.p. every 3 days until the sacrifice day. On day 19, tumors were removed from sacrificed mice and used for subsequent analysis. For the tumor invasion assays in vivo, 2 × 10^6^ stable–empty vector (stable-EV) and stable-RNF112 MGC803 cells infected with lentivirus encoding luciferase, were injected through the tail vein into male nude mice aged 4 weeks. The tumor invasion was monitored by bioluminescence imaging.

### Multiplexed immunofluorescent staining.

A gastric cancer TMA containing 110 cases of gastric cancer and paired, adjacent, noncancerous tissue was purchased from Shanghai Outdo Biotech. Multiplexed immunofluorescent staining of TMA was performed using the Opal 7-color Manual IHC Kit (PerkinElmer). Briefly, the slides were processed sequentially as follows: deparaffinization in xylene and ethanol, antigen repair by microwave, incubation with blocking buffer for 10 minutes, primary anti-FOXM1 (1:50; catalog ab207298, Abcam) and anti-RNF112 (1:50; catalog PA5-53402, Thermo Fisher Scientific) for 60 minutes, secondary Ab for 10 minutes, and Opal dyes for 10 minutes at room temperature. Microwave treatment was used to remove the Ab before proceeding with the next round of staining. Last, the slides were mounted with VECTASHIELD HardSet Antifade Mounting Medium with DAPI and scanned using confocal microscopy. Mean fluorescence intensities and percentage of positively staining cells were measured by TissueGnostics StrataQuest 7.0.1.165. Two pathologists who were blinded to the clinicopathological characteristics confirmed the results independently.

### Statistics.

Normality testing was performed by the Shapiro-Wilks test. The correlation between variables was assessed by Pearson’s correlation analysis. The Kaplan-Meier method was used in survival analyses. Two-tailed Student’s *t* test (2 groups) and 1-way ANOVA (multiple groups) test coupled with Tukey’s post hoc test were used to compare variables with a normal distribution, and the Mann-Whitney *U* test was used to compare those with a nonnormal distribution. Statistical significance was set at *P* less than 0.05.

### Study approval.

The study was approved by the Ethics Committee of the Second Affiliated Hospital of Third Military Medical University and by the Shanghai Outdo Biotech Company. All animal experiments were performed in accordance with protocols approved by the Laboratory Animal Welfare and Ethics Committee of the Second Affiliated Hospital of Third Military Medical University (approval no. AMUWEC20213581).

### Data availability.

RNA-Seq data can be accessed in the NCBI GEO via GSE197571 (https://www.ncbi.nlm.nih.gov/geo/query/acc.cgi?acc=GSE197571).

## Author contributions

CH and SY designed the experiments and revised the manuscript. The order of co–first authors SZ and JW was determined by their contribution to the article. SZ conducted most of the experiments, wrote most of the manuscript text, and analyzed the data. JW conducted some experiments and wrote the figure legends. WH, LH, QT, JL, MJ, XL, and CL participated in some experiments. QO performed molecular docking. All authors read and approved the final manuscript.

## Supplementary Material

Supplemental data

Supporting data values

## Version 1 06/08/2023

Electronic publication

## Version 2 06/12/2023

Authorship note addition: SY and CH are co-senior authors.
