# Supplementary material for: RNF112-mediated FOXM1 ubiquitination suppresses the proliferation and invasion of gastric cancer
Source: JCI Insight. 2023 Jun 8;8(11):e166698. doi: 10.1172/jci.insight.166698 (PMC10259508; doi:10.1172/jci.insight.166698)

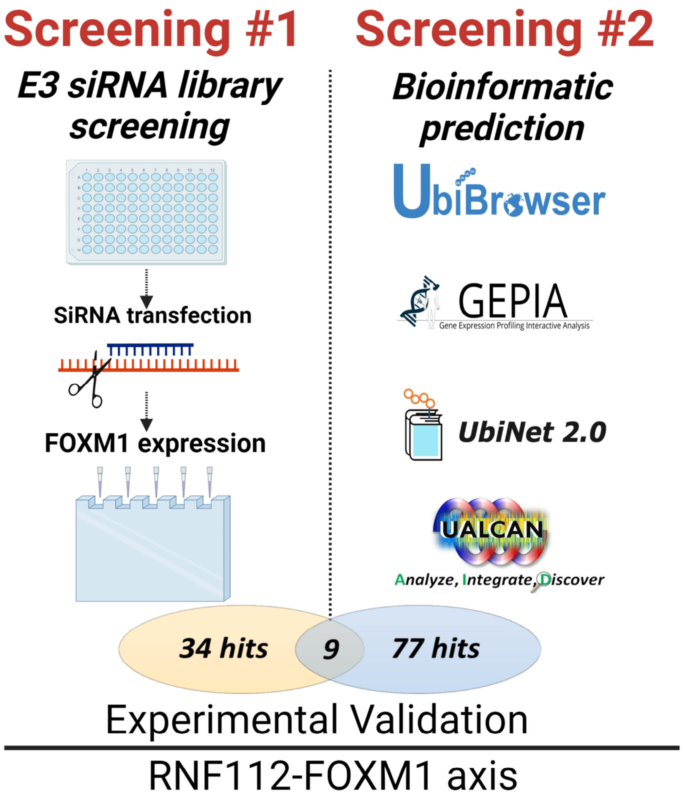

Supplemental Figure 1. Workflow of the identification of novel E3 ubiquitin ligases targeting FOXM1 degradation.

1

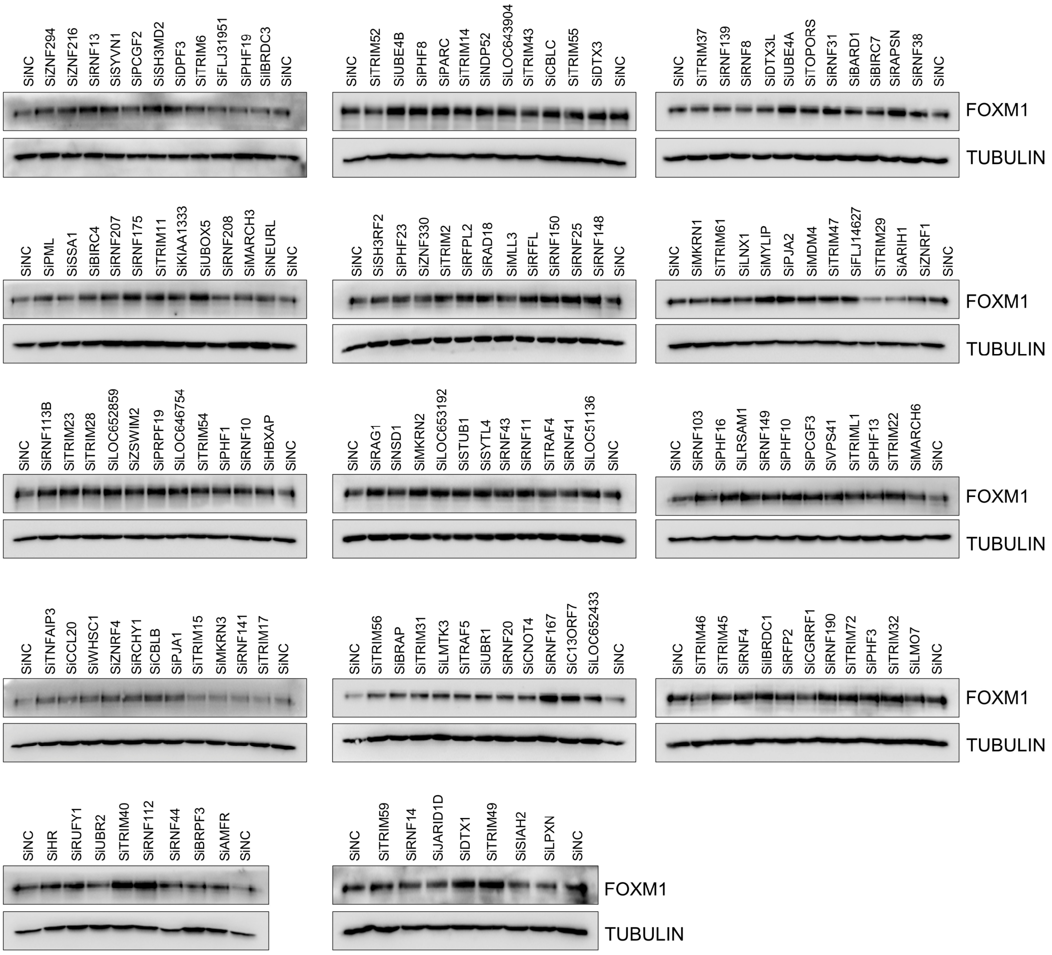

Supplemental Figure 2. Representative immunoblot results of FOXM1 after the transfection of siRNA library targeting E3 ligases in HEK293T cells. Western blot analysis of FOXM1 in HEK293T cells after the knockdown of indicated E3 ligases by siRNA transfection. After HEK293T cells seeded in 96-well plates, The mixture of ON-TARGET plus siRNA library (final siRNA concentration of 50 nM) and Lipofectamine 3000 together with Opti-MEM was added into to each well. The lysates were harvested for immunoblot analysis for endogenous FOXM1 expression 48 h later. Only those with apparent changes were listed.

2

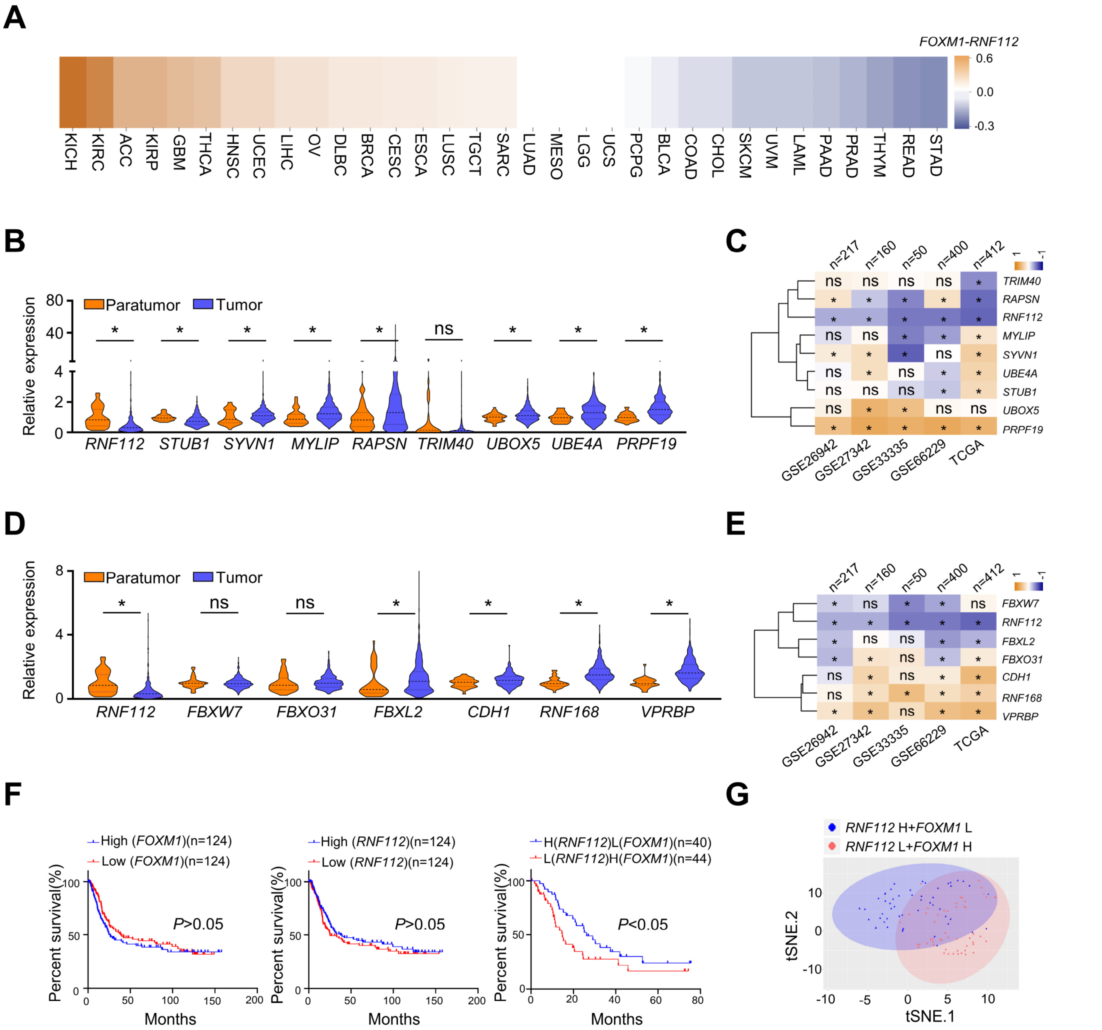

Supplemental Figure 3. The bioinformatic screen for E3 ligases targeting FOXM1. (A) The correlation between *FOXM1* and *RNF112* in 33 types of cancer tissues in TCGA. The color gradient depicts the strength of the correlation. **(B)** Violin plot of the expression of nine potential E3 ligases targeting FOXM1 in gastric cancer and adjacent tissues from TCGA (n=412). **(C)** Heatmap of the correlation between nine potential E3 ligases and *FOXM1* in gastric cancer tissues from five independent cohorts of TCGA and GEO. Orange represents a positive correlation, and blue represents a negative correlation. **(D)** Violin plot of the expression of *RNF112* and six reported E3 ligases targeting FOXM1 in gastric cancer and adjacent normal tissues from TCGA (n=412). **(E)** Heatmap of the correlation between the above E3 ligases and *FOXM1* in five independent gastric cancer cohorts. **(F)** Kaplan–Meier survival curves of gastric patients based on the expression of *FOXM1* and *RNF112* in the GSE15460 cohort. **(G)** tSNE cluster map showing subclusters of gastric cancer patients based on *FOXM1* and *RNF112* expression in GSE15460 cohort. Statistical significance was calculated using Mann–Whitney U test (B, D), Pearson's test (C, E), log-rank test (F). **P*< 0.05.

3

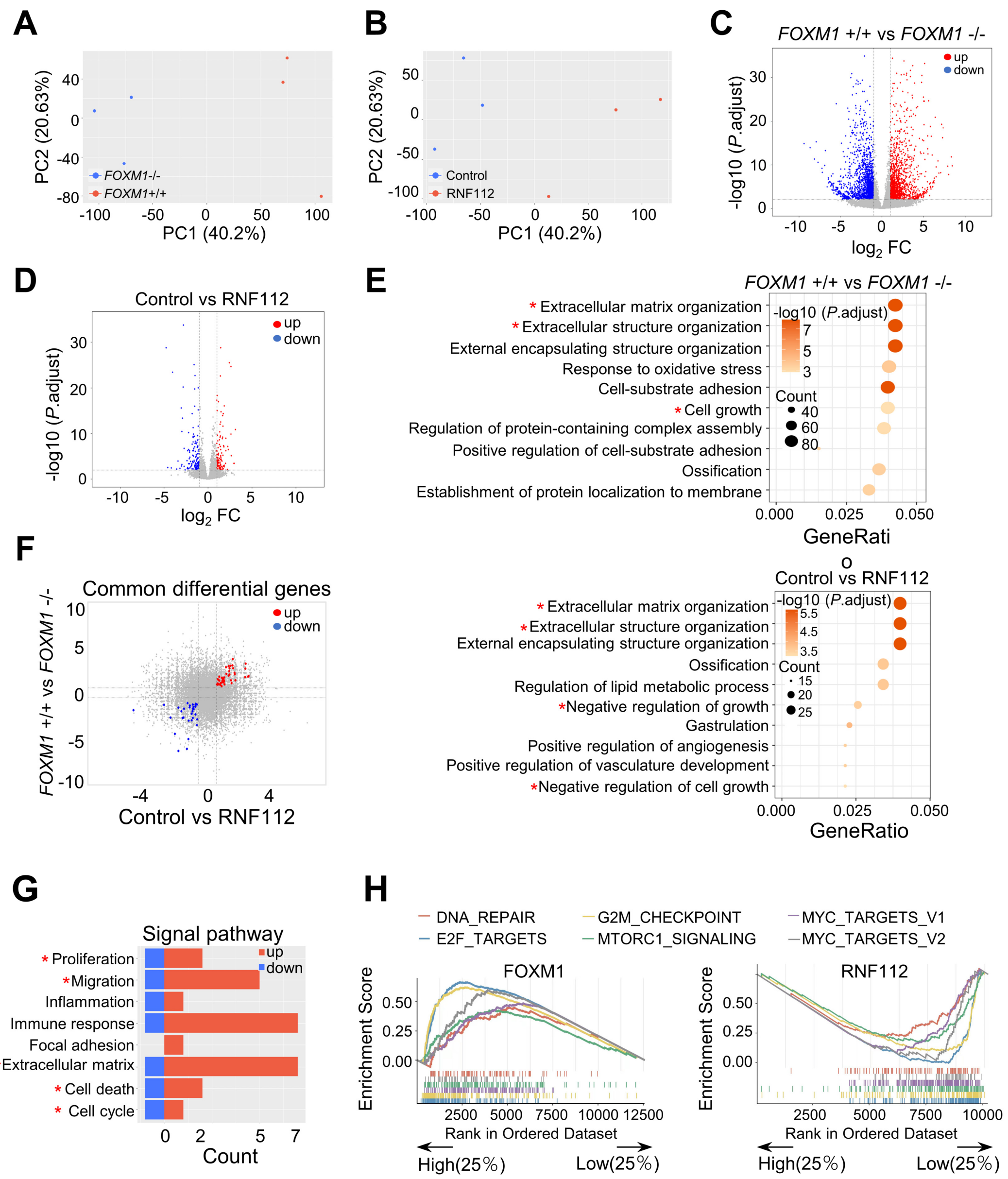

Supplemental Figure 4. FOXM1 and RNF112 are both associated with the proliferation and invasion signaling pathways in gastric cancer. (A-B) PCA analysis of RNA-Seq results of *FOXM1* knockout (A) and RNF112 overexpression MGC803 cells (B). **(C-D)** Volcano plots of differentially expressed mRNA revealed by RNA-seq in MGC803 cells between *FOXM1*+/+ and *FOXM1*-/- group (C) and control and RNF112-overexpressing group (D). **(E)** GO enrichment analysis of biological processes of significantly changed genes from (C-D). **(F)** Scatter plot of common differentially expressed gene between the above two indicated groups. The genes with similar remarkable changes are highlighted. **(G)** GO enrichment analysis of pathways from highlighted genes in (F). **(H)** GSEA analysis based on the expression of *RNF112* and *FOXM1* in TCGA gastric cancer dataset.

4

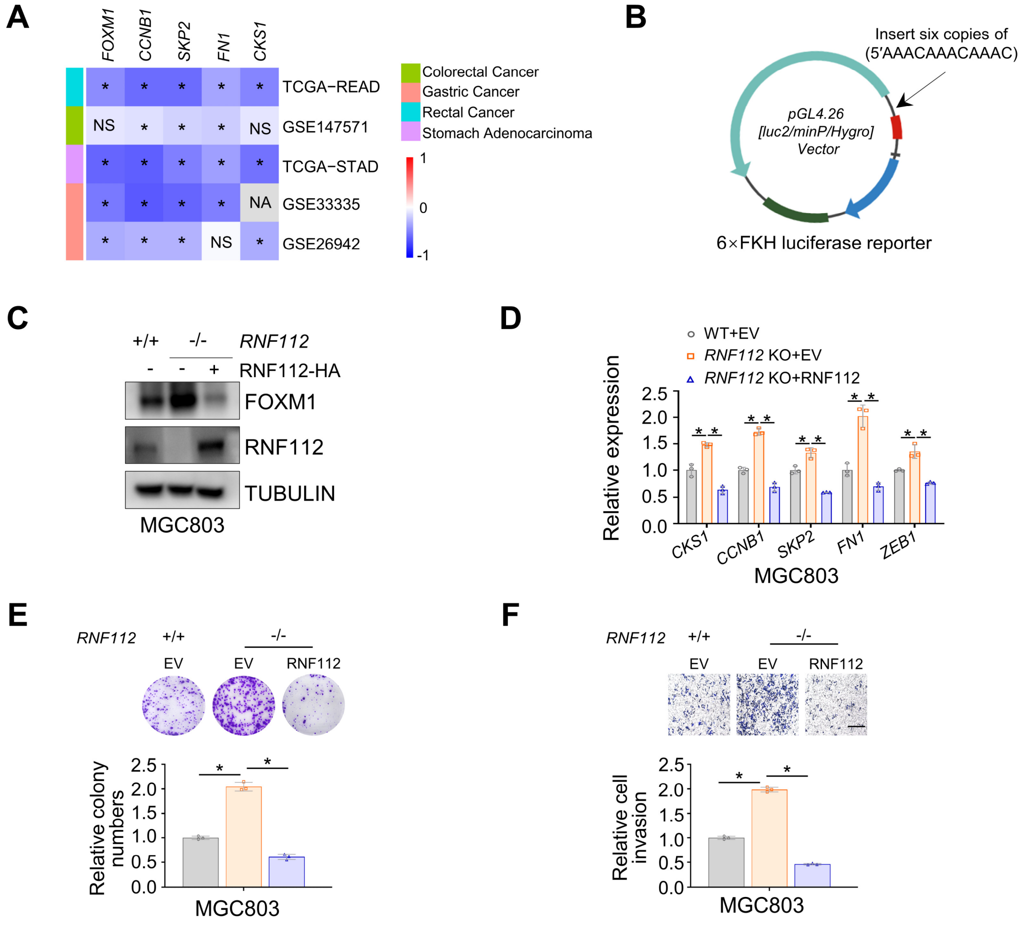

Supplemental Figure 5. RNF112 suppresses FOXM1 downstream genes. (A) The correlation between *RNF112* and *FOXM1*, *CCNB1*, *SKP2*, *FN1*, *CKS1* in several online datasets derived from TCGA and GEO. **(B)** Schematic illustration for the 6×FKH luciferase reporter plasmid containing six copies of the Forkhead consensus sequence (AAACAAACAAAC). **(C-F)** Immunoblot analysis (C), qRT-PCR analysis (D), colony formation assays (E), transwell invasion assays (F) of *RNF112*-depleted MGC803 cells infected with RNF112 lentivirus (n=3). Scale bar, 400 μm. Data are presented as mean±SD. Statistical significance was calculated using Pearson's test (A), one-way ANOVA (D-F)**.** NS, not significant, NA, not available, *$P< 0.05$.

5

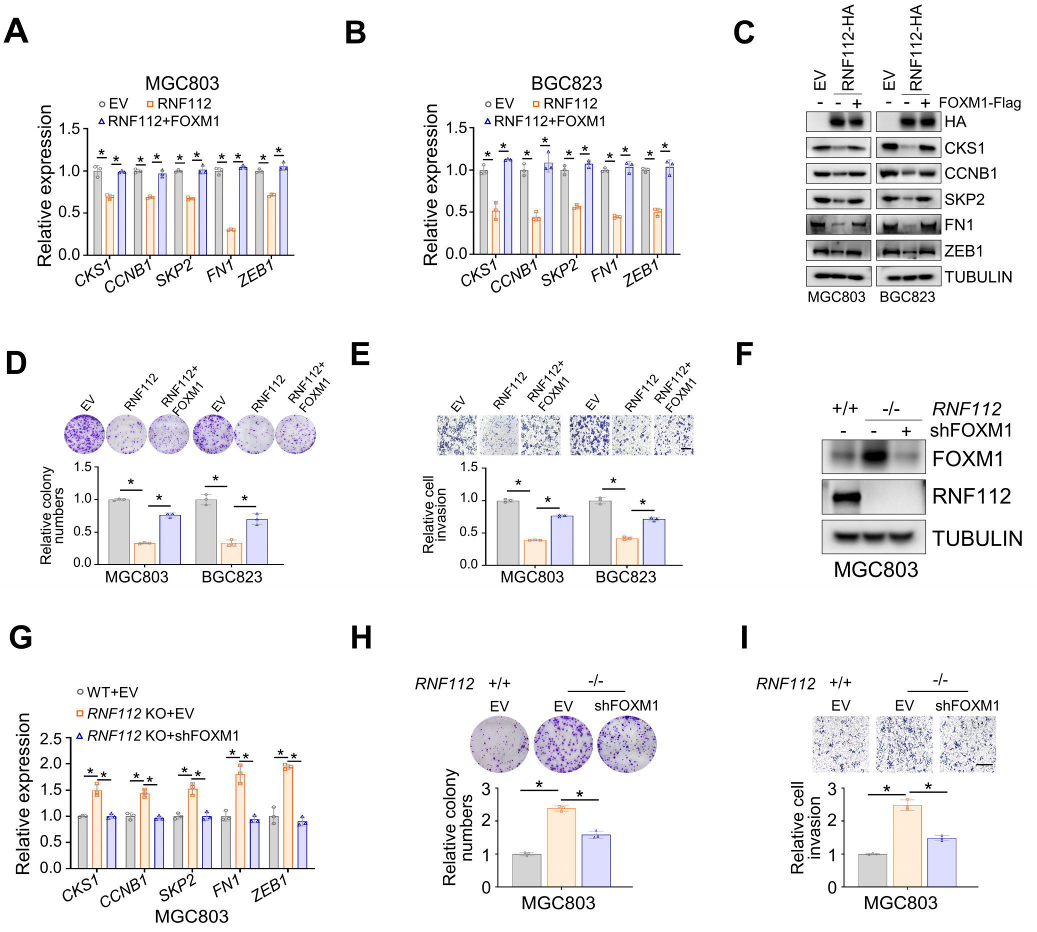

Supplemental Figure 6. Anti-tumor effect of RNF112 is partially owed to FOXM1 degradation. (A-C) qRT-PCR analysis (A-B) and immunoblot analysis (C) of the expression of FOXM1 downstream genes in RNF112 stably overexpressing MGC803 and BGC823 cells after the restoration of FOXM1 (n=3). **(D-E)** Colony formation assays (D) and transwell invasion assays (E) of RNF112 stably overexpressing MGC803 and BGC823 cells after the restoration of FOXM1 (n=3). Scale bar, 400 μm. **(F-I)** Immunoblot analysis (F), qRT-PCR analysis (G), colony formation assays (H), transwell invasion assays (I) of *RNF112* depleted MGC803 cells with FOXM1 knockdown (n=3). Scale bar, 400 μm. Data are presented as mean±SD. Statistical significance was calculated using one-way ANOVA. **P*< 0.05. Complete unedited blots were listed in the supplemental material.

6

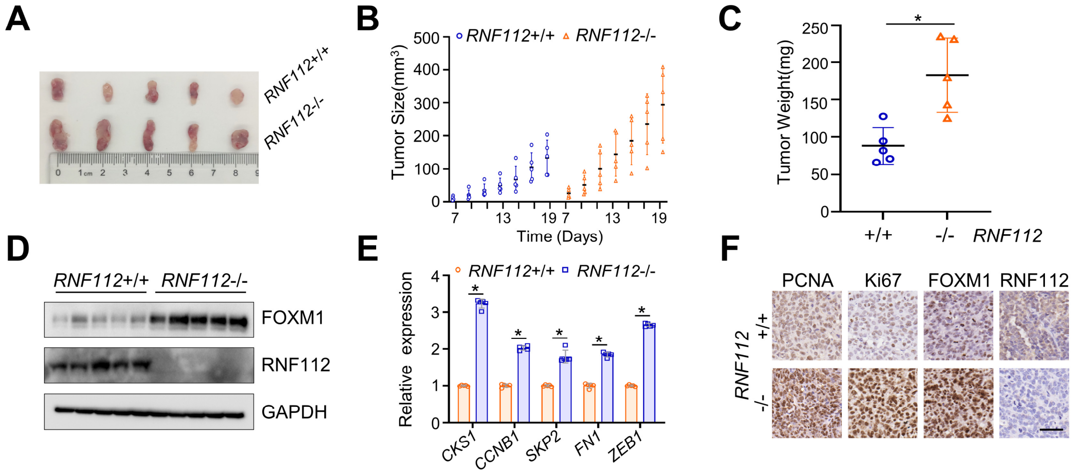

Supplemental Figure 7 *RNF112* depletion promotes gastric cancer cell proliferation in vivo. (A) Tumors were harvested after subcutaneous injection of wild-type or *RNF112*-depleted MGC803 cells in nude mice (n=5). **(B-C)** Tumor volume (B) and tumor weight (C) of the xenograft were calculated (n=5). **(D-F)** Immunoblot analysis (D), qRT–PCR (E) analysis and IHC staining (F) of tumors from the above mice (n=5). Scale bar, 50μm. Data are presented as mean±SD. Statistical significance was calculated using Student's t test (C, E). **P*< 0.05. Complete unedited blots were listed in the supplemental material.

7

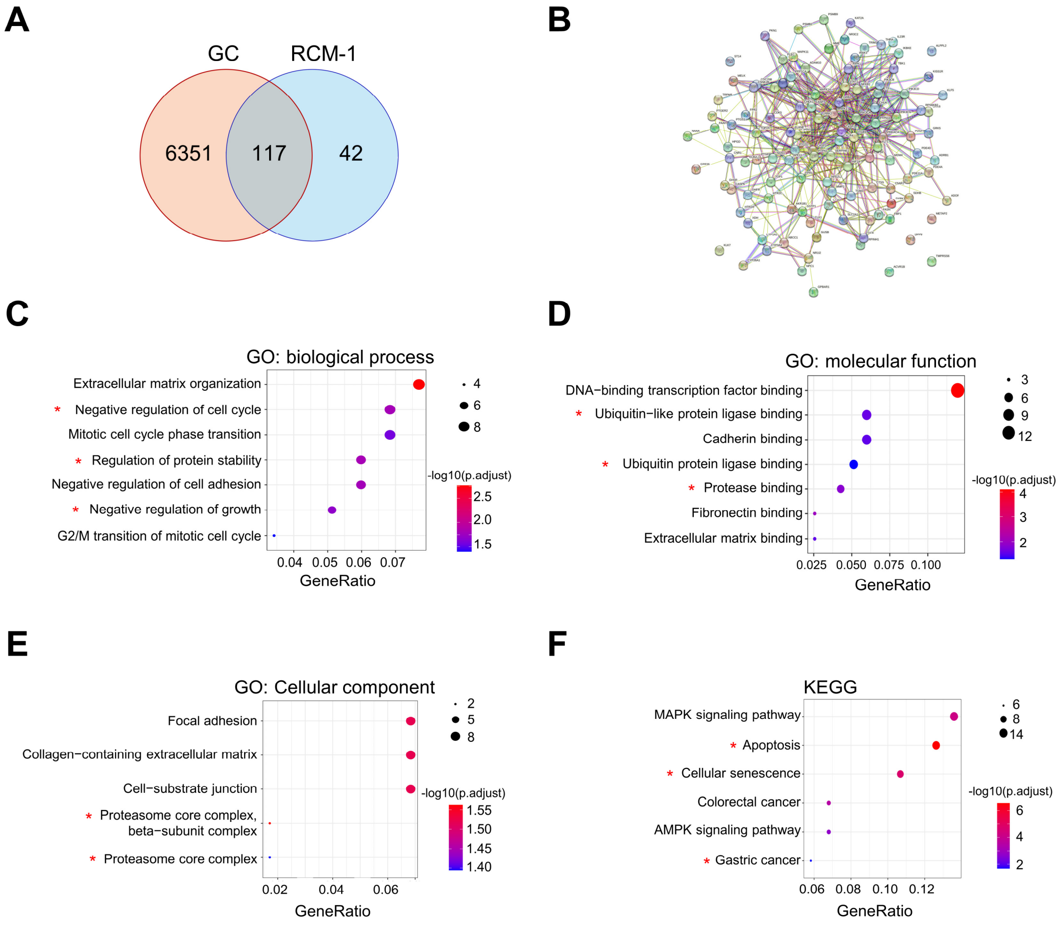

Supplemental Figure 8. Network pharmacology analysis of the role of RCM-1 in gastric cancer. (A) Venn diagram of common RCM-1 targets obtained from SuperPred database, Swiss database and SEA database and key genes of gastric cancer retrieved from GeneCards. **(B)** A component-target-disease model established by Cytoscape software. **(C-F)** GO biological process analysis (C), GO molecular function analysis (D), GO cellular component analysis (E), and KEEG pathway analysis (F) of the common genes in (A).

8

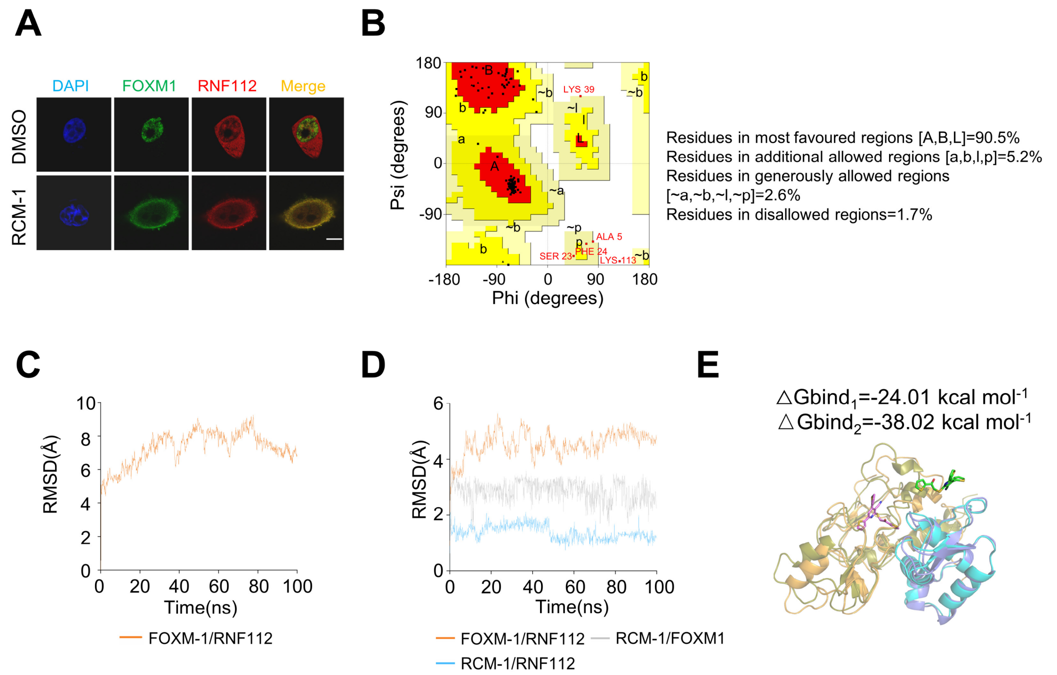

Supplemental Figure 9. Molecular docking and molecular dynamics simulation analysis of FOXM1, RNF112 and RCM-1. (A) Immunofluorescent analysis of cellular distribution of FOXM1 and RNF112 in MGC803 cells after DMSO or RCM-1 treatment (10 μM). Scale bar, 10μm. **(B)** Ramachandran plot analysis of φ and ψ dihedral angles of the model of RNF112. **(C)** RMSDs of FOXM1/RNF112 complex for 100ns MD simulations. **(D)** RMSDs of FOXM1-RCM-1/RNF112-RCM-1 complex for 100ns MD simulations. **(E)** Comparison of binding modes and binding free energies of FOXM1/RNF112 complex (FOXM1, light blue; RNF112, olive; $\triangle G_{bind1}$) with those of FOXM1-RCM-1/RNF112-RCM-1 complex (FOXM1, cyan; RNF112, orange; RCM-1/FOXM1, green; RCM-1/RNF112,violet; $\triangle G_{bind2}$).

9

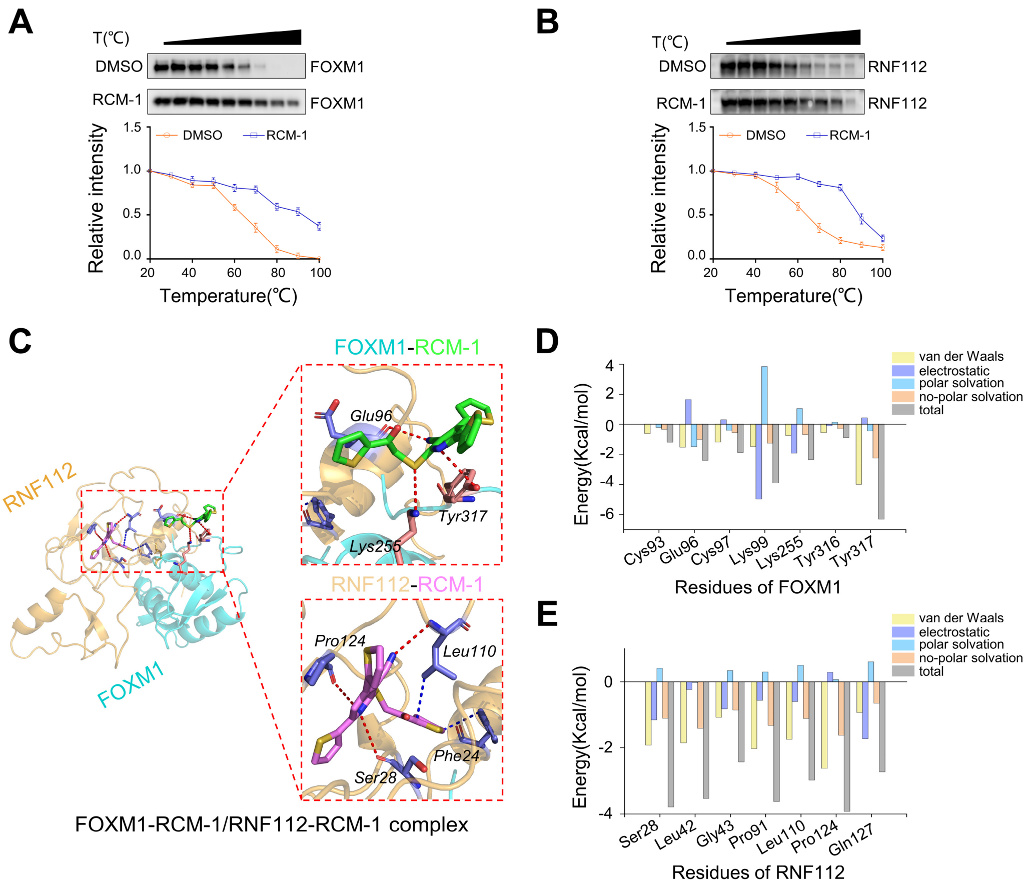

Supplemental Figure 10. Possible binding modes among FOXM1, RNF112 and RCM-1. (A-B) Thermal shift assays of MGC803 cells in the presence of DMSO or RCM-1 (10 μM). MGC803 cells were lysed using RIPA buffer containing protease inhibitor and the final concentration of the lysates was adjusted to 3mg/ml. The lysates were heated in the presence of 10 μM RCM-1 or DMSO at various temperatures for 4 min and then subjected to immunoblot analysis. **(C)** Docking model of FOXM1-RCM-1/RNF112-RCM-1 complex. Red and blue dashed lines indicate hydrogen bonds and hydrophobic interaction respectively. **(D-E)** Contributions of binding free energies calculated by MM/GBSA method for key residues of FOXM1/RNF112 complex with RCM-1(FOXM1) (D) and RCM-1(RNF112) (E). Data are presented as mean ± SD. Complete unedited blots were listed in the supplemental material.

10

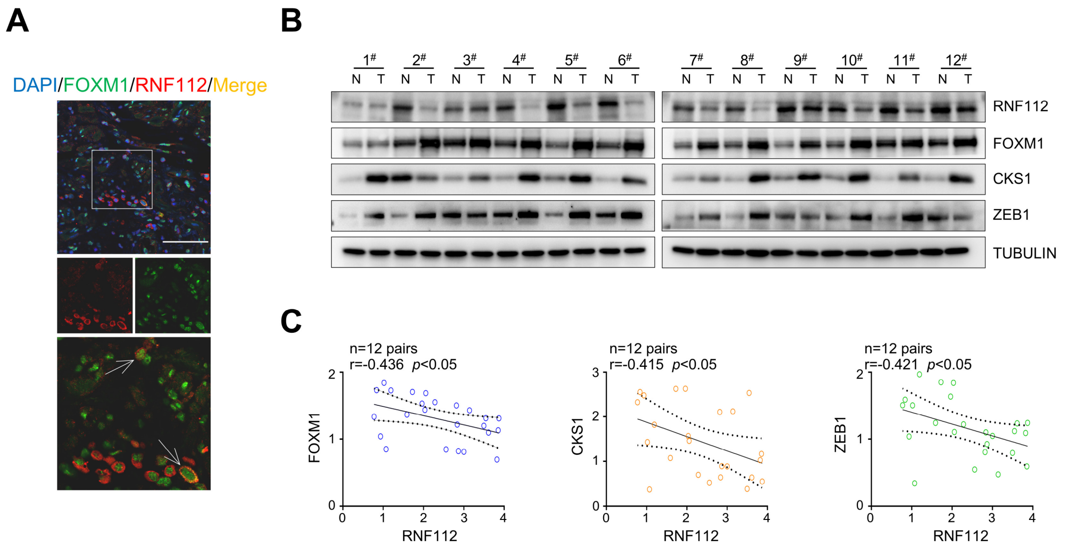

Supplemental Figure 11. RNF112 is negatively correlated with FOXM1 in gastric cancer tissues. (A) Immunofluorescence of the colocalization of FOXM1 and RNF112 in gastric cancer tissues. White arrow indicates the colocalization. Scale bar, 100 μm. **(B)** Immunoblot analysis of the protein expression of FOXM1, RNF112, CKS1, and ZEB1 in 12 pairs of gastric cancer tissues (T) and corresponding non-cancerous tissues (N) (n=12 pairs). **(C)** The correlation between expression of RNF112 and that of FOXM1, CKS1, and ZEB1, respectively (n=12 pairs). Statistical significance was calculated using Pearson's test (C). Complete unedited blots were listed in the supplemental material.

11

Supplementary Table 1 The List of siRNA Sequences

Genesymbol	GENEID	GINumber	Sequence
MDM2	4193	46488908	GAAUUUAGACAACCUGAAA
MDM2	4193	46488908	GAUGAGAAGCAACAACAUA
MDM2	4193	46488908	GCCAGUAUAUUAUGACUAA
MDM2	4193	46488908	GAACAAGAGACCCUGGUUA
PHF7	51533	27894286	GUCCAAGGGUGUCAGAAUC
PHF7	51533	27894286	GAACCUUCCUUAUUAGAAA
PHF7	51533	27894286	GAUCAGUGCCUCAGAAACU
PHF7	51533	27894286	CAGCAAAGCAUUUCUUCAA
TRIM39	56658	25777697	GCAACUAGGCAGUAUGGUG
TRIM39	56658	25777697	GAAGAAUGCUGCACCACUU
TRIM39	56658	25777697	UCGAGAUGCUUAAGGAUGU
TRIM39	56658	25777697	UUAAACAGCUAAUUGCGGA
TRIM41	90933	42516573	CCAAUAUGGUCCAGGUGAU
TRIM41	90933	42516573	CAAUAGGUGUGAAGAGGUA
TRIM41	90933	42516573	UAGCUUCACUUGAGAGAGA
TRIM41	90933	42516573	GAGAUGAGUUAGAUCGGGA
RNF133	168433	21040268	GAGCAAGUGUUGUUUGGAU
RNF133	168433	21040268	GGUUGUACCUUCACACAGA
RNF133	168433	21040268	CUAAUGACAUAGUUCGUAU
RNF133	168433	21040268	GAGAGUGGCAGGAGUUAUA
KIAA1718	80853	90093354	UGAUGGAUGUGGAACGUUA
KIAA1718	80853	90093354	CUAUAAACAACCCGUGUAA
KIAA1718	80853	90093354	CCUAGUACAGGGAGUGAAA
KIAA1718	80853	90093354	GUACCUGAAUGGAGAGCGA
RNF32	140545	37595535	AAAUAAGGGUCACUCAUCU
RNF32	140545	37595535	AGUCAAUGCAGUUGCUUUA
RNF32	140545	37595535	GAUCAGUGCUUGGCCAUAA
RNF32	140545	37595535	GAGAGGAUGUGUUGUUAGA
INTS12	57117	21361850	GUCAGUAACUAGUGGCUUA
INTS12	57117	21361850	GCUCAAUGCUAUGAAGCGA
INTS12	57117	21361850	UGUCAAAGAUCCAUUGGUU
INTS12	57117	21361850	GAAGUAAUGUGGCCAAGUA
ZNF592	9640	7661999	AGUCAUUUGAAGCGGAGAA
ZNF592	9640	7661999	UGGAAGAAUGUGCCGGUGA
ZNF592	9640	7661999	CAUCAAAGAUAACGGAUUU
ZNF592	9640	7661999	CAGCAAAGGCUCACCGUCU
LOC92312	92312	88952495	GGGCAACCAUCAAGCGCAU
LOC92312	92312	88952495	GAGCGAGACAGGCGACCAA
LOC92312	92312	88952495	GCAUUAUAUUUAGGGAGUA
LOC92312	92312	88952495	GCAAAAUAAAAGCGGGAAA
PRPF19	27339	34222313	GUACUAAUGUGGCCAACUU
PRPF19	27339	34222313	GAUCUGCGCAAGCUUAAGA
PRPF19	27339	34222313	GAUAACAACUUUGAGGUAA
PRPF19	27339	34222313	GCACGGAUGUCCAGAUCUA
RBX1	9978	22091459	GGGAUAUUGUGGUUGAUAA
RBX1	9978	22091459	GAAGCGCUUUGAAGUGAAA
RBX1	9978	22091459	CAUAGAAUGUCAAGCUAAC
RBX1	9978	22091459	GGAACCACAUUAUGGAUCU
LOC653111	653111	113422565	CCAUCAGGUUUCAUUGUAU
LOC653111	653111	113422565	AAUCUAUCCUUUGAGGUAA
LOC653111	653111	113422565	AGUGAAGUCUCAAUGAUAA
LOC653111	653111	113422565	CGCUAAAAGUAUACGUAUU
HRC	3270	75905816	ACACAGUGGUCAAGGAUAG
HRC	3270	75905816	GGUCAAAGAGGGUCCAUCA
HRC	3270	75905816	GCACCAAGGCCACGGGAUU
HRC	3270	75905816	CAAGGAAUAUGGGCACCUA
MYCBP2	23077	56118211	GAAUAGUUGUGGUUGGUUU
MYCBP2	23077	56118211	GCAAGGAUUUGGUGUGUGU
MYCBP2	23077	56118211	GGAAGUAGUUCUGUUAUUU
MYCBP2	23077	56118211	GCAGAAAUGCCCACACUUU

PRICKLE1	144165	23308518	GAUGAUGAUUCCUGGUGUU
PRICKLE1	144165	23308518	GAGAGAAGCAUCGGAUUAA
PRICKLE1	144165	23308518	CCAAUGAGAUGGAUAUUCG
PRICKLE1	144165	23308518	GAACAAGUUUUGCCAGUGA
CBL	867	52426744	UAGCCCACCUUAUAUCUUA
CBL	867	52426744	GGAGACACAUUUCGGAUUA
CBL	867	52426744	AAUCAACUCUGAACGGAAA
CBL	867	52426744	GACAAUCCCUCACAAUAAA
CHD5	26038	24308088	CACCGAAUCCUGAACCAUA
CHD5	26038	24308088	CGGGAGGGAUUGUCAACUA
CHD5	26038	24308088	AGGAUGAGCUCUCUGAUAA
CHD5	26038	24308088	GGAAAGACCUGCCCUACGA
RNF180	285671	31341781	CCAAGAGCCGGACUGAUUA
RNF180	285671	31341781	CAAGUAUUCUUCGUUGUUG
RNF180	285671	31341781	AAACAUGGCCCGAAAUAAU
RNF180	285671	31341781	GAAUGGAUAAGCUGCCUAA
PHF21A	51317	19923461	GACACUACCUCUCGUCUUG
PHF21A	51317	19923461	AAGAGUAGUUGAACAGCUA
PHF21A	51317	19923461	GCAAUUCCAUAAGUAAAUG
PHF21A	51317	19923461	GCAGUGACAUACCUAAACA
TRIM6-TRIM34	445372	51477689	CGAUUUAGUGUCUGGAACA
TRIM6-TRIM34	445372	51477689	GCCCAAAGCCUGUUAGCCA
TRIM6-TRIM34	445372	51477689	AGAACAUAUUCCCGCCAUA
TRIM6-TRIM34	445372	51477689	CUAUUUGGCUACUGGGUUA
LOC642678	642678	113413044	AGCAACAUCUGCAGGUCUA
LOC642678	642678	113413044	GUGUGAUGAUUGUGACAUA
LOC642678	642678	113413044	GGUUGGAGGUGUCUUGAGU
LOC642678	642678	113413044	AAGUGCAAAUGGUGUGUUU
MARCH8	220972	50539413	GGAAGAGACUCAAGGCCUA
MARCH8	220972	50539413	UAAAGUGUAUGUGCAAUUG
MARCH8	220972	50539413	GAAUGGCCCUUUUGGACUA
MARCH8	220972	50539413	GAGCAGAAAUCAUUCACGU
PHF20L1	51105	39653322	GCGCUAACAGCAAUAAAGA
PHF20L1	51105	39653322	GCAAAGAGAGCUCGACUUA
PHF20L1	51105	39653322	CAGAGAGCACAUUAUCAAA
PHF20L1	51105	39653322	CCACACCAGACGUAGAGAA
RNF152	220441	27734872	GAUAUCCUGUGGCUGAAGA
RNF152	220441	27734872	CAAGAAAGGUGGCAAGGUA
RNF152	220441	27734872	CACAACAUGUCUUGCAUUU
RNF152	220441	27734872	GGUGUGCACUGUCAUCUUG
RFWD2	64326	50428921	GCUAAUGUGUGCUGUGUUA
RFWD2	64326	50428921	CUACAAGGAUGUCUCGUAU
RFWD2	64326	50428921	CAUAAGAACCUGUUAGCUA
RFWD2	64326	50428921	GAAUUGGUAUGAAGGGUUA
RSPRY1	89970	45387948	CAGUAUUAGUGACCGGCUU
RSPRY1	89970	45387948	GCAAUGAUGUCAGCGAGUA
RSPRY1	89970	45387948	CAAGUCAGUAUCCGAGAAA
RSPRY1	89970	45387948	CUGAUUAUCUGAAACGUCA
MID2	11043	16445408	GGUCAACAGUCCUCAUCAA
MID2	11043	16445408	GCAAAUGAUCGCUGUCAAA
MID2	11043	16445408	GCGCAACAGCGAACUAGAA
MID2	11043	16445408	AAUCAAAUGGCCAAACUAA
MLLT6	4302	57222567	CAUGAUCGCUUCAACAAGA
MLLT6	4302	57222567	GCUCGUCGCUGUCCUUCCA
MLLT6	4302	57222567	GUAGGAGGCUGCUGCGUAU
MLLT6	4302	57222567	AGCCAUAGCCUGAGUCAUA
ZMYND11	10771	47078242	ACAAUAAACACCCGAUGUA
ZMYND11	10771	47078242	GCAAAGAAAGGACGACGUA
ZMYND11	10771	47078242	CGAGGAUGCUAUAUAAAGA
ZMYND11	10771	47078242	GGAGAGGGCUAUAGAUCUU
BIRC3	330	33946284	GUUCAUCCGUCAAGUUCAA
BIRC3	330	33946284	CUAACUGCCGGAAUUAUUA
BIRC3	330	33946284	CAUGUGAACUGUACCGAAU

BIRC3	330	33946284	UAACGAAAAUGCCAGAUUA
MIB2	142678	21264329	UGGCGGAGUUUAUCGGACA
MIB2	142678	21264329	CCUCAAGGGUCACGCGCUA
MIB2	142678	21264329	GCGAGUGGACGGACGACAU
MIB2	142678	21264329	GCUAGAGGCCAGUCCCAAA
TRIM62	55223	8922647	AGACCAACCUCACAUAUGA
TRIM62	55223	8922647	GACCAAGUCUUCCACCAAG
TRIM62	55223	8922647	CUACAAUGCUGAUGACAUG
TRIM62	55223	8922647	GCGAGAAGUUCCCUGGCAA
RNF125	54941	37595554	UUAGACCGGUCACUUCUUG
RNF125	54941	37595554	GAAAUGAGGGCACAUAUUC
RNF125	54941	37595554	UUUAAUACCCGAUGAGAAU
RNF125	54941	37595554	GCAGCAAGGUGUGUAUGUC
BMI1	648	39725706	GGUUCGACCUUUGCAGAUA
BMI1	648	39725706	CCUCGAAAAUCAUCAGUAA
BMI1	648	39725706	GAAAUUCAACCAACGGAAA
BMI1	648	39725706	ACAUAAGCAUUGGGCCAUA
PHF20	51230	40804464	CUGGAGUGAUGAUGAGUAU
PHF20	51230	40804464	AGACAUUGACUACGAGGAA
PHF20	51230	40804464	GAGGAUAAUUUGAGUGAGU
PHF20	51230	40804464	GCGUGAUGAUUGUUGUAUU
ZNF313	55905	40807455	GGAACACUGCAAAUUAUUC
ZNF313	55905	40807455	GUGUGAAGGCCACCAUUAA
ZNF313	55905	40807455	GCUUAGAGGUGUACGAGAA
ZNF313	55905	40807455	GCACGGAUACCAAAUCUGU
RNF185	91445	31542782	GGCCAGAGCCGGAGAAUAG
RNF185	91445	31542782	GCCACAGCAUUUAAUAUAA
RNF185	91445	31542782	UCACGCCUCUUCCUAUUUG
RNF185	91445	31542782	GGUGUGUCCUGUUUGCAAA
ZNRF3	84133	51476052	GAAAUUGGGAACUGUAUGG
ZNRF3	84133	51476052	GCAGAUCUUAGGUUAGGUA
ZNRF3	84133	51476052	UCAAGAGGCCGGUGGUGUA
ZNRF3	84133	51476052	UCACUUCUGUCAUCCUAUU
JARID1B	10765	57242795	UAAGUUAGUUGCAGAAGAA
JARID1B	10765	57242795	GGAGAUGCACUUCGAUAUA
JARID1B	10765	57242795	GGAAGAUCUUGGACUUAUU
JARID1B	10765	57242795	UCGAAGAGAUCCCUGCAUA
RNF123	63891	37588868	CAACUGGGCCUUCUCUGAA
RNF123	63891	37588868	GCGCUACUAUUGGGAUGAA
RNF123	63891	37588868	GGUGAAGCUUCUAGGUAUA
RNF123	63891	37588868	GCACAUGGCGGACCUCCUA
PHF11	51131	7705832	UCUGAUGGAGUUCGAGGAA
PHF11	51131	7705832	GCCCCAAAGAUGUCGAAUA
PHF11	51131	7705832	CGCUCAAAGUGCUAAAUUU
PHF11	51131	7705832	ACUGUAGAUUGUUCGAAGA
TRIM60	166655	37622897	CCACAAGUAUCAAAACCUA
TRIM60	166655	37622897	GAGGAAUAAUAUAGAACGA
TRIM60	166655	37622897	GCAAUUGGGCGAUACAUGA
TRIM60	166655	37622897	GGGCAAGUCUGUGCAGUCA
TRIM8	81603	13569865	UGUACAAACUCGAGUCAGA
TRIM8	81603	13569865	GGACAACUGUUACUGUUCU
TRIM8	81603	13569865	GAGAUCCGAAGGAAUGAAA
TRIM8	81603	13569865	GCAAGAUUCUCGUCUGUUC
RNF122	79845	38045930	UGAAAUGGCUGGAAGUUCG
RNF122	79845	38045930	CAACACGCCUUUCACCGCA
RNF122	79845	38045930	AGAGUGAGCGAUACGGAUA
RNF122	79845	38045930	GAACAUUGGGAUUCUAUUG
RNF39	80352	25777717	CAUUGCAGGAGUAUUAAUA
RNF39	80352	25777717	CUACAGUACUUCCUGACUU
RNF39	80352	25777717	GGAAAGCGGCAAAAGUUAA
RNF39	80352	25777717	GUGGAGAGAUUUGACGAGA
RNF12	51132	34452683	CUGCAUCGAUCGCUGGUUA
RNF12	51132	34452683	GCAAUUCAGACCAUGUUAA

RNF12	51132	34452683	CCACGAAGAUCUCAUCAUA
RNF12	51132	34452683	CAGAGAAAGUGUUGUGUAA
RNF135	84282	37655168	UGGAACAUCUUGUAGACAU
RNF135	84282	37655168	CAUCCAACCUUUAACUUGA
RNF135	84282	37655168	UAGAGAAGAGCAUCACAGA
RNF135	84282	37655168	GGACCUGGCCGACAAGUAC
ZNRF2	223082	40316919	GUAAAUAGAUCUUGCCCUG
ZNRF2	223082	40316919	CAAUAUGCCUUGAAGAAUU
ZNRF2	223082	40316919	GCACAUGUUUGGAGGAUUU
ZNRF2	223082	40316919	GCACGACUGCCUUGUCUAU
RKHD1	399664	42558261	GAGCCGGUCUUCAUCGUGA
RKHD1	399664	42558261	GUAUUGAGUGGUCAGGUUA
RKHD1	399664	42558261	GCGACAAGGAGCCGGUGUU
RKHD1	399664	42558261	GCCGAACACUUCUCCAUCA
TRIM67	440730	52138585	UAACCUGGCUUUAAUAGUG
TRIM67	440730	52138585	GAAGGUGGCGUGUGCAAGG
TRIM67	440730	52138585	GCACAAGGCACAACUAUCU
TRIM67	440730	52138585	GGUAAGGAGACUUUGUGUA
LOC644006	644006	88943821	CCAGAAGCGAACACGGGAA
LOC644006	644006	88943821	GCAGUGACGAUGAGUUGUU
LOC644006	644006	88943821	UUGAAACCCACGUCUGAUA
LOC644006	644006	88943821	GGGCAAUCCCGUUUCCUAA
PHF6	84295	63478059	GCAUGAUAAAGCUCAAAUA
PHF6	84295	63478059	AGAUAGGUCUCCACACAGA
PHF6	84295	63478059	UGUGGACAGUUACUAAUAU
PHF6	84295	63478059	GCACGAAGCUGAUGUGUUC
TRIM63	84676	19924162	GAGGAUUCCCGUCGAGUGA
TRIM63	84676	19924162	GGAAGAAGGACACCAGUAA
TRIM63	84676	19924162	GCAAGGAGCACGAAGAUGA
TRIM63	84676	19924162	AGACUGAACUGAAUAACUG
RFPL4B	442247	61966914	AGUCUGUAGAUGCGGAUAA
RFPL4B	442247	61966914	GUAAGAAGAUCCACCACGA
RFPL4B	442247	61966914	GAUAUUAGGUUUUGUGGAU
RFPL4B	442247	61966914	AAGAAUGGCAAGUGAGCGU
PDZRN3	23024	57529736	GACACGAGCUCUCAGAUAU
PDZRN3	23024	57529736	GUGGACCUCUACAGAAUGA
PDZRN3	23024	57529736	CAACGAGUCUUUCAUUUCG
PDZRN3	23024	57529736	GCACUAGAGUAUACAAUUC
C6ORF49	29964	32171176	CCGAAAUACCGGACAACGU
C6ORF49	29964	32171176	GGAUGUCGGACUAAGCGAA
C6ORF49	29964	32171176	AGACUGGACUCGACCGAAC
C6ORF49	29964	32171176	GGGACCAAACCUCGGUGAA
PHF5A	84844	55925655	GGAACUGACUGUGAAGCGA
PHF5A	84844	55925655	UGGCACACCAUAUCGGAGA
PHF5A	84844	55925655	CCUCUUCUAUGAACGCAAA
PHF5A	84844	55925655	UGGUGUUGCCAUCGGAAGA
C20ORF18	10616	14043031	UCACAGUGCGCCCUGAUAU
C20ORF18	10616	14043031	GACAGUGGCGUCUCUCAAG
C20ORF18	10616	14043031	CCUCAUACCAGCCCGACGA
C20ORF18	10616	14043031	GGACAGUGCCUACCUCUAU
TRIM75	391714	88981293	GAGAGUAGCCCAUCGAUCU
TRIM75	391714	88981293	GAUUGAAAUUGCCAAGCUA
TRIM75	391714	88981293	GAGACCACCUUGUGCGAGA
TRIM75	391714	88981293	GGGCACAACUUCUGUCGUU
PHF17	79960	40556392	CCAGACAUGAAGAUCGAAA
PHF17	79960	40556392	GCUAUGAAGCCCACCCGUA
PHF17	79960	40556392	UGAGGAUUCUGACGACAAU
PHF17	79960	40556392	CAGCGAUGCUACGACAAUA
TRIM42	287015	29029527	GCGAUACCCAGGUGGAUGA
TRIM42	287015	29029527	GUUCAAAGCAGUCCGAUAU
TRIM42	287015	29029527	GCAACAGGCGCAUCGCUUA
TRIM42	287015	29029527	CAGAAUACGUGUUUAAAGU
BAHD1	22893	41281572	GAGCCAGCCAUCCGAAAGA

BAHD1	22893	41281572	CCGCACUAAUGGCUGGGUA
BAHD1	22893	41281572	AAAUCAGGCCCACGAAAGA
BAHD1	22893	41281572	ACACACACUCGGAGAAAGU
WDR24	84219	14149986	GUUCACAGCCCACAACGGA
WDR24	84219	14149986	GAACACAAGCGCACGGUAA
WDR24	84219	14149986	GGGACGUGCAGUUCAGUAU
WDR24	84219	14149986	CGACGUACCUGCCGACUAC
DTX4	23220	51468788	CCACAUCUACUGCUUGGUU
DTX4	23220	51468788	GGAUCGACCUCACUUCCAU
DTX4	23220	51468788	GGCUUUAGCUACGUAAUUG
DTX4	23220	51468788	GGUGAUGAGUGUUAAGGCA
TRIM26	7726	60279912	CAACGAGGCCUGAGGGAAU
TRIM26	7726	60279912	GCAUCGCCCUGGAUUAUGA
TRIM26	7726	60279912	UGAGAGACUUGGAAUAUAA
TRIM26	7726	60279912	CUAAACAGGUAUCCACGGA
PHF21B	112885	19923936	UGAGACUGAUACAGGGCGA
PHF21B	112885	19923936	GUAUUAAACUCGAUCGAUG
PHF21B	112885	19923936	GGACUCAGUUUGCCGUUGA
PHF21B	112885	19923936	UAGCUGAGUGGUAGCGGUA
MARCH4	57574	57977324	GUACUGCUAUGGAUUGUGU
MARCH4	57574	57977324	GACCGAGGAUCGCUACUCA
MARCH4	57574	57977324	GAGCUGGUCAUGAGAGUCA
MARCH4	57574	57977324	UGGCUCAUCUGGUCAACUU
ANKIB1	54467	51493000	GCACAUCCGUGGUAAGUUC
ANKIB1	54467	51493000	GCACAAUACUCCAUUACAU
ANKIB1	54467	51493000	GCCAGAAGAUUCAAUGUUU
ANKIB1	54467	51493000	UAUCAAAGAUGGGUCAGAA
OIT3	170392	22749296	CGGUAAAGCAGUACACGUC
OIT3	170392	22749296	CUACAGGGCCAGACGCUAA
OIT3	170392	22749296	UUGAAGAGCCUUACCGGGA
OIT3	170392	22749296	GGAAAUCAUGAGCCGAAAU
MGRN1	23295	44917607	GAGCACUCUUGUCCCUUUA
MGRN1	23295	44917607	GAACUCGGCCUAUCGCUAC
MGRN1	23295	44917607	GAACAUGGAUCUGAACUUC
MGRN1	23295	44917607	CGAAGGAGAUGUGGUGGAA
RNF7	9616	34304332	GCGACAAGAUGUUCUCCCU
RNF7	9616	34304332	CAAGAGGACUGUGUUGUGG
RNF7	9616	34304332	GCAUGUCCCUGUGGGUGAA
RNF7	9616	34304332	UGUCUUAGAUGUCAAGCUG
WDSUB1	151525	22749102	UCCAAUAACUAGAGAACUU
WDSUB1	151525	22749102	CAAUUCGCCUGUACUCGUU
WDSUB1	151525	22749102	GGAAUUACCUGCUGCGAUU
WDSUB1	151525	22749102	CCAGGUAUGUCACAACUUG
PCGF1	84759	14249239	ACAAGUAUGUCCGAUGUUC
PCGF1	84759	14249239	AGAGUUGUAUUGUGAAGUA
PCGF1	84759	14249239	CCACUACUAUCGCUAUGAU
PCGF1	84759	14249239	UGUCACCGCUUGAUGCUAA
TRIM3	10612	32454736	GCACAUAUGAGCUAGUGUA
TRIM3	10612	32454736	GAGCGCCACUGCACACGAA
TRIM3	10612	32454736	GUACAGCACAGGCGGCAAA
TRIM3	10612	32454736	GAAUGAAAUUGUAGUAACG
ZNF645	158506	22749188	AAAGUUCGUCCUCAUAUUG
ZNF645	158506	22749188	GAGAAGACAUAGACGGUAU
ZNF645	158506	22749188	GCCAACACCUCCCGACUAU
ZNF645	158506	22749188	UGACAAAGUCGGAUAUAAA
RNF5	6048	34305290	UCAAUGCCCAUGAGCCUUU
RNF5	6048	34305290	GCAAGAGUGUCCAGUAUGU
RNF5	6048	34305290	GCGCGACCUUCGAAUGUAA
RNF5	6048	34305290	GGCCAUGUCUUCAUCAGUG
PHF15	23338	40556369	GAAGAGAAGAGGCGAAAAU
PHF15	23338	40556369	GCUAUGACUUGGACGAGAU
PHF15	23338	40556369	GGGAGAGGGUGCAACGAGU
PHF15	23338	40556369	GGUGACAAGGCGCGAGAGA

BRCA1	672	63252875	CCAAAGCGAGCAAGAGAAU
BRCA1	672	63252875	UGAUAAAGCUCCAGCAGGA
BRCA1	672	63252875	CAACAUGCCCACAGAUCAA
BRCA1	672	63252875	GAAGGAGCUUUCAUCAUUC
ZFAND6	54469	21359917	CAAAGACAGAAUAGUAGUA
ZFAND6	54469	21359917	GUGUAUGUAUAUAUCGCUU
ZFAND6	54469	21359917	CAGAUUAUGUUCAUGGGUU
ZFAND6	54469	21359917	UAUGGAAACCCUCGUACAA
SH3RF2	153769	47578102	GCGGCCAGCUCCCUCAUUA
SH3RF2	153769	47578102	GGACAGGUCAGCACUUAUC
SH3RF2	153769	47578102	GCAAAGGCCUUAUGCAACU
SH3RF2	153769	47578102	GGGCGGAGCAGCAUGAGAA
RNF150	57484	58331203	UCGCAUGGCUCGUCUUUUA
RNF150	57484	58331203	GACGUCAUCUUUACUACUA
RNF150	57484	58331203	AAGCAGUGAUUCUGACAUU
RNF150	57484	58331203	CCAAUGACGUUGUCCGGAU
PCGF3	10336	59710102	GCACUUAGAUUCCCAUCGG
PCGF3	10336	59710102	GUACAUCGGUCAUGACAGA
PCGF3	10336	59710102	GGAGUUCUAUCACAAAUUG
PCGF3	10336	59710102	GCGACCGUCUUGCAUCUGA
RNF8	9025	34304335	GAGAUGGUCUGGAGGAUUC
RNF8	9025	34304335	AGAAUGAGCUCCAAUGUAU
RNF8	9025	34304335	GAGCGCGUCUGGAACCUUU
RNF8	9025	34304335	CAACAAGAGUCUAAAUGGU
RNF38	152006	37577182	GUAGUAUGCAUGUGUGAUU
RNF38	152006	37577182	AAUCGUACUUGCCCAAUUU
RNF38	152006	37577182	GCACUUACCAGUACCAUAU
RNF38	152006	37577182	GAGUCAAGGCAGCUACUUA
TRIM15	89870	16445349	GCGAGAACGAUGCCGAGUU
TRIM15	89870	16445349	GGGAGAAACUUACUGCGAG
TRIM15	89870	16445349	GCAGAACCACAGACGGCUU
TRIM15	89870	16445349	CCCUGAAGGUGGUCCAUGA
IRF2BP1	26145	24308114	CCUUCAAUAAACCGAAAGA
IRF2BP1	26145	24308114	CAAGAAGGAUCACGGGCUG
IRF2BP1	26145	24308114	CUUCAAGUACCUCGAAUAU
IRF2BP1	26145	24308114	CCUGAAGAAUGUGGCCGAA
MLL2	8085	4505196	GAUAUUAGCUACCACACAU
MLL2	8085	4505196	GCAAAUCGCUAGCAUCAUU
MLL2	8085	4505196	GCAUGAAGCCGCAGCAAUU
MLL2	8085	4505196	GAAAGGGCACUGAGGGAUA
PARC	23113	63082031	GGAUGACCCUGCCCACUGA
PARC	23113	63082031	GGUGUGGCCUGUACCAGUA
PARC	23113	63082031	GAACCAAGCAGCUCGUCUA
PARC	23113	63082031	ACGCACAGCUCUAUAGCAA
HR	55806	70906481	GGACAUACCAUGCCUGGCU
HR	55806	70906481	CCAAGAACCUCUGUGUGGA
HR	55806	70906481	CAUUACAGGAGGCCAAAUA
HR	55806	70906481	CCAGAGAACGGCAUCGUGA
PHF23	79142	13236519	GCAAGAAGCGGAAGUUAAA
PHF23	79142	13236519	AAGAAGAGAUGGCAACAGU
PHF23	79142	13236519	CGACAGUGCUACCUUGCUU
PHF23	79142	13236519	AAACGGCGGAGAACAAUUG
RNF25	64320	34878786	AGGCUGAGCGAAACCGAUA
RNF25	64320	34878786	GGUCAAAUCAGCAAAGGUU
RNF25	64320	34878786	GACCAGGAUUCACAGUAUG
RNF25	64320	34878786	UGAGUCAGCUGUAGAUGUC
PHF13	148479	24432092	GGGGAGAGGUGUUCGGUUU
PHF13	148479	24432092	CCGUGGUAGCUGUGCGUUU
PHF13	148479	24432092	ACUGAAGGCAAACGGACUA
PHF13	148479	24432092	CAAACAGCCAGGACGCAUU
DTX3L	151636	31377615	CCAAAGGAAUUGAGUAAGA
DTX3L	151636	31377615	GCACCAUUGUGAUUACUUA

DTX3L	151636	31377615	GGACACCAUUAGUAACAAA
DTX3L	151636	31377615	GAAUACAGCGAACUGCAUA
TNFAIP3	7128	26051241	UCUGGUAGAUGAUUACUUU
TNFAIP3	7128	26051241	CAACGAAUGCUUUCAGUUC
TNFAIP3	7128	26051241	CUGCAGUACUUGCUUCAAA
TNFAIP3	7128	26051241	CAACUCAUCUCAUCAAUGC
MKRN3	7681	74272285	GGGGAGAACUGUCGCUAUU
MKRN3	7681	74272285	GUAGAAAGCUGGGCGGAUG
MKRN3	7681	74272285	AACCAUUCCUUCUGUAUUA
MKRN3	7681	74272285	AUUAUGAGCUGGAAGAAUA
PHF12	57649	75677337	GAAAGACUGUCCAAUCACA
PHF12	57649	75677337	GCACUUAGCCACCCAAGCA
PHF12	57649	75677337	GGGAUUAGCACCCAGAAUU
PHF12	57649	75677337	CAACAGCCAACCAACGAUU
MID1	4281	15451851	GAACAAGUGUCUGACGAUU
MID1	4281	15451851	AGAAUGAUCAUGCGCGUUU
MID1	4281	15451851	UAACGGCUCUAUCGCCUUU
MID1	4281	15451851	CAUGAUAACUUGACAGUAG
TRIM14	9830	15208664	AACAGGGUCUGGAGUAUCA
TRIM14	9830	15208664	CAACAUAACCCAGAUAGAA
TRIM14	9830	15208664	GCCAAGAAAUUCAUUGAUA
TRIM14	9830	15208664	CAGAUUACUACUUGACGAA
RUFY1	80230	22095370	CAUCAGAUAUAGCGACUAG
RUFY1	80230	22095370	GAACUUAACCGGCACUUGA
RUFY1	80230	22095370	AUAAACAUCUCUUAAGCGA
RUFY1	80230	22095370	GAAAGAUGACGAAGCGACA
ZNF330	27309	13384595	CCUAAAUGUGGGCAUGAAA
ZNF330	27309	13384595	GAUCGAAGCAAUUGAAGUA
ZNF330	27309	13384595	GCAAUAGGGUGUAGCGUUU
ZNF330	27309	13384595	GCUGAGAACCGCCGAGAAC
RNF148	378925	37675276	GAUCACAUCGGAAUUAGGA
RNF148	378925	37675276	CUACUUAGCUUUCCUGACU
RNF148	378925	37675276	GAGGCGAAGUCAAAUAAAG
RNF148	378925	37675276	GCAUAGUUCUGUUUCAUCU
VPS41	27072	18105060	GAACGGUCUUGGAUGAACA
VPS41	27072	18105060	AGUCAUAGUUCAAGCAGUU
VPS41	27072	18105060	UGACAUAGCAGCACGCAAA
VPS41	27072	18105060	CAGCAUGUGUAUUUGCAUA
UBE4B	10277	38327033	GGACUUGAUUGGCCAGAUU
UBE4B	10277	38327033	GCAACUAGACACCGCGAAA
UBE4B	10277	38327033	CAAGAACGCACGCGCAGAA
UBE4B	10277	38327033	GCUAAACACUGGCUCCAAU
CCL20	6364	4759075	CAAUGAAGGCUGUGACAUC
CCL20	6364	4759075	GCUAUCAUCUUUCACACAA
CCL20	6364	4759075	CCGUAUUCUUCAUCCUAAA
CCL20	6364	4759075	UGAAAUAUAUUGUGCGUCU
RNF141	50862	38045936	UGUCAGAAGUGUAUUGAUA
RNF141	50862	38045936	UUAAUGAUGUAACGGCUAA
RNF141	50862	38045936	AAGAUGAUAUGGCUAACUA
RNF141	50862	38045936	GAUCAGACACAGUUGGUUA
WHSC1L1	54904	13699812	GCUUGAGGUUCAUACUAAA
WHSC1L1	54904	13699812	GUCCACUGGUGUUAAGUUU
WHSC1L1	54904	13699812	GGUUGACACUGUAUCAGAA
WHSC1L1	54904	13699812	GAACGUGCUCAGUGGGAUA
MARCH2	51257	53729333	CCAGAAAGUUCGCCUGAAG
MARCH2	51257	53729333	GGAGACACCAGUAUGAAUG
MARCH2	51257	53729333	UGGCUUUCCUCAUCUAACA
MARCH2	51257	53729333	GAUGGUCCUUUCUGCCGGA
NDP52	10241	33519473	GGACGUCACAUGUCAUUAU
NDP52	10241	33519473	GGAAGACAACCCGUGAGUA
NDP52	10241	33519473	GGAAACCCAUAUUCUGGUA
NDP52	10241	33519473	GGAUUGGAUUGGCAUCUUU
UBR2	23304	27597060	GCGUAGGUCUGUUCGAUAU

UBR2	23304	27597060	CAACAGAGAUUACGCUUAC
UBR2	23304	27597060	UCAGAGAUCAACUGUAUUA
UBR2	23304	27597060	GCUUAGUGAUUCCAAAUUA
TRIM2	23321	15011942	GGUCAACUAUGGCCUCAAA
TRIM2	23321	15011942	GCAAGAGUGUGCUGCUUAU
TRIM2	23321	15011942	GUAUAUGCCUGGAACGGUA
TRIM2	23321	15011942	CAACCAAUGUGUGCAGAUA
RNF103	7844	37595534	GUUCAUGUGCCAAUAAAUA
RNF103	7844	37595534	UUACCAAUGUGGCGAUUUA
RNF103	7844	37595534	GCCAUUGUGUGGUAUGAAA
RNF103	7844	37595534	GAGCUUGGUUCUAGUUAAU
TRIML1	339976	31560863	UCAGAGAGCCUGUGUGUAA
TRIML1	339976	31560863	CAGCAAAUCAGAAGCCUAA
TRIML1	339976	31560863	ACACAACUAUUAAGACGAU
TRIML1	339976	31560863	GGACCUGUUCUCACUAAUA
TOPORS	10210	40805103	ACAAAUGGGAGUAUACUUA
TOPORS	10210	40805103	UUACUUAGAUCGCUGCUUA
TOPORS	10210	40805103	ACGAAAUGCUUCUGUGUAU
TOPORS	10210	40805103	UCAUGGAUCUUUAGUGAAU
WHSC1	7468	6594682	GGAGCAGGGCCUUGUCGAA
WHSC1	7468	6594682	CCGGGUGUUUAAUGGAGAA
WHSC1	7468	6594682	GGUCCAAAGUGUCGGGUUA
WHSC1	7468	6594682	UGUCAGUGGAGGAGCGGAA
TRIM17	51127	68160934	GAGCGGAGAGAACGCAUUG
TRIM17	51127	68160934	GUGAAAGGAUAGACACAGA
TRIM17	51127	68160934	GCUAAGAGGCUUUCUAGAG
TRIM17	51127	68160934	GAGCAGAGCUUAGCCGAGU
TRIM33	51592	74027250	GAACAUAGGUAUCAGUUUU
TRIM33	51592	74027250	CCAGCAAGUUGGUGUAAUA
TRIM33	51592	74027250	GGACAAACCACAUUAGUAA
TRIM33	51592	74027250	GGAAAGCAGUUGCAUUGUA
RC3H2	54542	9256536	GGGAAAGGUUGGCGCUAAU
RC3H2	54542	9256536	CAGCUAAUCUCACGUAGUA
RC3H2	54542	9256536	CAGACAUCUUUGCGGGAAA
RC3H2	54542	9256536	CCCAAGGAAGUGCGACUAA
LOC643904	643904	89041178	CCGUGUGCUAUGAGAAGUU
LOC643904	643904	89041178	ACGUCACAUUCCUCAGCAA
LOC643904	643904	89041178	AGUCACAGAUCUUUGUCAU
LOC643904	643904	89041178	GGGUGCUGCUGGUGAGGAA
TRIM40	135644	33469990	CCACAGAAAUUAGAGGUUA
TRIM40	135644	33469990	GGAAAGGACGGCCAAGGAA
TRIM40	135644	33469990	GCUCAGAAGCCUGGUCAUU
TRIM40	135644	33469990	GGAGAAAGGAGUCAGUGAA
RFPL2	10739	5730010	GAGAAUCUGUUCACCGCAA
RFPL2	10739	5730010	GAGCGUAUCUGCUGAGGAG
RFPL2	10739	5730010	GAAGUGGGCGCAUCAGACA
RFPL2	10739	5730010	CUUCGUAGACCGCAAGUUA
PHF16	9767	23110985	AGGAACAGAUCUUCGGUUU
PHF16	9767	23110985	CUGCUGAGGUAUUCCGGAA
PHF16	9767	23110985	GGAUGGAACCGAUCACGAA
PHF16	9767	23110985	CAGUGACAGUUCAGACGAA
TRIM22	10346	15208661	GUAGAUGUGUCUGGAAAGA
TRIM22	10346	15208661	AGUGAAAGCUGGACAUUGA
TRIM22	10346	15208661	UAAACGAGGUGGUCAAGGA
TRIM22	10346	15208661	GUACGCACCUGCACAUUUA
RNF31	55072	38045939	GCCGAGAUGUGCUGCGAUU
RNF31	55072	38045939	GGCGUGGUGUCAAGUUUAA
RNF31	55072	38045939	GCAGAAUACUCAUCCAAGA
RNF31	55072	38045939	CCUAGAACCUGAUCUUGCA
ZNRF4	148066	34222361	CCGGAGCACUUAAUGCCUA
ZNRF4	148066	34222361	CAGCGAAGCGGGUGACCAU
ZNRF4	148066	34222361	GUAUACCACUGUCUCCUCA
ZNRF4	148066	34222361	GGUCGUAUCAGCCUUCUUU

RNF166	115992	31343485	GACACAAGUUCUCCUACGA
RNF166	115992	31343485	GUGAAGCACUGUGUGGAAA
RNF166	115992	31343485	GCAGCUCUCAUCCUACAAA
RNF166	115992	31343485	GGACUACAGUAUUGACGAG
LOC649055	649055	89057653	GUCGAAUAAUGAACGAUAU
LOC649055	649055	89057653	AGGAAUCUGUGAACCGACA
LOC649055	649055	89057653	GCAUGAACUCACUGCGAAA
LOC649055	649055	89057653	ACAAAGAGUAGGCGAUGAU
RNF168	165918	31377565	GACACUUUCUCCACAGAUA
RNF168	165918	31377565	CAAAGUAAGGCCUGGUAAA
RNF168	165918	31377565	GAAAUUCUCUCGUCAACGU
RNF168	165918	31377565	AGAAGAACAGGACAGGUUA
TRIM43	129868	20270352	GCACAAUGGUUAACUCUGA
TRIM43	129868	20270352	GCACAGAUGAUCAGGAAUG
TRIM43	129868	20270352	GGACCCAUAGGCAAACAAA
TRIM43	129868	20270352	CCAGAGAAGUUGGGUCAAA
RNF112	7732	23199981	AGAGGGAGUUCGAGGAGUA
RNF112	7732	23199981	AUACAGACCUGGACUAUCU
RNF112	7732	23199981	AAACAGAGCUUCAUGGGAA
RNF112	7732	23199981	GAACCAAGGCCAUGCAAGC
RAD18	56852	14550404	GCUCUCUGAUCGUGAUUUA
RAD18	56852	14550404	GAAAUGAGUGGUUCUACAU
RAD18	56852	14550404	GGGAGCAGGUUAAUGGAUA
RAD18	56852	14550404	CCAAGAAACAAGCGUAAUA
STUB1	10273	56181386	CGCUGGUGGCCGUGUAUUA
STUB1	10273	56181386	GUGGAGGACUACUGAGGUU
STUB1	10273	56181386	UGGAAGAGUGCCAGCGAAA
STUB1	10273	56181386	GAAGGAGGUUAUUGACGCA
MARCH6	10299	33589845	ACGGAAAUCUGGCAAACAA
MARCH6	10299	33589845	CUAAGGAAUUUGAAUGAUC
MARCH6	10299	33589845	CAUACAAUGUCAUGCUCUA
MARCH6	10299	33589845	UCAUAGAUCUCGUCGCUUA
BARD1	580	4557348	UGGUUUAGCCCUCGAAGUA
BARD1	580	4557348	GAGCACAUCUUCUGUAGUA
BARD1	580	4557348	UCAGAUAUGUUGUGAGUAA
BARD1	580	4557348	CGACAUACCUUCUGUUGAA
RCHY1	25898	58331198	GCACUCUGCUUUAGAUAUG
RCHY1	25898	58331198	GAAAUGUAACUUAUGCCUA
RCHY1	25898	58331198	GCACAUUGUUUGGAGAAUA
RCHY1	25898	58331198	CUAGAUCGCUUUAAAGUGA
TRIP	10293	40807468	GCUCCGACUUCUUCGAUCA
TRIP	10293	40807468	ACGCAAUGCUACUGUGGUA
TRIP	10293	40807468	GCAAAGAUGUGGUAAGGAC
TRIP	10293	40807468	GCUGAGGAAGGAUUUGUUU
LMO6	4007	7710128	CCUUUAGCCAGCAGCGGAA
LMO6	4007	7710128	GCACAGAGUUAGCGCCAGC
LMO6	4007	7710128	CACUAGGAGGGCAGCGCUA
LMO6	4007	7710128	CGCAUCAUGUGUCGGCUAA
TRIM52	84851	34147443	GGGACAACGUAGACUAUAU
TRIM52	84851	34147443	GCAAGGCCUAUAAUCAGUA
TRIM52	84851	34147443	GAAUUGAUGUCUACCGAGA
TRIM52	84851	34147443	GGUGGGAAUACUUCAGAUA
CBLC	23624	20149595	GGCCAACACUCCUCAAGAA
CBLC	23624	20149595	GCAACAAGGAUGUGAAGAU
CBLC	23624	20149595	GAACAGCAGUGACCAGGAA
CBLC	23624	20149595	CAUUUGAGCUCUGCAAGAU
RNF44	22838	42718018	ACAAGUGGUUGAAGGCCAA
RNF44	22838	42718018	GCACAUCCCUGGCUGCAGU
RNF44	22838	42718018	CCUUUGGUGUGCCAUAUUC
RNF44	22838	42718018	CAGCAGAGCCCGUUCAUGG
MLL3	58508	10864040	CCAGGUCAAUCAACAGUUA
MLL3	58508	10864040	CCAAAGCAUUUCAUCAGUA
MLL3	58508	10864040	GCAGUUACCAGAUACUUUA

MLL3	58508	10864040	GCAAUGGUCUUUCUGGAUA
RNF149	284996	31543079	GCUAGAAGCCGGCAGGAGU
RNF149	284996	31543079	GAGUCUAGCUUUACCAGAU
RNF149	284996	31543079	UAUACUGGCUCUCAGAUUG
RNF149	284996	31543079	CAUGAUGAUUAUCUCGUUA
TRIM37	4591	52487175	AGAGUGAGUUGAUAUCUAA
TRIM37	4591	52487175	GAAUGUAGAAGCUGUAAGA
TRIM37	4591	52487175	AUACGAAACUCCACAAAUA
TRIM37	4591	52487175	GGACUUUGCUGGAGGUUAA
BIRC7	79444	21536419	GGAGAGAGGUCCAGUCUGA
BIRC7	79444	21536419	GGAAGAACCGGAAGACGCA
BIRC7	79444	21536419	GCUCUGAGGAGUUGCGUCU
BIRC7	79444	21536419	GGAAGAGACUUUGUCCACA
CBLB	868	54112419	GGUCGAAUUUUGGGUAUUA
CBLB	868	54112419	UAUCAGCAUUUACGACUUA
CBLB	868	54112419	GUACUGGUCCGUUAGCAAA
CBLB	868	54112419	GAACAUCACAGGACUAUGA
RNF6	6049	34305296	GCGGUUAGAUGGCGUCAAG
RNF6	6049	34305296	GAGAGACCAUAAUCUUUUA
RNF6	6049	34305296	GAAUCAGUCUAGAUCGAGA
RNF6	6049	34305296	GGAACGAAUUACAGAGACU
RNF144	9781	38045937	GACCAGUGCUGCUUUCAAA
RNF144	9781	38045937	GUGCAAAGCCUGCCGUAUG
RNF144	9781	38045937	GAACGAGAUUGAGUGCAUG
RNF144	9781	38045937	GCAAUUAGCUGCCCAGAUG
LONRF1	91694	40217795	GGAAAGCGGUUUAGGGUUU
LONRF1	91694	40217795	GUUUAGAUCAUGCACCAUA
LONRF1	91694	40217795	GAUACCAGCUGUCGGUUUU
LONRF1	91694	40217795	GAAGGUCUGUGGUUGAUAC
TRIM55	84675	34878851	GAAAUGUGCCAGUGAUAUU
TRIM55	84675	34878851	GAGCAGCAGACCAUGGAUA
TRIM55	84675	34878851	UGGUUGAACUCCCUAAAUG
TRIM55	84675	34878851	GCGCAUCUCUGAAUUACAA
BRPF3	27154	55742814	AGGCAGAGAACAUCGGCUA
BRPF3	27154	55742814	GCAUCGAGGAAGAGCGCCA
BRPF3	27154	55742814	GUAUAAUGCUAAAGACACA
BRPF3	27154	55742814	UGAUUGAGCUGAUUCGGAA
RFFL	117584	62865648	CAUGACAUCUCUACCGAAA
RFFL	117584	62865648	GAGGAGAACCUGUGUAAGA
RFFL	117584	62865648	CCUGAGAGCUUGCAUCGGU
RFFL	117584	62865648	GGACUCACCCAUUGACUGU
LRSAM1	90678	53729362	CAGAUCAGGAGCCAGAUUA
LRSAM1	90678	53729362	GAACGAUUCCAGCAGAUUC
LRSAM1	90678	53729362	UGACGGAGUUAGAAGCCAA
LRSAM1	90678	53729362	GCAGAUGACAUUCUCGACA
RNF139	11236	38045935	GGGAGCCGCUUACAAGAAA
RNF139	11236	38045935	UGACAGGCGUCUUGGCUUU
RNF139	11236	38045935	AGAGAGACUUUACUGUUUA
RNF139	11236	38045935	GGGAAAAGCUUGACGAUUA
RAPSN	5913	38045927	GAGAAGCUGUGCGAGUUUC
RAPSN	5913	38045927	UGUCUGAGCGAGAGCAUUU
RAPSN	5913	38045927	GGGCGCACGUUGUGAGGUU
RAPSN	5913	38045927	CAUUGCAGGUGUGGACAAA
PJA1	64219	74048536	AAACUGCCCUUGCGCACUU
PJA1	64219	74048536	GACGAUAUGCCACACAGUA
PJA1	64219	74048536	CGACGAAGACUCUGACAGU
PJA1	64219	74048536	GGACGCACAGUGAUGAUUA
RKHD2	51320	39545576	GGAGUGAUCCUUCUGGUAA
RKHD2	51320	39545576	GAAACUAUAUAGAGCUCAA
RKHD2	51320	39545576	GCGCAAGAAUGAUAUCCAA
RKHD2	51320	39545576	CUAAUGGUACCAAUAGUUA
DPF1	8193	4758797	AGGAAAAGGCUUCUGCUUA
DPF1	8193	4758797	GGCCAAAGGAAAGGCAUAU

DPF1	8193	4758797	CAAGAUCGACUGUGAAGCA
DPF1	8193	4758797	CGGGACAGAUUUACACGUA
UBE4A	9354	38327028	GAUAAUAGCGUGUCAGAGA
UBE4A	9354	38327028	GCAGAUAACCUGGGUGAUU
UBE4A	9354	38327028	GCCAAUAGAGCUAACCUUU
UBE4A	9354	38327028	CCAGGCAACCACUUAAUUA
DTX3	196403	31341899	CGAAUGUCAUCACCUGGAA
DTX3	196403	31341899	GGAGUUCGGUAUCCUGGCA
DTX3	196403	31341899	GGCGGAUGCUGGUCUCUAA
DTX3	196403	31341899	CCUCAUAGAUGGCGAGACU
AMFR	267	21071002	GCAAGGAUCGAUUUGAAUA
AMFR	267	21071002	GGACGUAUGUCUAUUACAC
AMFR	267	21071002	GUAAAUACCGCUUGCUGUG
AMFR	267	21071002	GAAUUCGUCGGCACAAGAA
SCEL	8796	21536305	GCAGAUACAUCCACCUAAA
SCEL	8796	21536305	GGAAGUCCGAUUAAAGUUA
SCEL	8796	21536305	UCUCGAAUCUGUUGCUAAA
SCEL	8796	21536305	GAUAACCAACUAACCAAUA
MARCH5	54708	58532582	GAAUAAUGGUCGGCUCUAU
MARCH5	54708	58532582	GCGCAAAUACUCGAAUAAA
MARCH5	54708	58532582	GCUGAAUACCUAAUAGUUU
MARCH5	54708	58532582	GUAAAUUGAUGUUCAGUAG
LOC652591	652591	89071835	CUCCAAUGCAACCGGGAAU
LOC652591	652591	89071835	GGAACUACUUAACGGACAC
LOC652591	652591	89071835	AGGGAGCUCUGUUGCUUUA
LOC652591	652591	89071835	CUGUCCUGUGUGUGGGAAA
MAP3K1	4214	51464911	GUGGAGAUCUCAUGAUUUC
MAP3K1	4214	51464911	ACAUUUGUCUCCUGGUUUA
MAP3K1	4214	51464911	GACUAAGAAUUGCAGAUUU
MAP3K1	4214	51464911	GAUUAGAUGUCAAUACAGA
C1orf164	55182	46397374	CGGAAUGACAGAUGUAAGA
C1orf164	55182	46397374	GGACGUGCAUAUUCCUUUC
C1orf164	55182	46397374	CAACGGUUCCUAUACCUUU
C1orf164	55182	46397374	CCAAGCGACUUAAGAACUG
LOC120824	120824	89033943	UCAUAAGAUGGUUGCAUUU
LOC120824	120824	89033943	CUCAAGGGAUUCAGAGUUU
LOC120824	120824	89033943	GGAUAGAAGUUCCCUGAUA
LOC120824	120824	89033943	GCAAGAGCCAGUAGUCAUA
LOC441061	441061	88987268	GGAUAGACACACUGCCGCA
LOC441061	441061	88987268	GUGAUGGGUCAGUUCGGUA
LOC441061	441061	88987268	CAUUGACAGCUCUGCGGAA
LOC441061	441061	88987268	UCAGUGUGGCUAUGUGUUA
M96	22823	6678763	CUUAGCAGAUCAGGAGUUA
M96	22823	6678763	GAACUUGAGCUUAUGACAU
M96	22823	6678763	GUAAUUCACUGGUCCACAA
M96	22823	6678763	CCAAUGAAAUGGUUAUAUG
RNF40	9810	37588854	CUACGCAGAUGCCGACGAA
RNF40	9810	37588854	GAACUCCGAGAACGAAUUG
RNF40	9810	37588854	GAGAUGCGCCACCUGAUUA
RNF40	9810	37588854	GCUUAACUCUGGCUACUAU
RNF20	56254	34878776	CAGAAGAAGCUACAUGAUU
RNF20	56254	34878776	GUAUCAUCCUUAAACGUUA
RNF20	56254	34878776	UAUCAAUGCUCGGAAGUUU
RNF20	56254	34878776	GCUAAACAGUGGAGAUAAU
PHF14	9678	55769549	UCGUAAACUUAUGCGGAAA
PHF14	9678	55769549	CCAGUAACACUAACGGAAA
PHF14	9678	55769549	CCGAUAAGAAACACGAGAA
PHF14	9678	55769549	GGAGUGAUGAAGACGAGAA
LOC729974	729974	113428354	CAUUAGAUGUCCUGUCUGU
LOC729974	729974	113428354	GGUAUGAGGUCCAUUGCCU
LOC729974	729974	113428354	ACAUCAAGCCCAAGUACAA
LOC729974	729974	113428354	GGGAUGUGGGCGUGUGCAA

RNF157	114804	58743364	UGAGAAGCCUGGUCAAUAU
RNF157	114804	58743364	CCAGAGGACGUGCGCAUUU
RNF157	114804	58743364	AGAGGGAAAUGAUGUUAUA
RNF157	114804	58743364	GUACAACACACAAGAUUCU
LOC51255	51255	75709223	CCAUUGAGAUGCCUUGCCA
LOC51255	51255	75709223	GCAAGGUCACUUUUCAAUA
LOC51255	51255	75709223	CGUCCUAUUUCGAUGAACA
LOC51255	51255	75709223	UGGCUAAGCAAGACAAAUU
ZNF547	284306	38044285	GCAAAGCAGUGCAGUGAAU
ZNF547	284306	38044285	GGAAGACCGACAUUUGUGA
ZNF547	284306	38044285	CCACAAACAUAAACUUUCU
ZNF547	284306	38044285	GGAAAUUGUUUAUGUGGAG
SHPRH	257218	27436872	GAAAUCACAUUAUCAAGGU
SHPRH	257218	27436872	GCCAGAAGCUAGUAAGAGA
SHPRH	257218	27436872	GCACAAAUCAGUCGUGUUA
SHPRH	257218	27436872	AAAGAAAUCUCGUAUGUCU
KIAA1542	57661	55741656	GAGCUCAAUUUGGUGGUAA
KIAA1542	57661	55741656	GUGGAGAGCAUCUUUGGUA
KIAA1542	57661	55741656	GUUGAUCGAACUCUAUUUA
KIAA1542	57661	55741656	GGACACGUCUUUGAUGAUU
UHRF2	115426	23312363	CUACACAGAUUGAGGCUAA
UHRF2	115426	23312363	GCAGGUUACCCUUCAGAUA
UHRF2	115426	23312363	GAUCUUAGACCACGAGCUA
UHRF2	115426	23312363	GGAGCAGAGUCUCGGAAUU
TRIM35	23087	70608107	UUGAUAAGCUUCGCGAGUU
TRIM35	23087	70608107	UGUGGAAUCUGUACCCUUC
TRIM35	23087	70608107	AGGGCGAGCUGUCUUUCUA
TRIM35	23087	70608107	GAAGGAGGACGACGUUUCU
CNOT4	4850	56550058	CGUCUUUGUUGUAGGUUUA
CNOT4	4850	56550058	CCAAUUCUCUCAAUAGUAC
CNOT4	4850	56550058	UAACAGAGUCACAGUCGUU
CNOT4	4850	56550058	GGUAGUAGAUGGCAGAACA
RFPL3	10738	5730012	GGAAUUUUCUACUUGGUAA
RFPL3	10738	5730012	GAUAUGGGACAUUUCUAUA
RFPL3	10738	5730012	AUUCGAUUAUUGAGUCGUA
RFPL3	10738	5730012	GAAAUGUUCUGUAUUCUCG
CHD4	1108	51599155	GAGCGGCAGUUCUUUGUGA
CHD4	1108	51599155	GAAUAAAUUUCUAGCUCGA
CHD4	1108	51599155	GGUGUUAUGUCUUUGAUUC
CHD4	1108	51599155	AAGAAGAUCUAGCCCGAAA
RNF34	80196	37595536	UCUGAGAAAUAUACCCAUA
RNF34	80196	37595536	GGCCCAACAUAGUUUGUAA
RNF34	80196	37595536	GCUUAUGGAUGGAGACCAA
RNF34	80196	37595536	CGGCACAGGUACAAAGUGA
TRIM64	120146	89034199	GACAUCGAGAGGAGUGUAA
TRIM64	120146	89034199	UGUGGUUAUGAAUGGGAUA
TRIM64	120146	89034199	GGAUGAUUGUGGCAAGUUA
TRIM64	120146	89034199	UCAGUAAGGAAGAGGAUAA
ZNF185	7739	6005971	GGGCAGAAGUGUAGGGUUC
ZNF185	7739	6005971	CGAGAGAGUUGUGGCAGUA
ZNF185	7739	6005971	GUACGUGAAUGCUAGUGAA
ZNF185	7739	6005971	GCUGAUGACUCGUGGACAA
SMARCA3	6596	21071053	GCAGGAUCUUCUAAGGUUA
SMARCA3	6596	21071053	GAUAGAGAAUGGUGGCAUA
SMARCA3	6596	21071053	CCAGAUGACUUUCUAACUA
SMARCA3	6596	21071053	GGAUUUGUGUUUACUCGUU
DTX2	113878	24308252	GAUACAGCGAUGUGACUGA
DTX2	113878	24308252	GGACCAUCCUCAUAGUUUA
DTX2	113878	24308252	UGACCAACCUCCCUGCAUA
DTX2	113878	24308252	GUAUUACGGUUCCAGAUGU
MIB1	57534	62868229	CAGCAUACCCAGAUUGUUA
MIB1	57534	62868229	CGACAGACACCACUUCAUA
MIB1	57534	62868229	GGUAUGCUCUGACAAGAAA

MIB1	57534	62868229	GACCUGAGCAUUCGAAAUA
TRIM56	81844	30794215	GCGACUUCCUGGCCUGUAA
TRIM56	81844	30794215	GCAGAAUAGUGUGGUAAUC
TRIM56	81844	30794215	ACGUGGAGGUGUACAAUAU
TRIM56	81844	30794215	GCGCACGGCUCUAUCUCAU
RNF167	26001	14149701	GGAAACGGCUCCAGCGGAA
RNF167	26001	14149701	CCAAUGAACUUCUGAACAU
RNF167	26001	14149701	GUAAUGAUAGCUCGUUGUA
RNF167	26001	14149701	GGUCAAUGGGUCAGUCUUU
LONRF2	164832	38348241	AGACAAACUUUCGGAAUUA
LONRF2	164832	38348241	GAUGCAUGCUGGAGAUUAA
LONRF2	164832	38348241	CGACGGAUAUUAGUCAUCA
LONRF2	164832	38348241	GAUAAGAAGAUGCAUGGAA
LOC642446	642446	169203567	GGACUGCAGAUGCCAAUUU
LOC642446	642446	169203567	AAAGUUGGUUUCACGAUGA
LOC642446	642446	169203567	UAAGACACUAUGUGCGAGA
LOC642446	642446	169203567	GCAAAGGCCUCUGGGUCGA
RFWD3	55159	71143111	GGGCAUCUCUUUGGGUAUA
RFWD3	55159	71143111	GGACCUACUUGCAAACUAU
RFWD3	55159	71143111	GCAGUCAUGUGCAGGAGUU
RFWD3	55159	71143111	GUUAAGAUGUUGAGUACUG
LOC642219	642219	89035027	GCAAAUAUCACCUGCGUAU
LOC642219	642219	89035027	GCACAUUUCAUUCGUUAAA
LOC642219	642219	89035027	AAAGUUGGUUUCACGAUGA
LOC642219	642219	89035027	UAAGACACUAUGUGCGAGA
RNF151	146310	51473159	GACGCUGGCUGCAUAGUGA
RNF151	146310	51473159	GGGCCAACAUACCUUGUAA
RNF151	146310	51473159	GAAGUCAAGUGCAAGAACG
RNF151	146310	51473159	GGUAUGAUCUCAACCUCUU
WDR59	79726	58331265	GAGCGGAAAUCAAGACGAU
WDR59	79726	58331265	AAACGUGGUUGUAGAGUUC
WDR59	79726	58331265	CAUAUUAGAUGGUGUUGAU
WDR59	79726	58331265	GAGCUGAAGUGUUGAAGUU
VPS11	55823	62865896	UAUUUGAGAUGGCGAUUAA
VPS11	55823	62865896	GAACGUCCAGUCCUAUAUA
VPS11	55823	62865896	UUUAGAGGCUACCUUAUCA
VPS11	55823	62865896	ACUCGAAUCUUCCCUGCUA
ZNF278	23598	14670363	UGAGGUCGGUUAUCGAGAU
ZNF278	23598	14670363	GAACGGACAUAUCAAGCAG
ZNF278	23598	14670363	GGGUUGCGGUUCAAGAGAA
ZNF278	23598	14670363	GCAGGUGGCUUGUGAGAUC
BRAP	8315	10800416	GGAGAGAAAGUAGCGAUUA
BRAP	8315	10800416	CGACGUAAUUGAACAAAUG
BRAP	8315	10800416	GGUAUUAUGCACCUAUAUA
BRAP	8315	10800416	UGAGAAGUGUGAUAAUCUA
C13ORF7	79596	21362045	GAACGAAGUGAUAAGUAUA
C13ORF7	79596	21362045	CCAAAGGUUCUCUAACUAA
C13ORF7	79596	21362045	CGAAAUAGAUUGUUUACAG
C13ORF7	79596	21362045	GAUAACAGAUCACCUCAAA
TRIM73	375593	65285120	CAAGGAGUCCCUAAUGCUA
TRIM73	375593	65285120	GGAAGAUAGAGGAAAGUCA
TRIM73	375593	65285120	CUCCCAAGCAGCUGCCUAU
TRIM73	375593	65285120	CUACAGAAAUACUAGAGGA
IBRDC2	255488	50284695	GAAUAAACUUGGCCACUCA
IBRDC2	255488	50284695	GGUACUGCCUCCAGAACUU
IBRDC2	255488	50284695	GAGAUUGCCUGUUUGGUAC
IBRDC2	255488	50284695	CAGCUUGCCUGAAACAGUA
PPIL2	23759	22547209	GCAAGAAGCAUUAUUACGA
PPIL2	23759	22547209	GUACAUUACCUGUGCUGAA
PPIL2	23759	22547209	GUGCUGCGCUACCAGUUUG
PPIL2	23759	22547209	GGACCCGUCUUAUUAUCUG
ARIH2	10425	83776589	CAAUACACCUACCCAUAUG
ARIH2	10425	83776589	GCAAGUUUCAGAGAUAUUG

ARIH2	10425	83776589	GCAACGAGGUCUUCUGUUU
ARIH2	10425	83776589	GUAUGCAGUUUGUGCGAAA
TIF1	8805	47419908	GAGCAUAGAUACCAAUUUA
TIF1	8805	47419908	GAAGAACGCCAGUUGCUUA
TIF1	8805	47419908	UAACUGUGCCUGAUUAUUA
TIF1	8805	47419908	GAUCAUAGAUACACUAAUC
TRAF3	7187	22027615	UCAUUUACAGCGAGUGAUA
TRAF3	7187	22027615	AAGGAAAUGCUUCGAAAUA
TRAF3	7187	22027615	AAGAGAGCAUCGUUAAAGA
TRAF3	7187	22027615	CGCUAAAGCUGCACACUGA
TRIM4	89122	15011940	CCAAGUGGCUGUAAACCUA
TRIM4	89122	15011940	GGGAAUUACUGAUCGUUCA
TRIM4	89122	15011940	GCAUCUGGGCGAUUUAUUG
TRIM4	89122	15011940	GGACCUGUUUCUUCAGAGA
PCGF6	84108	37655164	GCAAAGGUUACUUAAUAGA
PCGF6	84108	37655164	GAGCAGUAUCAAACUCUAA
PCGF6	84108	37655164	GCAAUAUAGUAGUACAUCA
PCGF6	84108	37655164	GAGGAGGAGCGCCUGAUUA
TRIM31	11074	62865892	GGAGAAGAAUUUCCUGCUA
TRIM31	11074	62865892	GGAAGAACGCAAUCAGGUU
TRIM31	11074	62865892	CCACAAAUCCCAUAAUGUC
TRIM31	11074	62865892	AAUUUGAACUCCUGCAUCA
LOC652433	652433	89034678	UCAUUAAGGAAGGUGAUAA
LOC652433	652433	89034678	GCAGAUACCAAUAUAGUUA
LOC652433	652433	89034678	GGAAUGCAGGGUACAGAAA
LOC652433	652433	89034678	GUGGGAAAUAUAUCGGCAA
ZNF650	130507	40255162	AGAAAAGUCUUACGAAGUA
ZNF650	130507	40255162	AGGCAAACCUCUCUACAUU
ZNF650	130507	40255162	AGAUCGACCUACUGGAUUA
ZNF650	130507	40255162	GAGAAAGCUCACCCAGUUA
ISL1	3670	4504736	GCUCCAAGGUGUAUCACAU
ISL1	3670	4504736	GGACCGGGCUCUAAUUCCA
ISL1	3670	4504736	UGAAAUGUGCGGAGUGUAA
ISL1	3670	4504736	GCAGUGAAGUAGCAUCAAU
VPS18	57617	42544225	GACGUAAGGAUGACGCAAA
VPS18	57617	42544225	GGGCGUAGCUUUGUUAUUG
VPS18	57617	42544225	CAAAGCAGCGCAUUGACUU
VPS18	57617	42544225	GCAAUCAGCUGUGCAUGAG
PDZRN4	29951	39653318	GCGAACACCUCUUAGUAGA
PDZRN4	29951	39653318	GGGAAGAUGUCCAGAAUCG
PDZRN4	29951	39653318	GAAAUCUGCAGUCGAGUAU
PDZRN4	29951	39653318	GAUGAAAGCUUGCGAAAUG
RNF215	200312	63025219	CAGUGAUCGUCCUCCAUUA
RNF215	200312	63025219	CAGUGGCAUACGUGGGCAA
RNF215	200312	63025219	GGUUGAGGGAAGAGCUAUC
RNF215	200312	63025219	GAGUAGGGAAGGUUUAAUA
TRIM69	140691	88999602	GUACAUGGUAUGGAGGGAA
TRIM69	140691	88999602	AGGAUAUGUUGGUGAGCAU
TRIM69	140691	88999602	CGUCUGGCAUGGUGACAUU
TRIM69	140691	88999602	UGACUAACAACCUCGACAA
AIRE	326	4557294	GAAGAAUGAGGACGAGUGU
AIRE	326	4557294	CAACAGUCCAGGAGGUGCA
AIRE	326	4557294	GUGCGGAGAUGGUACGGAC
AIRE	326	4557294	GUGCUGCGGUGUACUCACU
PARK2	5071	7669539	UUAAAGAGCUCCAUCACUU
PARK2	5071	7669539	GAUAGUGUUUGUCAGGUUC
PARK2	5071	7669539	GAACAUCACUUGCAUUACG
PARK2	5071	7669539	GUAAAGAAGCGUACCAUGA
LMTK3	114783	51474756	CACAGUCGCUGCCUGAUGU
LMTK3	114783	51474756	CUACUGGUAUGACAUUCUU
LMTK3	114783	51474756	UCGAGUGGGCGGAGGAUUU
LMTK3	114783	51474756	GGGAGUGGCUGGUUUGGGA
LOC390231	390231	89035024	CACCUUGGAUCGUAAGAUA

LOC390231	390231	89035024	AAAUGUACUUCAAACGCAU
LOC390231	390231	89035024	UUUAUAAAUUUGCGGAGCA
LOC390231	390231	89035024	AGGAUUUGGGAGACGUAAU
LOC283116	283116	169202725	CCAUCAGGCUUCAUUGUAU
LOC283116	283116	169202725	UGAACGAAAUGUGCCAUAA
LOC283116	283116	169202725	AAUCUAUCCUUUGAGGUAA
LOC283116	283116	169202725	GCAUAUUACUCUGCAUCAU
TRAF6	7189	22027628	GCGCUUGCACCUUCAGUUA
TRAF6	7189	22027628	GGCCAUAGGUUCUGCAAAG
TRAF6	7189	22027628	GAUAUGAUGUAGAGUUUGA
TRAF6	7189	22027628	GGAGACAGGUUUCUUGUGA
RNF2	6045	54792140	CGAGAUACAUAAAGACUUC
RNF2	6045	54792140	GUAUCUGGCUGUGAGGUUA
RNF2	6045	54792140	GGCAAUUGAUCCAGUAAUG
RNF2	6045	54792140	ACAAAGGAGUGUUUACAUC
PXMP3	5828	4506342	AUUUAAACCUGGGCUGUUA
PXMP3	5828	4506342	CAGAACGUCUCCUAGGUAU
PXMP3	5828	4506342	GAUAUCAGCCACCCAGUAA
PXMP3	5828	4506342	GAAGAACGAUGCUAUGAUU
TRIM25	7706	68160936	CGGAACAGUUAGUGGAUUU
TRIM25	7706	68160936	CAACAAGAAUACACGGAAA
TRIM25	7706	68160936	GCGGAUGACUGCAAACAGA
TRIM25	7706	68160936	GGGAUGAGUUCGAGUUUCU
BIRC2	329	41349435	UAUAGGACCUGGAGAUAGG
BIRC2	329	41349435	GAAAUGCUGCGGCCAACAU
BIRC2	329	41349435	GAAUGAAAGGCCAAGAGUU
BIRC2	329	41349435	UCGCAAUGAUGAUGUCAAA
BAZ2B	29994	7304922	AGACAAUGUUUCCGAGAUU
BAZ2B	29994	7304922	CAGGAUGAGACGUCGGAAA
BAZ2B	29994	7304922	CCAAGUAACUCGAGAUAUU
BAZ2B	29994	7304922	CGACAAGAACAUUGAUUAU
RNF130	55819	38176162	GUGCCGAAUUUGCCAUGUA
RNF130	55819	38176162	CAAAUGCACGCGACAGGAA
RNF130	55819	38176162	UAACGUAGCAUUCGAUAUG
RNF130	55819	38176162	GUAGAAUGGUUUUGAAGAA
TRAF5	7188	77404347	GAUCAAAGAUUAGCCGUUU
TRAF5	7188	77404347	GGUCACACCUGUCCCUAUA
TRAF5	7188	77404347	GAGCAAGACUGUCCUUUUA
TRAF5	7188	77404347	GCCAAGAACGCCUACAUUA
PRICKLE2	166336	38524619	GCGAUGAGCUGCUGCACAA
PRICKLE2	166336	38524619	CAAAGACUCUGGAAUGAAU
PRICKLE2	166336	38524619	CAGGAGAGCUACAGUGAUA
PRICKLE2	166336	38524619	GGAAGAGCCCUACCAUUAU
LOC653978	653978	89065688	UGUACAGGCUCAACCGCUU
LOC653978	653978	89065688	CAUGCAAUGCCUUCAAUUA
LOC653978	653978	89065688	CCAGAGAAGUUGGGUCAAA
LOC653978	653978	89065688	CGGUUCUCCAUAAGGAAGA
TRIM71	131405	84993741	CACCAAGGCCACAGGCGAU
TRIM71	131405	84993741	CUUGGGAUGUGGCGGUGAA
TRIM71	131405	84993741	AGAAAGUAGUGCUAGCCGA
TRIM71	131405	84993741	GGAGGAGGGUAGAGCGCUA
SH3MD4	344558	88953463	CCGGAAUAAUGUAGUCGGA
SH3MD4	344558	88953463	CCGCAAACCUCAACGGGGA
SH3MD4	344558	88953463	GAUGCAAACUAGACGAGAA
SH3MD4	344558	88953463	ACGUGUACCUGGCGCUCUA
TTC3	7267	49640010	CAAAGGGUCAUUAUCGUUA
TTC3	7267	49640010	UCAAAACAGUUCCGUAAUA
TTC3	7267	49640010	GACCAUAGCAAUCGAAAUU
TTC3	7267	49640010	UAACUUGGCCCACGAGUAA
LONRF3	79836	73747832	CCGGAAAUGUCAUGGGUUU
LONRF3	79836	73747832	UGAAUGCGCUCUAUGUAUG
LONRF3	79836	73747832	AAAGAUGCCUAGAUCACAA
LONRF3	79836	73747832	GUGAGUGGAUUGCCGAAGA

DZIP3	9666	40254857	GAUACAAGGUACCCAUGAA
DZIP3	9666	40254857	CCUAGAAUCACUUCAAUUA
DZIP3	9666	40254857	GGGAUAUCCUCCUUAGUGA
DZIP3	9666	40254857	GGGCUCAGCUGGCAAAGUA
TNFRSF25	8718	23200030	GAGAAGAUCUGCACCGUCC
TNFRSF25	8718	23200030	GGAAGGAGUUCGUGCGCAC
TNFRSF25	8718	23200030	GAUCGAAGCCGUGGAGGUG
TNFRSF25	8718	23200030	GCCGGUGACUUCCACAAGA
SPRYD5	84767	14249255	GAAAGAGCCAAUAGUCAUA
SPRYD5	84767	14249255	GAGCAACAUCACUUGGAAA
SPRYD5	84767	14249255	UACCAGCACAGUAGGAUUA
SPRYD5	84767	14249255	GGACAGCCUCAGUGGAUUC
UBR1	197131	83656781	GGAAAUCAGCGCGGAGUUA
UBR1	197131	83656781	GAUCAGCAAACCCACAAUA
UBR1	197131	83656781	GUACAAUCGUGUGGACAUA
UBR1	197131	83656781	GCGAAGAAAUGGACUGUCU
MLL4	9757	7662045	GGGCGAGGCCUGUUCUGUA
MLL4	9757	7662045	GAAUCAAAGUGCCCAACUA
MLL4	9757	7662045	AGACAUCUCUCCUUUAGUG
MLL4	9757	7662045	GCUAUGAAGACAACGACUA
RNF113B	140432	37059780	CGACCAAGCAGACCAGGUA
RNF113B	140432	37059780	GGAAUCCACAGCUACCUGA
RNF113B	140432	37059780	GAGGGUCGCUACUGUAUCU
RNF113B	140432	37059780	CCAAAGAACUGAUGGCGAA
PHF1	5252	21536450	CACAAGGACCGUUUCAUUU
PHF1	5252	21536450	CUGAUGGGCUGCUAUACUU
PHF1	5252	21536450	GAGGAUGAUUCGCAGUUUC
PHF1	5252	21536450	GAAGCUUUCUCUGCCAUAU
SH3MD2	57630	51988886	GAUCGUAGGUUCUCGAAAU
SH3MD2	57630	51988886	GGAUGAAUCAGCCUUGUUG
SH3MD2	57630	51988886	AAAGGCACAUUACAACGUA
SH3MD2	57630	51988886	AAAGGGACAUCCAUGCAUA
RING1	6015	51479191	CUGGAGGGCGUCAGUGAAA
RING1	6015	51479191	GAACUGAGUCUGUAUGAGC
RING1	6015	51479191	UCUCUAAGAUCUAUCCUAG
RING1	6015	51479191	CGAGGUAUGUGAAGACAAC
FSD1L	83856	89886461	GCAGGAACAAGCUCGUAAA
FSD1L	83856	89886461	CAUUAGAUAUAAAGGAACC
FSD1L	83856	89886461	GGAAAAUUCGUCCAACAUA
FSD1L	83856	89886461	AGUCAGAUUAGUCAAUGUA
RAG1	5896	4557840	GAAAUGAGUCUGGUAACAA
RAG1	5896	4557840	GCAAUGCAGCUGAGUUCUA
RAG1	5896	4557840	CAGCUAAACCUUUCAUUGA
RAG1	5896	4557840	GGAUGCAGUUUGUGAGUUA
TRAF4	9618	22027623	UGAUCUACCUGCACACUUG
TRAF4	9618	22027623	GAAACUAUGUGCGGGAUGA
TRAF4	9618	22027623	GGAGCUGGAAGUACAAGUA
TRAF4	9618	22027623	GUCAUUCCCUGCCCUAAUC
CGRRF1	10668	50726998	GUACCCAGAUCUCGCUAUC
CGRRF1	10668	50726998	GUAUUGGGAUGGUUUGGUU
CGRRF1	10668	50726998	GAGGAUGACCGGGAAAUUU
CGRRF1	10668	50726998	ACAGAUUGCCUUGAAGAUA
MSL2L1	55167	38570120	CCAGUACACAUGAUGAUAA
MSL2L1	55167	38570120	CUGACUAGGUGUAAUGAAA
MSL2L1	55167	38570120	CGACCAAACUUUCCUAAUA
MSL2L1	55167	38570120	GCAUAUAGAUCACUUGUAU
RNF138	51444	38045933	AGCCAUACAUCUUAAUGAA
RNF138	51444	38045933	GGGAAUAGGGAUAGACUUU
RNF138	51444	38045933	AAAGAGUGGUGUUUACUAU
RNF138	51444	38045933	GGAGGGAAUUGUAUUGAUA
TRIM23	373	44955904	GUAGAGUUGUCUUGGCAAA
TRIM23	373	44955904	CCCAUUUGAUCGACAAGUA

TRIM23	373	44955904	GUGCUAGAGUGUGGAGUUU
TRIM23	373	44955904	GAAACUCUGUGUCGUCAAG
RNF10	9921	34452680	GAUCCAAGCGUUAUAAUCG
RNF10	9921	34452680	GCGCAUUUGUAGCCAUGAA
RNF10	9921	34452680	CCACAGAGUCACAUCAGUA
RNF10	9921	34452680	GUAAAUGUCCCAUCUGUUA
DPF3	8110	13442997	AGGAUAUUCCCAAGCGAAA
DPF3	8110	13442997	GGGAAGCAACAGUGGCGUA
DPF3	8110	13442997	GGGCAUCGACUGUGUGCGA
DPF3	8110	13442997	CGGGCCAGCUGUAUACAUA
RC3H1	149041	73695472	UGCAAGAUGUGCCUGAAUA
RC3H1	149041	73695472	ACCAACAGAUCGAGAGUUA
RC3H1	149041	73695472	GCGAUUAGAUCUUCAGAGA
RC3H1	149041	73695472	GAAGAGAUAUUCCGAGAAA
RNF128	79589	37588870	GGUCAUAGAAGUAGGGAAA
RNF128	79589	37588870	GUCAACAAAUGAAAGUCUA
RNF128	79589	37588870	GCUCGAAGGCUACGGAAUG
RNF128	79589	37588870	AGACGAAACUCCUAAUCAA
NSD1	64324	27477094	GCCGAGAGCUGUUGAGAAA
NSD1	64324	27477094	GAUCAAAGCCUUCAUCCAA
NSD1	64324	27477094	GAACAGAAGUAGUACCAAU
NSD1	64324	27477094	GGACGAGAAUUCUUUGAUU
RNF41	10193	37588860	GACGUCAGCUGAACACAAA
RNF41	10193	37588860	GAAGAGAUAUAAGAGAACU
RNF41	10193	37588860	GGAGACAAUUGAAUACAAC
RNF41	10193	37588860	GAUGAGCUGCCCAACCAUA
RNF190	162333	22749228	UCAUUUAGGUUCCGAGAUG
RNF190	162333	22749228	CCAAACAGAGUUCUAGUGA
RNF190	162333	22749228	GAGCAGAGGUUUGCAGAAC
RNF190	162333	22749228	GAGAGAGGUUGUCAAGAAA
CXXC1	30827	52856410	AGAAGAAGGAGGAGCGAUA
CXXC1	30827	52856410	GAGAUUCGCUAUCGGCACA
CXXC1	30827	52856410	GCAGCAGAUCAAACGGUCA
CXXC1	30827	52856410	UCACAGAAGUUAGGGCGCA
LOC196346	196346	89033947	GCAUGAAGAUCUGGAGCCA
LOC196346	196346	89033947	UGGAUAAGGAAGAAUAGUA
LOC196346	196346	89033947	GCCUGAUAGUCUUUGCAUA
LOC196346	196346	89033947	CCGACUGGCUCCUUAAAUU
TRIM28	10155	14971416	GAAAUGUGAGCGUGUACUG
TRIM28	10155	14971416	GCGAUCUGGUUAUGUGCAA
TRIM28	10155	14971416	AGACAGCACUGGCGUGGUG
TRIM28	10155	14971416	GAACGAGGCCUUCGGUGAC
HBXAP	51773	38788332	GCUGGUAAAUGGAGAAGUU
HBXAP	51773	38788332	AGGCAAAGUUCGAUGGACU
HBXAP	51773	38788332	CUGUGUGACUCUUGCGAUA
HBXAP	51773	38788332	GAAGAAAGAGCGUGCCGAA
TRIM6	117854	51477693	GUGAUUGGGUUACAGCAUA
TRIM6	117854	51477693	CCACUACUCUUUGUCCAUA
TRIM6	117854	51477693	GCAACUGUGUAAUUCCUAU
TRIM6	117854	51477693	GGACCUACAUUCUCUUUCA
ANAPC11	51529	50409795	GUCAAACCCUGAGUCAUGA
ANAPC11	51529	50409795	UCUGCAGGAUGGCAUUUAA
ANAPC11	51529	50409795	AAGAUUAAGUGCUGGAACG
ANAPC11	51529	50409795	GGCCAACGAUGAGAACUGU
MARCH9	92979	53759067	GAGGGUCACUACAGUCUGA
MARCH9	92979	53759067	GCGCUGCGGUUAUACAAUC
MARCH9	92979	53759067	GACCAAGGACAUAGGAGGA
MARCH9	92979	53759067	GGAAGGUCCUAAAUUAUGA
MKRN2	23609	32880198	UCACAUGACUUGGCAAACA
MKRN2	23609	32880198	ACUGUGAGGUUCUUUAAUU
MKRN2	23609	32880198	GGAAUCAUCAGAACCCUAA
MKRN2	23609	32880198	GGACAAAGUGUGCAGUAUC
LOC51136	51136	21361528	GAUAAACUCCAUACCUAAA

LOC51136	51136	21361528	AAUCUAAGGGUUACUGGUA
LOC51136	51136	21361528	GCGUCAAACUUGUUAUGCA
LOC51136	51136	21361528	AAUGCAAGCUCCAGAAAUA
TRIM72	493829	56605980	CAUGCAUGCGUAAGGAGAA
TRIM72	493829	56605980	GAGCAGACAUUGCGGCACU
TRIM72	493829	56605980	GGGAGGUGGAUGUUGGCGA
TRIM72	493829	56605980	CCAGAAUACUGACAAGCGU
TRIAD3	54476	46370053	GUGCGCAGAUGCUCACUUG
TRIAD3	54476	46370053	UACCGUACCUCUAUUGAAG
TRIAD3	54476	46370053	GAAAAUAAGCGACGACAUU
TRIAD3	54476	46370053	GAAAACAAAUGAACCAGUA
KIAA0644	9865	76880479	CCGCCAAGGAACUCGUUUA
KIAA0644	9865	76880479	GGCUGGAUCUAGACGGCAA
KIAA0644	9865	76880479	AGGCGGACCUCAUCGAAUU
KIAA0644	9865	76880479	CGUAGGAAACUGCGGGCUA
LOC652859	652859	89065050	AGGAACUUAUAGUGAACAA
LOC652859	652859	89065050	GAAGGUUAGUACCGUAUUA
LOC652859	652859	89065050	AUGGGUGACUCUUAAAACA
LOC652859	652859	89065050	AAUGCAAGAAGACAAUACA
ZNF294	26046	31657110	GAUUGAAGGCUGGGAAUUA
ZNF294	26046	31657110	GCGAAAGGAUGCUUGCUAA
ZNF294	26046	31657110	AAACAACAAUGCUUCCAUA
ZNF294	26046	31657110	GGGUGUAUCUAACCUAUUA
FLJ31951	153830	21389514	UGACAAGUAUUGCGGAAUG
FLJ31951	153830	21389514	CAGAAUUGCUAGUAGUUUA
FLJ31951	153830	21389514	UGGUUCAGCUUUAUCUAUA
FLJ31951	153830	21389514	GAGAAUUGGUUCAGGUAGU
SYTL3	94120	58000458	GAUCCCAAAUGCUCUACUA
SYTL3	94120	58000458	UCAAGGAGUUAGAACGCGA
SYTL3	94120	58000458	GGUGGAAAGGAGCGAAGAA
SYTL3	94120	58000458	AGAAAUACGAAGACAGCGU
JARID1D	8284	56243542	ACAAGGAGAUGUUCAUUAU
JARID1D	8284	56243542	CAAAAUAGCUUACAACACA
JARID1D	8284	56243542	GUAGAAACGUUGAGAAUCU
JARID1D	8284	56243542	GGUUAUGGGUGCCAAGAUA
LOC653192	653192	169163293	UGUACAGGCUCAACCGCUU
LOC653192	653192	169163293	CUGUAACAACUCCUGGAUA
LOC653192	653192	169163293	CCAGAGAAGUUGGGUCAAA
LOC653192	653192	169163293	CCACAGAUGAUCAGGAUUA
TRIM46	80128	47174858	UGACAAAGAGCCUGACAUA
TRIM46	80128	47174858	GCGAAUACAGUGAAGAUGU
TRIM46	80128	47174858	GCCAACGCCUGGUAUGUCA
TRIM46	80128	47174858	GCUCAGGUCUGGUGGGCUA
PHF3	23469	7662017	CGAUAAGGAUCCUAUGCUA
PHF3	23469	7662017	CCACAGAUGCUCCGAAGAA
PHF3	23469	7662017	CAGCCGAAAUAGCGGACAA
PHF3	23469	7662017	GUAAGAAGUUUGCGACAGA
RFPL1	5988	10440557	GAGGAGCCACUGCACUUGU
RFPL1	5988	10440557	UAGGAACGCUCUACUCGGU
RFPL1	5988	10440557	UUGAGUAUCUGUCCUGUGA
RFPL1	5988	10440557	UGGAUGCGCUGUCUGCUUC
RNF183	138065	34147709	UCAACAACACGUUCCAUAC
RNF183	138065	34147709	GAGGGAGUGUUUCCGCAAC
RNF183	138065	34147709	GGUGAGUGCUGUUCCCAGA
RNF183	138065	34147709	GCUCAUAUUCUCCAUCUUU
ZSWIM2	151112	71043931	GUAAUAACCACAACCUAAA
ZSWIM2	151112	71043931	GGGAAGUGUUAUAAGUGUA
ZSWIM2	151112	71043931	CCGAAUGCAUAGAAUAUCA
ZSWIM2	151112	71043931	GAUCUUAGCUAAUUAUCAG
ZNF216	7763	5174754	CGAAACUGGUUAUAAGUAA
ZNF216	7763	5174754	GUGGCUGCCUUGCCUGUAA
ZNF216	7763	5174754	GCACAGAACUGCACUAGGA
ZNF216	7763	5174754	GCAAAUGACAGAAAUGAGC

PHF19	26147	58331162	GAUCCAGGGACUCGGGACU
PHF19	26147	58331162	GGAAGUGUGGCCUGGGUUA
PHF19	26147	58331162	CCUCGUGACUUUCGAAGAU
PHF19	26147	58331162	GGGAAGAUCAAGAGGGUCA
PDC	5132	32967589	GAUGAAAACUGCCUUCGUA
PDC	5132	32967589	GCAGAAUACCCUAUAGUUA
PDC	5132	32967589	GGUAUUAAGGGUUGUGAUG
PDC	5132	32967589	GACAGUGUAUGCAGGAUAU
DTX1	1840	41352717	AGACUCAUCUUCACUAUCG
DTX1	1840	41352717	GUACUCCAAUGGCAACAAG
DTX1	1840	41352717	GCAUGGACGGCCUACGAUA
DTX1	1840	41352717	GAAGUUCACCGCAAGAGGA
PHF10	55274	19747274	GCGCAGUGAUGAAGUGAUU
PHF10	55274	19747274	ACUUAAACCGGGAACGCAU
PHF10	55274	19747274	AAAGAACGUCAACGAAUUA
PHF10	55274	19747274	AAUAAAGGCACUUCGGACA
TRIM45	80263	50878292	GCACCGAGGAGUCUACUUA
TRIM45	80263	50878292	GGACAUACUACAUUUCCUA
TRIM45	80263	50878292	GCUCAGAUCCACAUAAUAA
TRIM45	80263	50878292	GUGCAGGGCUCGCCAUUCA
TRIM32	22954	15208649	UGGUAACUAUCGUAUACAA
TRIM32	22954	15208649	CCAAAUAGGACACACGAUG
TRIM32	22954	15208649	GGACAGUUAACGUGGAAGA
TRIM32	22954	15208649	GUGAAGUACUAGUCGCUGA
RNF121	55298	37588864	GAAAUGGUCCUCAUCCUCA
RNF121	55298	37588864	GCAAACGUGUCCCUACUGC
RNF121	55298	37588864	CGUAUAGGCUGUCCUGCAA
RNF121	55298	37588864	CCAUAGGGUUCUACAGCGA
RNF26	79102	34878803	UGUGUACUCUGCUGUAUAG
RNF26	79102	34878803	GCCGAGAGAGGCUCAAUGA
RNF26	79102	34878803	GCACUGAAAUCCUGAUGCG
RNF26	79102	34878803	CGUAGUGGCUGCCUUCCUA
KRTAP5-9	3846	40254818	CCAACAAGUGACUACCCUU
KRTAP5-9	3846	40254818	GCUCUAGUGGGAAACCUCA
KRTAP5-9	3846	40254818	CCCAUAUGUCUGAGCCAAA
KRTAP5-9	3846	40254818	CCUAUAAGAAUAUCCCAAG
RNF13	11342	34577092	UUACUGAGACCUUUAGCUU
RNF13	11342	34577092	GAAUCCCGCUCACAUCAGA
RNF13	11342	34577092	UGAACGGGAUUACAACAUA
RNF13	11342	34577092	GACAGAAUCUUCAGACUAU
IBRDC3	127544	23503262	GCCCGAAGCUAACUUGUGA
IBRDC3	127544	23503262	CAGCGAAACGGCCAGCUUU
IBRDC3	127544	23503262	CAGAUGGACUUAGUGUUAU
IBRDC3	127544	23503262	CCAAAUCAGACAUGCGAUA
VPS8	23355	57863274	GCACUUUGGUUUCCGUUAU
VPS8	23355	57863274	GCAAAUAUCUCCUUGUAUC
VPS8	23355	57863274	CAACCCAACCCAAGUUAUA
VPS8	23355	57863274	GCAAUAAGCUCCUUGUAUA
TRIM49	57093	35493773	CAAGAUGUACCCUAUUUCA
TRIM49	57093	35493773	CAUAUUACUCUGCAUCAUG
TRIM49	57093	35493773	GUUAAUCAAAGCUCCCUAA
TRIM49	57093	35493773	GAAGAUAGAUGGAAAGGCG
SYTL4	94121	18152766	GAACAAGGCCAGUAAACGU
SYTL4	94121	18152766	UGUUGGAGGUGACCGGAAA
SYTL4	94121	18152766	GCGACUAAAGAAUGAGUUA
SYTL4	94121	18152766	GAGAUGAAGAGGUCCGGAA
RNF4	6047	34305289	GAAUGGACGUCUCAUCGUU
RNF4	6047	34305289	GCAAUAAAUUCUAGACAAG
RNF4	6047	34305289	GACAGAGACGUAUAUGUGA
RNF4	6047	34305289	GCUAAUACUUGCCCAACUU
LMO7	4008	33598967	GAUGAUUACUCCACAAAUA
LMO7	4008	33598967	AGAGAGAGAAUUCCAAGUA
LMO7	4008	33598967	UAGCAGGAUUGGAUAAUAU

LMO7	4008	33598967	GAAAGCUUGUGAACAGAUU
CHFR	55743	8922674	CAACGUGGCAUACCUCUAU
CHFR	55743	8922674	CAACAACAGCUACGAGUCA
CHFR	55743	8922674	GCACUCAGGUGAAAGCUCA
CHFR	55743	8922674	GAACAGUGAUUAACAAGCU
MNAT1	4331	49574512	UAGAUGAGCUGGAGAGUUC
MNAT1	4331	49574512	CAGCCCAGUUAACCAUUUA
MNAT1	4331	49574512	GGCUAUACUUCUUCUCUUG
MNAT1	4331	49574512	GUAUUUAAACCAUGUCAGA
LOC646754	646754	169202589	GGAUAAUGCUCUGAGUACA
LOC646754	646754	169202589	UCAUUAAGGAAGGUGAUAA
LOC646754	646754	169202589	GUGGGAAAUAUAUCGGCAA
LOC646754	646754	169202589	GCAGAUACCAAUAUAGUUA
PCGF2	7703	37595566	GUGACGUGCAGGUCCAUAA
PCGF2	7703	37595566	ACGAGCCACUGAAGGAAUA
PCGF2	7703	37595566	GGAUUUCUAUGCAGCGUAC
PCGF2	7703	37595566	CAAAUUGGUCCCUGGGCUU
C1orf166	79594	13375704	GUACAACAGCUAAUAGUUU
C1orf166	79594	13375704	UAACAGCCAGUUUGUGGAA
C1orf166	79594	13375704	GGCAUGCAGUACUAUCUAA
C1orf166	79594	13375704	GGAGCUGUGCGGUCUGUUA
TRIM59	286827	27436876	ACUAAGGGCUAUUAUUGAA
TRIM59	286827	27436876	GCCAUGCUCUCAUACAUUU
TRIM59	286827	27436876	UAACGGCUCUCUGUGAUGU
TRIM59	286827	27436876	GCACUGACAAUAUCUUUAC
SIAH2	6478	55925659	AGACAUAUGUGAAUACCGU
SIAH2	6478	55925659	GCAAGCAAGCCGAGAACUU
SIAH2	6478	55925659	GCUAAUAAACCCUGCAGCA
SIAH2	6478	55925659	GACAUCGUCUUUCUAGCUA
RNF43	54894	56711321	GCAGAACAGAAAGCUAUUA
RNF43	54894	56711321	UAUGAUGUGUGGAUCCUAA
RNF43	54894	56711321	GGAGAAAGCUAUUGCACAG
RNF43	54894	56711321	GGUGGAGUCUGAAAGAUCA
IBRDC1	154214	34303935	CAUAGCGGUUGUAAUCGUU
IBRDC1	154214	34303935	GUUGUUAAGUGAUAUGCUA
IBRDC1	154214	34303935	CAUAUGACCUGCUCACAAU
IBRDC1	154214	34303935	GAACUUGGCCGUAUUGAUU
RNF186	54546	9506662	GCACCAAGACCCUGCAACA
RNF186	54546	9506662	GACAACACCUGGUCCAUCA
RNF186	54546	9506662	CAUCUACCCGGGUGUCUUA
RNF186	54546	9506662	AGCCACAUCUCUUCCAUUG
MARCH7	64844	53828927	GGUGACUUAUGUAGAAUUU
MARCH7	64844	53828927	CAUAACAGGACAUUUGAUA
MARCH7	64844	53828927	GGAGUAAUUUGACCGACAA
MARCH7	64844	53828927	GAACAUCAGACCAUACUAA
ZA20D1	56957	9910155	CCGAUUGGCCAGUGUAAUU
ZA20D1	56957	9910155	CCGAGUGGCUGAUUCCUAU
ZA20D1	56957	9910155	GCAUCUAGGUACCAAUGGA
ZA20D1	56957	9910155	UAACGGAGGGAGCAAGUAU
TRIM54	57159	34878869	GACAAUAGCCGGAGGCAGA
TRIM54	57159	34878869	GAACUUCACAGUGGGUUUC
TRIM54	57159	34878869	GGUCUUCGGUGCCCACAAG
TRIM54	57159	34878869	UCUACGGCCUGCAGCGAAA
SYVN1	84447	51317310	UCAUCAAGGUUCUGCUGUA
SYVN1	84447	51317310	GAGAAGAGAUGGUGACUGG
SYVN1	84447	51317310	CAACAUGAACACCCUGUAU
SYVN1	84447	51317310	GGAAAGGCCUCCAGCUCCU
RKHD3	84206	47894110	GAACGUGGAUCGCGCUCGA
RKHD3	84206	47894110	GCGAAGACCAAUACUUACA
RKHD3	84206	47894110	GCGCCAAUCGCAUCUGUGA
RKHD3	84206	47894110	GCGCACAACGGAAACAAUA
RNF14	9604	34577097	UUAAUGGACUUACGAAAUG
RNF14	9604	34577097	GAAUCACUGUCAAAUCUGA

RNF14	9604	34577097	CUAGCAUACUUGAAUAUUG
RNF14	9604	34577097	UUAGACGGAUGUAACAAGA
LPXN	9404	4758669	CAACGACUACCACCAACUU
LPXN	9404	4758669	UCCAGGAGCUCAAUGUCUA
LPXN	9404	4758669	GCGCAGCUCGUGUAUACUA
LPXN	9404	4758669	CUUCGGAGAUCCUUUCUAU
RNF11	26994	34452682	GAUGACUGGUUGAUGAGAU
RNF11	26994	34452682	UAGGAUAGCUCAAAGAAUA
RNF11	26994	34452682	GGGAGUGUGUGAUCUGUAU
RNF11	26994	34452682	GCACUGCUUUCAUCCUAUG
RFP2	10206	47132520	GAAGGGAGUGUGCGGAAUU
RFP2	10206	47132520	GACACUGGCACAUUCAUUA
RFP2	10206	47132520	UAAAACAGCCGAUUUCAUA
RFP2	10206	47132520	GAGACCAGCUCCAUUCAAG
TRIM58	25893	40353772	CUAUGAAGCCGGUGAAAUU
TRIM58	25893	40353772	GAAAGUCCUCGCUGCAUUG
TRIM58	25893	40353772	UCACAAGGCUGGAAGCAGA
TRIM58	25893	40353772	GAUUGGAGUUUGAGAAGCA
BFAR	51283	7706090	GGACAUCACGGUUUCUCAU
BFAR	51283	7706090	GCUACGACAUCCUGGUUAA
BFAR	51283	7706090	UAACACAGGCCGAGCGAAU
BFAR	51283	7706090	AGAAAUAUGGGAAUGAUCA
RNF19	25897	35493781	ACAAAUACCUCUUCUGACA
RNF19	25897	35493781	GCAAGUAGAUAUUGAGUCA
RNF19	25897	35493781	GGUGUAACGUUGUCUGUAA
RNF19	25897	35493781	ACGAUGUGCUGCUUAUAUA
BRPF1	7862	51173721	GAACUGGGCCCUUAAAGAA
BRPF1	7862	51173721	GAUCAAGGUUCAGCAGAUU
BRPF1	7862	51173721	GACUACAUCUGGCUGGAUA
BRPF1	7862	51173721	CGAAAGGUCUACAAGAGUU
RNF126	55658	37622891	CAUCACACAGCUCCUCAAU
RNF126	55658	37622891	GAACAAAACUGCUCCAACA
RNF126	55658	37622891	CGGAUUAUAUCUGUCCAAG
RNF126	55658	37622891	UGUCUAACCUCACCCUCUA
TRIM38	10475	24497622	GGCCCUAUUUCCAGGUUUA
TRIM38	10475	24497622	CAGCAAUGCGAAUAACUAA
TRIM38	10475	24497622	GUGUAUAACAGACUUCUUU
TRIM38	10475	24497622	CGGAUGGGAUUUAGGAGUU
RNF169	254225	51468839	GAUGAACCAUUAGUACUGA
RNF169	254225	51468839	GAACAGGACAGUGAUAAUA
RNF169	254225	51468839	UUACUAGGCUCUGAAGGUA
RNF169	254225	51468839	CCGAAGAACUAAACCAUUU
CBLL1	79872	13376203	GACAAGAUAUAGACCGUAU
CBLL1	79872	13376203	GGGGUGAGCUGUUUGCAAA
CBLL1	79872	13376203	UAUCAACCAUCGCCAUAUG
CBLL1	79872	13376203	GGGAAUGAGUCCUGGUAUA
LNX1	84708	14249127	GGAAGAAUUACUCUAACUA
LNX1	84708	14249127	GCACGGCCCUUUGAGAGAU
LNX1	84708	14249127	CGAUAGUACUCAAAGCUUU
LNX1	84708	14249127	GGAGAAUGACCGUGUGUUA
ARIH1	25820	9966762	CGAGAUAUUUCCCAAGAUU
ARIH1	25820	9966762	GAGAGUCGACGAAGGGUUU
ARIH1	25820	9966762	CCAAAUGCCAUGUCACAAU
ARIH1	25820	9966762	GGAUAUGCCUUGUCAGAUC
RNF175	285533	27734858	GGGAUGUUCUCCGUUAUUA
RNF175	285533	27734858	CACGAUUGGUCUACAAAUG
RNF175	285533	27734858	GACAAUAUCUGUGCAGUCU
RNF175	285533	27734858	CAUUUGGUGUUGUGGGUUA
NEURL	9148	21314780	CCGCAGAACUCACUCAACU
NEURL	9148	21314780	CCACAAGGCUGUCAAGAGG
NEURL	9148	21314780	CUGACUCGCUGCCCAAGUA
NEURL	9148	21314780	GGUAACAACUUCUCCAGUA

LNX2	222484	34222215	GGACAUACAUUCUGCUACA
LNX2	222484	34222215	CAACGAAACACCUUUGAUU
LNX2	222484	34222215	CUUCAUAGCUGCCACGAUA
LNX2	222484	34222215	CCAAGUGGCUCUUCAUAAA
RNF17	56163	14277695	GAACUUUCUUGUUACGAUA
RNF17	56163	14277695	UGAUGUACAUUUAGAAGCA
RNF17	56163	14277695	GCCCUGAUGUGAUAAUUGA
RNF17	56163	14277695	GCAAGAGCAUUACAAUUAU
RNF24	11237	10518498	GGGCAGAGAACAUUGUAUA
RNF24	11237	10518498	GAAUUUACAUGAGCUCUGU
RNF24	11237	10518498	ACAGAAAGUGCCUUAUUAA
RNF24	11237	10518498	GCUCGGAUUUCCCACAUUA
RNF146	81847	33636757	GGACGUCGCAGGAAGAUUA
RNF146	81847	33636757	GAUGGACAGUGCACAGUAA
RNF146	81847	33636757	CCGUAAACCUAGCAAGAGA
RNF146	81847	33636757	GGAUGUAUCUGCAGUUGUU
PHF2	5253	24797090	AGGAGUUUGUGGACUAUUA
PHF2	5253	24797090	GCUCAAGAUCGACGAGUUU
PHF2	5253	24797090	ACGGGAAACUACUCCUUUA
PHF2	5253	24797090	CUUCACAGCUAAUCAAAGA
PEX10	5192	24797087	UCUCAGAUGUGGCCUACUU
PEX10	5192	24797087	GAGAGAGCCGUUUCCAGAA
PEX10	5192	24797087	CGCAGAAGGACGAGUACUA
PEX10	5192	24797087	UCACCACACUUGCAGGCUA
MYLIP	29116	38788242	GGUGAAAGUUUAUGGCUAA
MYLIP	29116	38788242	CCAGAACACUGCCAAGUAU
MYLIP	29116	38788242	GACUUUAGCCCAAUUAAUA
MYLIP	29116	38788242	UAACAGAGACGCACGCAUU
ZNRF1	84937	30089958	ACGAUGAUGUGCUGACUAA
ZNRF1	84937	30089958	GAACAGAUCUUGUCCGGAA
ZNRF1	84937	30089958	GGAAAUGCACUUUAUAAUG
ZNRF1	84937	30089958	GCAUAGUGGUUUCAAGUGC
TRIM11	81559	24497621	CCGAAGACCUCAAGGCGAA
TRIM11	81559	24497621	CCGAUGGGUCACUGCUAUU
TRIM11	81559	24497621	UGGGUGAGUUCGAGCGUCU
TRIM11	81559	24497621	AGGCGAAGCUGGAGAAGUC
TRIM5	85363	15011943	GCAGAAAGUUGAUCAUUGU
TRIM5	85363	15011943	GGAAUCCUGGUUAAUGUAA
TRIM5	85363	15011943	GAGAACAUACGGCCUAAUC
TRIM5	85363	15011943	GGGUGUGGAUGGCGUCAUA
TRAF7	84231	45594313	GCAAGAGUGCCCGCUACAA
TRAF7	84231	45594313	GGGCACACGUUCUGUAGGA
TRAF7	84231	45594313	CGGUGAAGCUGUGCUGUCA
TRAF7	84231	45594313	GACCUUCGGACCCGCCUUU
PHF8	23133	32698699	GGUGAUGGAAGACGAAUUU
PHF8	23133	32698699	CUCAUGAGUGUGCGAGAUA
PHF8	23133	32698699	UGGGAGUGUUAGUAAUCAA
PHF8	23133	32698699	UCAAGAAGGCAGAGCGAAA
RNF170	81790	21361953	GGCCAAAUAUCAAGGUGAA
RNF170	81790	21361953	GAUCUACCCACUUUACUGA
RNF170	81790	21361953	GGGCAACCCAGAUCUAUUA
RNF170	81790	21361953	UAGACAAACGGUAACCUUA
RNF182	221687	22749454	GAGCCUCGUUAUUCUUAUG
RNF182	221687	22749454	UACAAUCGAUACAAUCUGA
RNF182	221687	22749454	GUUAGUAGCCUGCCCGAUG
RNF182	221687	22749454	UAUGGCACCUCCUUCUUAA
UHRF1	29128	16507203	GCCAUACCCUCUUCGACUA
UHRF1	29128	16507203	GUAAAGUGGAGGAGACGUU
UHRF1	29128	16507203	GCGGAACAGUCUUGUGAUC
UHRF1	29128	16507203	GCAAGGGCAUGGCCUGUGU
DKFZP547C195	257160	46409321	GAAUGUUCGUGACCAAUUU
DKFZP547C195	257160	46409321	GAACAUUGCUGUACAGACU
DKFZP547C195	257160	46409321	UGUAAGCUAUGUCUAAUGU

DKFZP547C195	257160	46409321	GGAAAGAGUUACUGGUACU
PJA2	9867	41281511	GCAGGAGGGUAUCAGACAA
PJA2	9867	41281511	GAAGCACCCUAAACCUUGA
PJA2	9867	41281511	GUUAGAUUCUGUACCAUUA
PJA2	9867	41281511	AGACUGCUCUGGCCCAUUU
PML	5371	67089153	GGACAUGCACGGUUUCCUG
PML	5371	67089153	GGAAAGAUGCAGCUGUAUC
PML	5371	67089153	GCAACCAGUCGGUGCGUGA
PML	5371	67089153	GAGCUCAAGUGCGACAUCA
UBOX5	22888	40806195	GGAGAAGUGUAACCGCAGU
UBOX5	22888	40806195	GGCGGUAUCCCUUGUAUCA
UBOX5	22888	40806195	GCAACAAGAUAUCAGCUGA
UBOX5	22888	40806195	GACAGUAACUUUGGUGUAA
TRIM65	201292	38679904	AGCCAAGCCUGUGGACUUA
TRIM65	201292	38679904	GCGCCAACCGUCACUUCUA
TRIM65	201292	38679904	GCAGCCAGAUCCAGAACUC
TRIM65	201292	38679904	GUAGGACCCUGACCCUGUG
DPF2	5977	21536317	GAAGAUACUCCCAAGCGUC
DPF2	5977	21536317	GACCAACAGUCGAGCGCGA
DPF2	5977	21536317	GGAGUAGCCCAGAGCAAUU
DPF2	5977	21536317	CCGGACAGCUGUACUCCUA
JARID1C	8242	11321604	GACAAGACUCUGCGGAAGA
JARID1C	8242	11321604	CUACGAACGCAUUGUUUAU
JARID1C	8242	11321604	GAGCAGGCUACCCGGGAAU
JARID1C	8242	11321604	UCGCAGAGAAAUCGGGCAU
BAZ1B	9031	14670391	CCAAUAAGCUGCACACUAA
BAZ1B	9031	14670391	AUAAGGAGAUAGUUCGAUA
BAZ1B	9031	14670391	GCAUUCAGAUUGGUGGAUA
BAZ1B	9031	14670391	GCACGUAGAUCGCCACGAA
AOF1	221656	60685218	GUAAUUGAGUAGCAGUAAA
AOF1	221656	60685218	CCUAAGGACUACCACAAUA
AOF1	221656	60685218	CCGGUAGGCAGGCGAAGAA
AOF1	221656	60685218	AAGGCAGGCUGUACGGCAA
RNF113A	7737	5902157	GCGAAAGAAUUGAUUGCUA
RNF113A	7737	5902157	UUACUUAGGUUUCCCAUAA
RNF113A	7737	5902157	GACAAGAUCUAUCGGGGAA
RNF113A	7737	5902157	GGGUGGUGCUUCCGACUUG
PCGF5	84333	83816965	GAGGUUGGACAAUACAUUA
PCGF5	84333	83816965	CAACAACAGUGACGGAAUG
PCGF5	84333	83816965	GAAGAAAUUCAUUCGAUGU
PCGF5	84333	83816965	ACAAAUUGCUAUCUGUCUA
MDM4	4194	4505138	CCACGAGACGGGAACAUUA
MDM4	4194	4505138	CGUCAGAGCUUCUCCGUAA
MDM4	4194	4505138	CCUAAAGAUGCGUAUAUAA
MDM4	4194	4505138	AAGCAUGGGAGAACAGUUA
SSA1	6737	56549143	UCUCAGAGCUAGAUCGAAG
SSA1	6737	56549143	GAAUGUGCCUUUACAGGAC
SSA1	6737	56549143	GCAGCACGCUUGACAAUGA
SSA1	6737	56549143	AAUAUUGGAUCACAAGGAU
KIAA1333	55632	33620748	GAGCCUAUUCCAAGUUAUA
KIAA1333	55632	33620748	CCAUCGACCUGUUCAAAUA
KIAA1333	55632	33620748	UAAACCCACUCCUUCAAUU
KIAA1333	55632	33620748	UCGAAGAUGUCGUUGCAAA
LOC399940	399940	51470744	GCAAGAAGACAAUACAGCA
LOC399940	399940	51470744	GAACACAGAAACCACCAGA
LOC399940	399940	51470744	CAUCAAAUGGGACAAAUAA
LOC399940	399940	51470744	GAAAGAACCAAUAGUCAUA
TRIM50A	135892	31342404	GUACGAAGCCUUUGCCUGC
TRIM50A	135892	31342404	GGCUCUACCUGCACUAUGA
TRIM50A	135892	31342404	CGUAAGGGCAAGCUGAACA
TRIM50A	135892	31342404	GAACUCACCUUCUUCGAUG
MEFV	4210	4557742	GCAUAUGACACCCGCGUAU
MEFV	4210	4557742	GCAGGCCCUUCGAAGUGUA

MEFV	4210	4557742	GCCCGCAAAUCCAGAAAUU
MEFV	4210	4557742	GCUACUGGGUGGUGAUAAU
RBBP6	5930	38683864	AGACAUAUGUUAUAAGUCG
RBBP6	5930	38683864	UAUUGGAGGUGUUAAAUCU
RBBP6	5930	38683864	CGAAAGAAGAAUAUACUGA
RBBP6	5930	38683864	CCUCUAAACUCAACUAUGA
UNK	85451	89041820	CCACCAAGUGCAACGACAU
UNK	85451	89041820	CCUGAAAGAAUUCCGCACA
UNK	85451	89041820	GGAGAAGACUUUCGAUAAC
UNK	85451	89041820	AAGCACAAAUACAGGUCGU
RNF111	54778	37595552	GAGUUGAGAUGAUUAAUAG
RNF111	54778	37595552	GCAAAUAGUAGUUCUGGUA
RNF111	54778	37595552	UGAGACGUCUUCCAUGUAU
RNF111	54778	37595552	GCGCUUCCAUUAACAAUUC
TRIM68	55128	37622898	GAGAGAUCCUGAAGACUUA
TRIM68	55128	37622898	GAGGAUGUCUUGAUAAUGU
TRIM68	55128	37622898	GAAGGGAAAUGAGUACCGA
TRIM68	55128	37622898	GAACUGGGGUUACACCUGU
TRIM47	91107	54792145	CCAAAGGUGUCAAGAGGGU
TRIM47	91107	54792145	GUACGGGACGGCAAGAUGA
TRIM47	91107	54792145	CCCAAGACCUCGAGAGUAC
TRIM47	91107	54792145	CAUCAAGAGUGCAGCCGUA
BIRC4	331	32528298	GAAAGAGAUUAGUACUGAA
BIRC4	331	32528298	GGACUCUACUACACAGGUA
BIRC4	331	32528298	GUAGAUAGAUGGCAAUAUG
BIRC4	331	32528298	GAACUGGGCAGGUUGUAGA
RNF208	727800	119220602	GGACAUGCCUGCCUUGGAA
RNF208	727800	119220602	AUUGUGAAUCAGUACGUGA
RNF208	727800	119220602	CAGAGAUCAUUGUCAACCA
RNF208	727800	119220602	UGAAAAGUUCCCUGAGCUA
NHLRC1	378884	40255282	GGCGAUCGCUCCAUCAAAG
NHLRC1	378884	40255282	GUGUUUAGCUCAAGUAUGC
NHLRC1	378884	40255282	CAGAAUGGGAUUGUGGUAA
NHLRC1	378884	40255282	GAUCAAGCUUGUCAUUGGA
BIRC8	112401	44680138	GCAGCGCGGUUAUUGAUUU
BIRC8	112401	44680138	CUAUAGGUCAAGAGGAUAA
BIRC8	112401	44680138	GUGAUACCAUCUUCCCUAA
BIRC8	112401	44680138	GAUGUACUCCGUUAACAAA
TRIM48	79097	34147361	UGGGAGAAGCUGUUAAAGA
TRIM48	79097	34147361	GAGCUUCGUUGAUGUUAGU
TRIM48	79097	34147361	GAUAUUACUCUGCAUCACA
TRIM48	79097	34147361	GCAUAAAGACAAUACAGCA
MARCH1	55016	53759068	CAAGAUAUCAACCAUGUAU
MARCH1	55016	53759068	CAUGUAAUGUAAACACAGA
MARCH1	55016	53759068	GGUUUGGUCUUUGUAUGUA
MARCH1	55016	53759068	GCAGCCACGUUUGUUGUAA
MLLT10	8028	57546898	UAGCAAGACUUAGUGAUAA
MLLT10	8028	57546898	CAGCAAUGAUGUAGCAGUA
MLLT10	8028	57546898	GGCAAUAGAUCAAAUUCAU
MLLT10	8028	57546898	GUAACCAACUGGCAAUUAA
ZNF364	27246	33859667	CAAACUACCGGAAUAUAUA
ZNF364	27246	33859667	GCAAGCAACAGAUUUAGCA
ZNF364	27246	33859667	ACAAGUUGAUAUGGGUUUA
ZNF364	27246	33859667	CCUGACAGAUCUCCAGCUA
MKRN1	23608	21359891	UCAAGUCUCUCAUCGAUAG
MKRN1	23608	21359891	GAGUGGGACUUGUUUCAUG
MKRN1	23608	21359891	GCAAGUGGAGGAGUGCUAA
MKRN1	23608	21359891	UGACUUGGAUCUAUAGCAA
FLJ14627	84900	14249505	GGACGUGUGGGCGGAGUUA
FLJ14627	84900	14249505	GUAGGUUACUAGUGAAUAC
FLJ14627	84900	14249505	GACGGAACCCAGUGUAUUA
FLJ14627	84900	14249505	UGAAGGGGCACAAGAAAUU
RNF207	388591	124487386	CCGAGGAGGAGGACGCUAU

RNF207	388591	124487386	CCAGGAAGUGUCCCGGAAA
RNF207	388591	124487386	GUGCAGAGCCAAUACGAAG
RNF207	388591	124487386	GGAGGGAACACCCGACUUA
MARCH3	115123	31341961	UCCAAAGUCUGUCAAUGUA
MARCH3	115123	31341961	GCGCAAACCCAGGCCGUUA
MARCH3	115123	31341961	GGAGGAUUGUGGCAGCCUA
MARCH3	115123	31341961	UGAAUGGGCAGCCGCAGUA
TRIM74	378108	38524611	GAAAUGAGGACCACCAUGA
TRIM74	378108	38524611	GGAAGAUAGAGGAAAGUCA
TRIM74	378108	38524611	CUACAGAAAUACUAGAGGA
TRIM74	378108	38524611	CAAGGAGUCCCUAAUGCUA
RNF165	494470	57165360	CCACACAGAUGGUCGUCCA
RNF165	494470	57165360	UGUUUGGCUCUGUGCGAAA
RNF165	494470	57165360	GGAUACAUUCCAUACGAAA
RNF165	494470	57165360	UGACAAACAACUACGAAUA
TRIM10	10107	16519562	CCAGAGGAUUCGGGACUUU
TRIM10	10107	16519562	UGAGGGAGCCGGUCACUAU
TRIM10	10107	16519562	UCGCACACCUGAGGAAGUU
TRIM10	10107	16519562	CAUCAGAAGCACUCUAAUA
EEA1	8411	55770887	GCAGUCAGCUGGAAAGUCA
EEA1	8411	55770887	GAACCUUGAAGCUUUAUUA
EEA1	8411	55770887	GUUCAAACACUAAUGGAUA
EEA1	8411	55770887	GAAGCAACGGUUCAGAAUA
RFP	5987	18641280	GAACCAGCUCGACCAUUUA
RFP	5987	18641280	GAGAUGGGCGUGUGCGAGA
RFP	5987	18641280	UAAGAGAGGCUCAGUUAUA
RFP	5987	18641280	CGGAGAGUCUAAAGCAGUU
C17ORF27	57674	66571326	CAACAUCGCUCGUGGACAA
C17ORF27	57674	66571326	GUCCAUUGCUCUAAGAUUU
C17ORF27	57674	66571326	CAACACAACGGAAGCUAUA
C17ORF27	57674	66571326	ACACAGAAUUGUCAACUAA
TRIM61	391712	60099473	GGAGAGAGUGGAACUAAUU
TRIM61	391712	60099473	UCAGAAAGACCUAGAGCUU
TRIM61	391712	60099473	CUGGGUAGUUUGACUGAAA
TRIM61	391712	60099473	GGAUCUACAUGAUAGUUUC
TRIM29	23650	17402906	GGAAUUUGGUGCAUUGAUG
TRIM29	23650	17402906	AGCCGUAACUUCAUUGAGA
TRIM29	23650	17402906	GAAGCCACCCGUUACCUUU
TRIM29	23650	17402906	GAAGAGAUACUCCAUGUAC

Supplementary Table 2 The List of Primers

**Gene**	**sense (5'-3')**	**antisense (5'-3')**
hCKS1	AATCTTGGCGTTCAGCA	TTGGTTTCTTGGGTAGTGG
hCCNB1	CTTTCGCCTGAGCCTATTT	CCATCTTCTGCATCCACAT
hFN1	ATTCTGTAGGCCGTTGGA	TACTGCTGGATGCTGATGA
hSKP2	TACAGGTGGCTGTTGCG	TGGAGGTAGTTGAGCTGGA
hZEB1	GCCTGAGTCCTCTGTTTCA	GCTCTTCTGCACTTGGTTG

Supplementary Table 3 The Clinicopathologic Features of Microarrays

Patient	Survival state	Survival time	Sex	Age	Tumor location	T	N	M	TNM stage
1	1	3	male	73	gastric cardia	T4a	N3b	M0	IIIC
2	0	88	male	56	gastric angle	T3	N3a	M0	IIIB
3	1	17	female	79	gastric body	T3	N0	M0	IIA
4	0	88	female	69	gastric angle	T3	N1	M0	IIB
5	1	8	male	68	lesser curvature of gastric body	T4a	N3a	M0	IIIC
6	1	10	male	71	gastric antrum	T4a	N2	M0	IIIB
7	1	5	female	68	lesser curvature	T4a	N1	M0	IIIA
8	1	11	female	68	gastric angle	T3	N1	M0	IIB
9	1	44	male	71	gastric antrum	T3	N2	M0	IIIA
10	0	88	male	50	gastric antrum	T4a	N2	M0	IIIB
11	1	16	female	52	lesser curvature	T3	N3b	M0	IIIB
12	1	8	male	78	lesser curvature of gastric body	T3	N3b	M0	IIIB
13	0	88	male	67	gastric fundus	T3	N0	M0	IIA
14	1	0	male	75	lesser curvature of gastric antrum	T4b	N3a	M0	IIIC
15	1	7	male	75	gastric antrum	T4a	N3b	M0	IIIC
16	0	87	male	78	gastric body	T3	N0	M0	IIA
17	1	10	male	51	remnant stomach	T3	N2	M0	IIIA
18	1	67	male	74	gastric antrum	T4a	N2	M0	IIIB
19	1	4	female	67	gastric antrum	T3	N3a	M0	IIIB
20	1	12	male	62	remnant stomach	T3	N0	M0	IIA
21	1	2	male	66	lesser curvature	T4a	N3a	M0	IIIC
22	0	87	male	67	lesser curvature	T3	N0	M0	IIA
23	1	26	male	73	gastric antrum	T3	N2	M0	IIIA
24	1	14	female	76	gastric antrum	T3	N1	M0	IIB
25	1	54	male	59	gastric antrum	T4a	N2	M0	IIIB
26	1	14	male	59	gastric antrum	T3	N2	M0	IIIA
27	0	86	male	58	gastric angle	T2	N0	M0	IB
28	0	86	female	31	greater curvature	T2	N0	M0	IB
29	0	86	male	64	gastric body	T2	N1	M0	IIA
30	1	22	male	52	stomach	T3	N1	M0	IIB
31	1	19	male	54	gastric antrum	T3	N3a	M0	IIIB
32	1	15	male	63	stomach	T3	N3b	M0	IIIB
33	0	85	male	67	lesser curvature	T3	N0	M0	IIA
34	1	28	female	65	lesser curvature of gastric antrum	T3	N3b	M0	IIIB
35	1	15	male	54	gastric cardia	T3	N2	M0	IIIA
36	1	22	male	72	lesser curvature of gastric body	T3	N1	M0	IIB
37	0	84	female	46	gastric antrum	T1b	N0	M0	IA
38	1	17	female	60	gastric body	T4a	N2	M0	IIIB
39	1	54	female	68	stomach	T3	N0	M0	IIA
40	1	50	male	61	gastric cardia	T3	N2	M0	IIIA
41	1	38	male	59	gastric antrum	T3	N3a	M0	IIIB
42	0	84	female	78	gastric cardia	T4a	N2	M0	IIIB
43	1	0	male	76	gastric antrum	T4a	N3b	M0	IIIC
44	1	22	male	77	gastric fundus	T2	N2	M0	IIB
45	1	13	male	68	gastric antrum	T3	N2	M0	IIIA
46	1	27	female	52	lesser curvature of gastric cardia	T4b	N3b	M0	IIIC
47	0	83	female	52	gastric fundus	T3	N1	M0	IIB
48	1	7	female	82	gastric antrum	T3	N2	M0	IIIA
49	1	26	male	59	gastric cardia	T3	N2	M0	IIIA
50	1	28	female	62	lesser curvature of gastric body	T3	N3b	M0	IIIB
51	1	37	female	83	gastric cardia of fundus	T3	N0	M0	IIA
52	1	23	female	72	gastric cardia	T3	N3a	M0	IIIB
53	1	9	male	65	gastric cardia	T4b	N2	M0	IIIB
54	1	39	male	53	gastric antrum	T3	N1	M0	IIB
55	1	44	male	75	gastric body	T3	N1	M1	IV
56	1	10	male	50	stomach	T4a	N3a	M0	IIIC

57	1	12	female	61	stomach	T3	N3a	M0	IIIB
58	1	9	female	77	lesser curvature	T3	N1	M0	IIB
59	1	6	male	80	gastric cardia	T3	N2	M0	IIIA
60	1	16	male	81	lesser curvature of gastric antrum	T4a	N2	M0	IIIB
61	1	35	male	67	gastric cardia	T4a	N1	M0	IIIA
62	1	5	male	71	gastric antrum	T3	N3b	M0	IIIB
63	0	97	male	70	gastric fundus	T3	N0	M0	IIA
64	1	14	female	53	remnant stomach	T4a	N0	M0	IIB
65	1	35	female	52	lesser curvature of gastric antrum	T3	N3a	M0	IIIB
66	1	11	male	69	lesser curvature	T3	N3a	M0	IIIB
67	1	28	male	61	gastric body	T3	N0	M0	IIA
68	1	32	male	55	lesser curvature of gastric antrum	T3	N2	M0	IIIA
69	0	97	female	65	gastric body,gastric antrum	T2	N1	M0	IIA
70	0	97	female	53	lesser curvature of gastric body	T3	N0	M0	IIA
71	1	67	male	62	lesser curvature of gastric angle	T2	N3a	M0	IIIA
72	1	27	male	58	lesser curvature of gastric cardia	T3	N2	M0	IIIA
73	1	10	male	49	lesser curvature of gastric antrum	T3	N3a	M0	IIIB
74	0	96	male	71	gastric cardia	T3	N0	M0	IIA
75	1	8	male	64	lesser curvature of gastric body	T3	N3a	M0	IIIB
76	1	35	male	79	gastric antrum	T3	N2	M0	IIIA
77	1	18	female	67	lesser curvature	T3	N3a	M0	IIIB
78	1	2	male	73	pylorus	T3	N0	M0	IIA
79	1	24	male	57	lesser curvature	T3	N2	M0	IIIA
80	1	46	male	54	gastric body	T3	N1	M0	IIB
81	1	42	male	63	lesser curvature	T3	N2	M0	IIIA
82	1	6	female	52	gastric body	T3	N3a	M0	IIIB
83	1	58	male	81	gastric antrum	T3	N2	M0	IIIA
84	1	23	female	77	lesser curvature of gastric antrum	T2	N1	M0	IIA
85	1	25	female	72	lesser curvature	T4a	N2	M0	IIIB
86	1	5	female	62	lesser curvature of gastric antrum	T4a	N3a	M0	IIIC
87	1	15	male	68	gastric antrum	T3	N3a	M1	IV
88	1	15	female	79	gastric antrum	T3	N1	M0	IIB
89	0	94	male	72	gastric antrum	T1a	N0	M0	IA
90	1	3	male	81	lesser curvature of gastric antrum	T2	N3a	M0	IIIA
91	1	22	female	72	gastric antrum	T3	N3a	M0	IIIB
92	1	22	male	55	gastric body	T3	N3a	M0	IIIB
93	1	18	male	80	gastric cardia	T3	N3a	M0	IIIB
94	1	24	male	57	stomach	T4a	N3b	M1	IV
95	1	27	female	65	gastric antrum	T3	N1	M0	IIB
96	1	31	male	59	gastric antrum	T3	N3a	M0	IIIB
97	1	11	male	83	gastric antrum	T3	N2	M0	IIIA
98	1	39	male	75	gastric antrum	T4a	N2	M0	IIIB
99	1	9	male	59	gastroenteric stoma	T4a	N3a	M0	IIIC
100	1	42	female	73	lesser curvature	T3	N3a	M0	IIIB
101	1	19	male	59	gastric antrum	T3	N3a	M0	IIIB
102	1	2	male	78	gastric antrum	T3	N3a	M0	IIIB
103	1	63	male	82	gastric fundus	T3	N2	M0	IIIA
104	1	24	male	62	gastric cardia of fundus	T3	N3a	M0	IIIB
105	1	21	male	73	gastric cardia	T3	N1	M0	IIB
106	1	44	male	58	gastric cardia of fundus	T3	N3a	M0	IIIB
107	1	43	male	67	gastric cardia of fundus	T4a	N1	M0	IIIA
108	1	27	male	67	lesser curvature of gastric antrum	T3	N2	M0	IIIA
109	1	30	male	71	gastric antrum	T3	N1	M0	IIB
110	1	13	male	80	gastric cardia	T4a	N2	M0	IIIB

Unedited blot and gel

Full unedited gels for Figure. 1

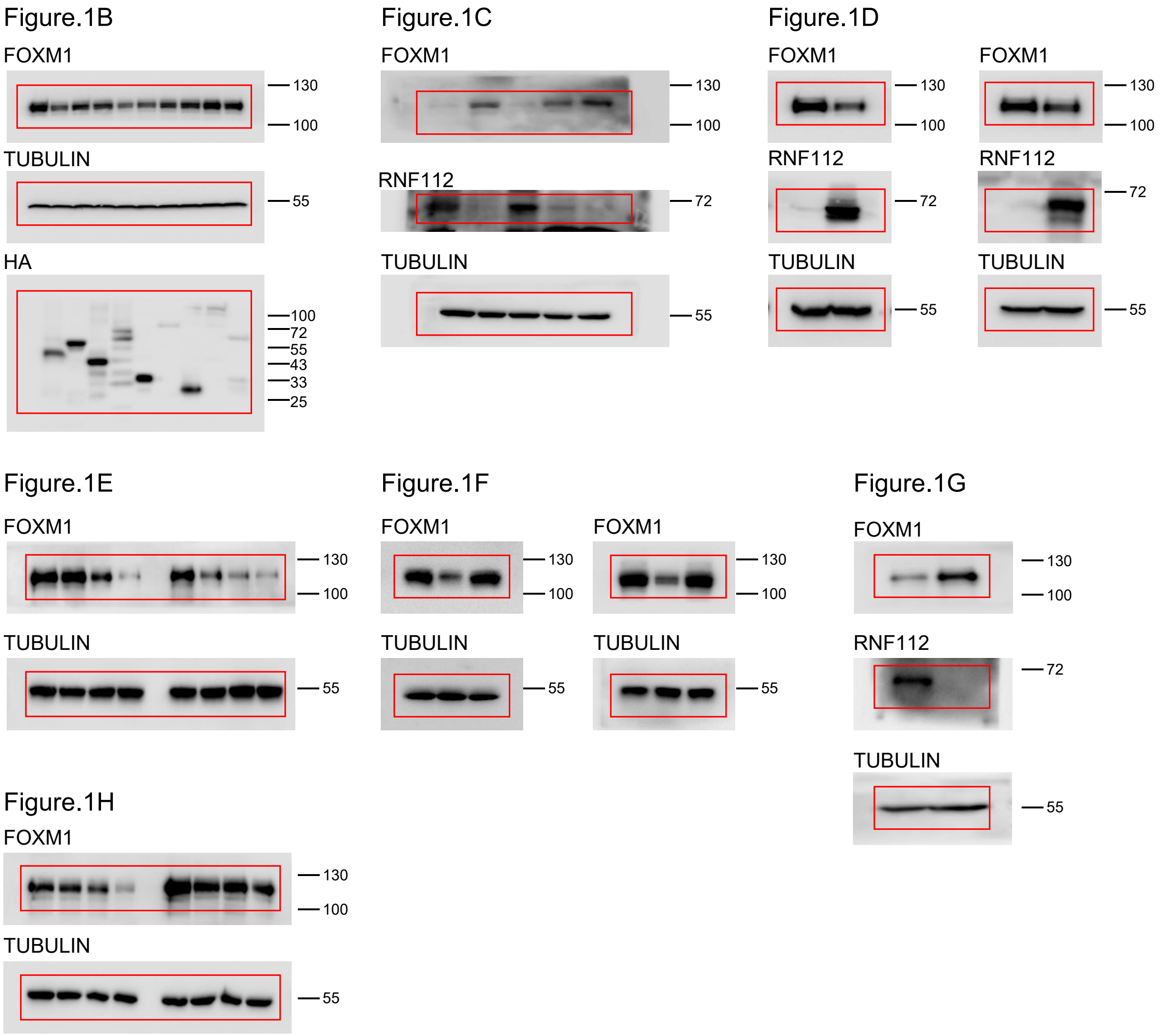

Unedited blot and gel

Full unedited gels for Figure. 2

Figure.2D

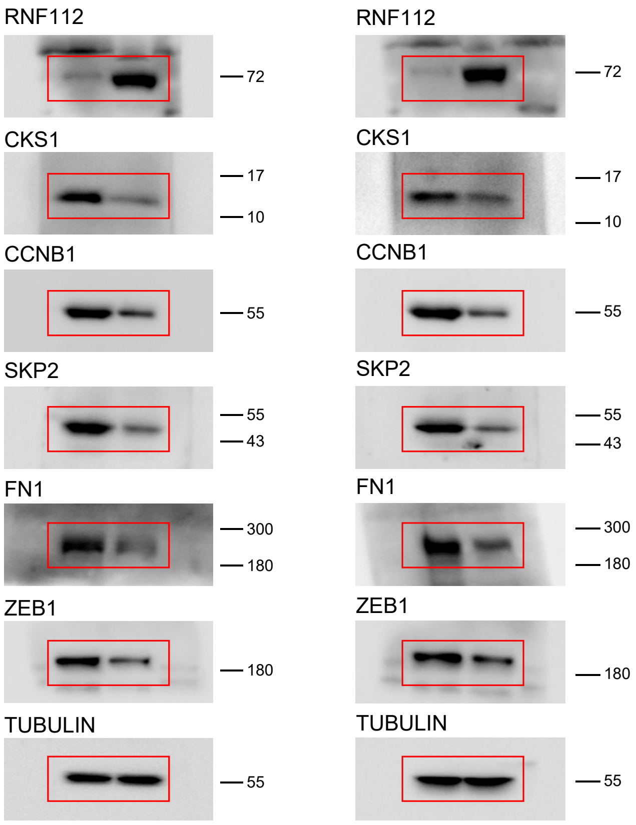

Figure.2I

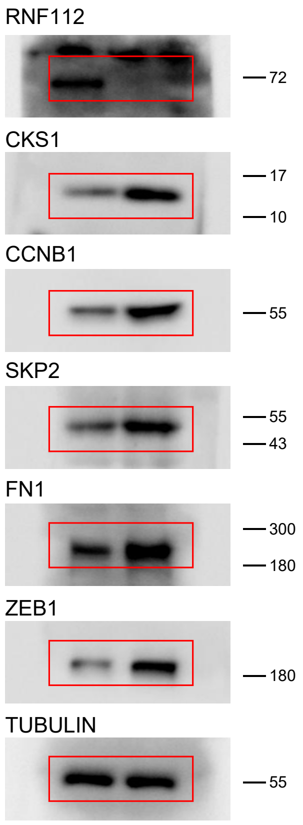

Unedited blot and gel

Full unedited gels for Figure. 3

Figure.3D

FOXM1

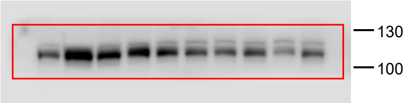

RNF112

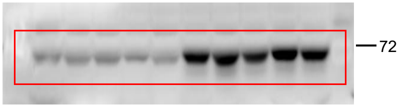

GAPDH

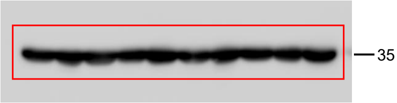

Unedited blot and gel

Full unedited gels for Figure. 4

Figure.4A

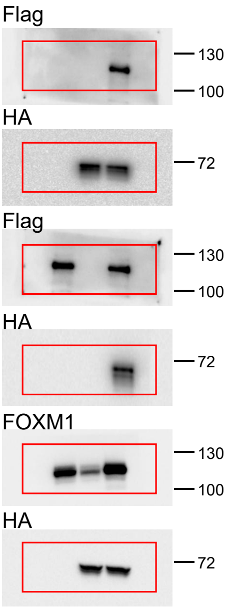

Figure.4B

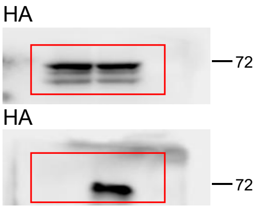

Figure.4F

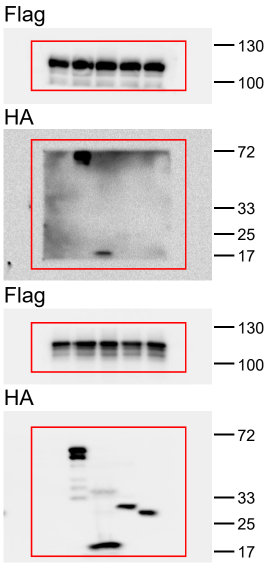

Figure.4G

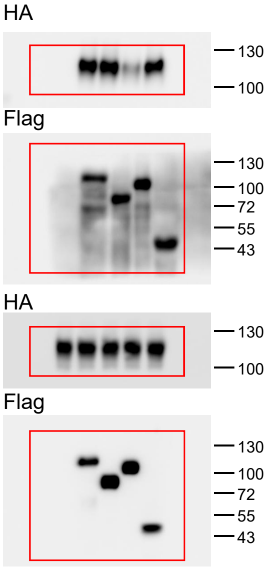

Figure.4H

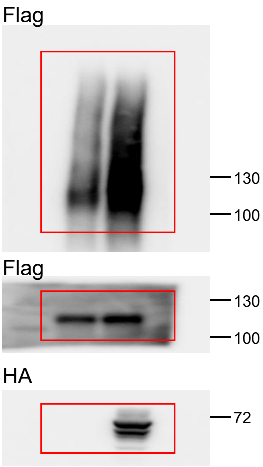

Unedited blot and gel

Full unedited gels for Figure. 5

Figure.5B

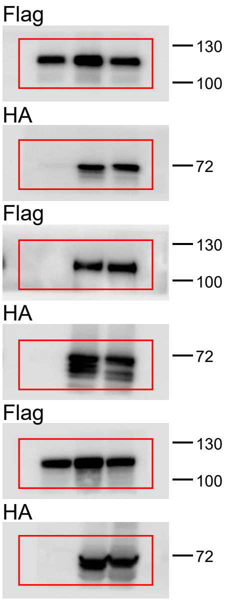

Figure.5C

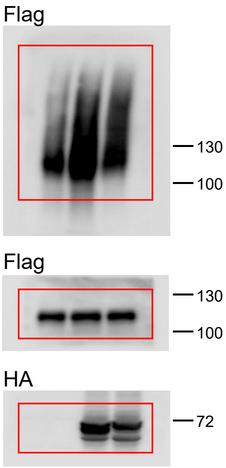

Figure.5D

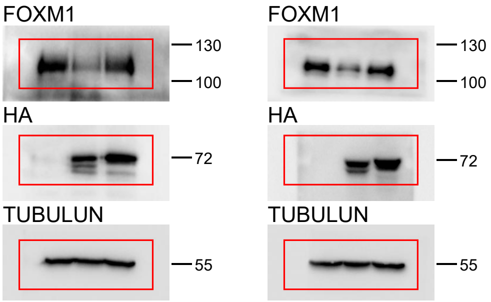

Unedited blot and gel

Full unedited gels for Figure. 6

Figure.6A

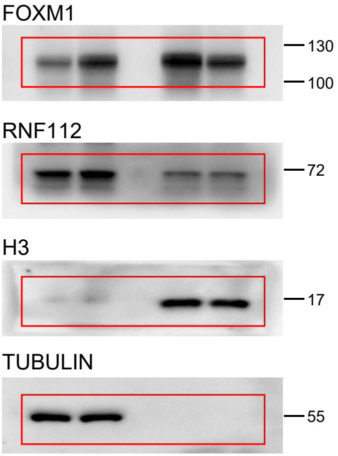

Figure.6B

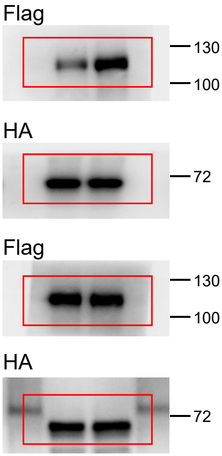

Figure.6C

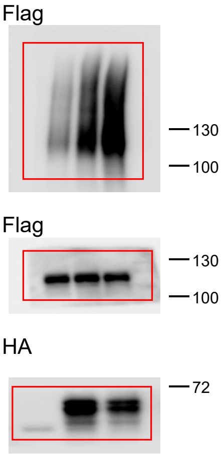

Figure.6E

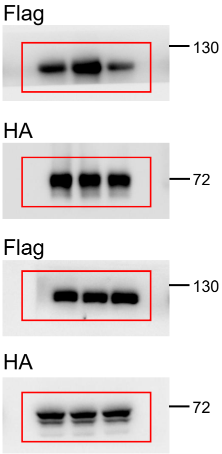

Figure.6F

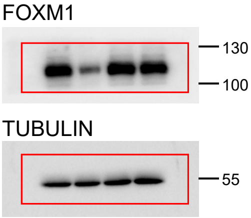

Unedited blot and gel

Full unedited gels for Figure. 7

Figure.7A

FOXM1

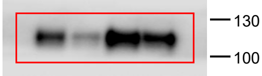

RNF112

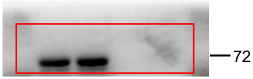

TUBULIN

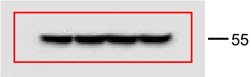

Unedited blot and gel

Full unedited gels for Supplemental Figure. 2

Supplemental Figure. 2

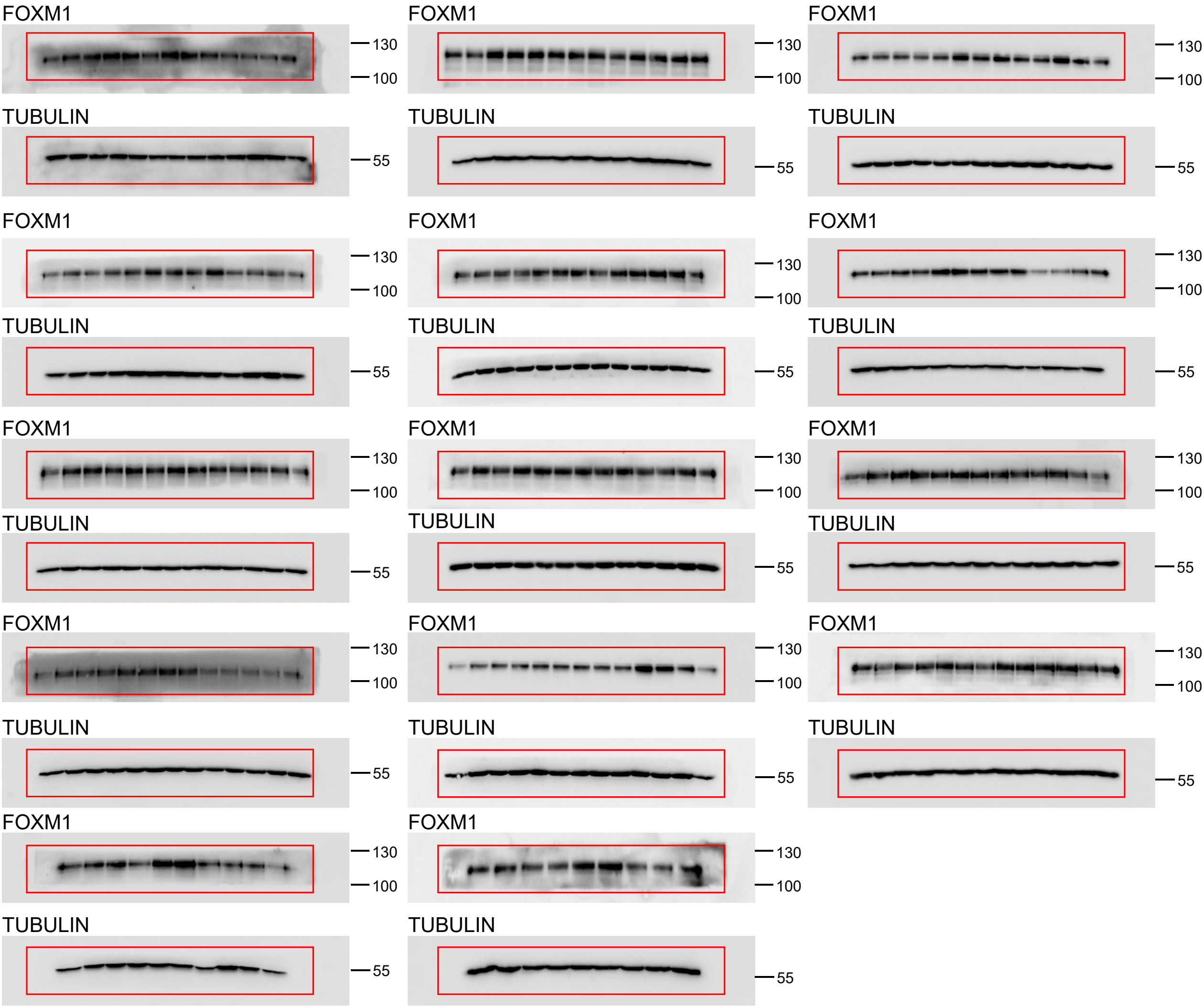

Unedited blot and gel

Full unedited gels for Supplemental Figure. 5

Supplemental Figure. 5C

FOXM1

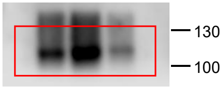

RNF112

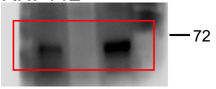

TUBULIN

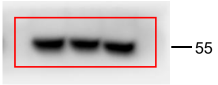

Unedited blot and gel

Full unedited gels for Supplemental Figure. 6

Supplemental Figure. 6C

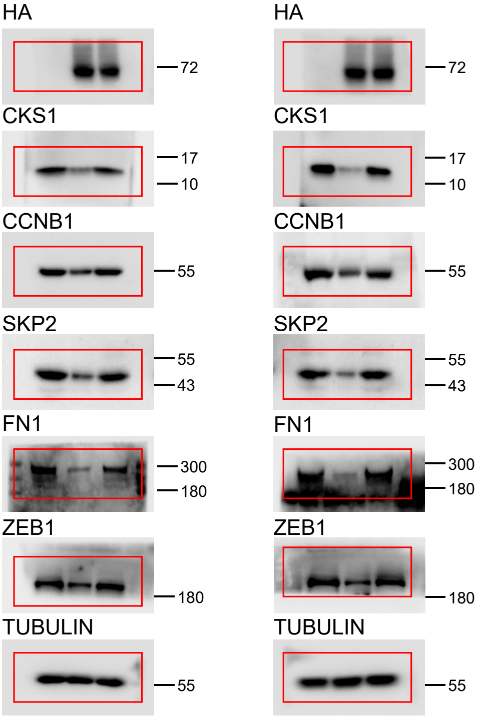

Supplemental Figure. 6F

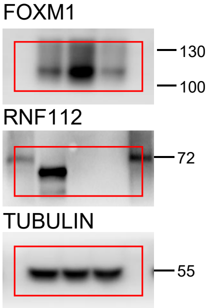

Unedited blot and gel

Full unedited gels for Supplemental Figure. 7

Supplemental Figure. 7D

FOXM1

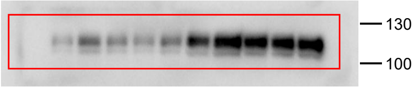

RNF112

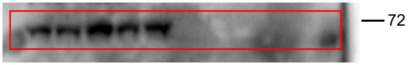

GAPDH

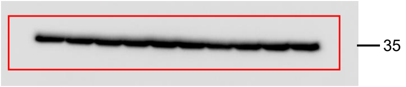

Unedited blot and gel

Full unedited gels for Supplemental Figure. 10

Supplemental Figure. 10A

FOXM1

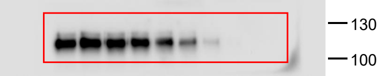

FOXM1

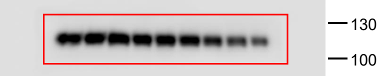

Supplemental Figure. 10B

RNF112

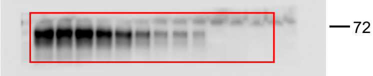

RNF112

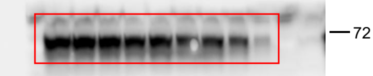

Unedited blot and gel

Full unedited gels for Supplemental Figure. 11

Supplemental Figure. 11B

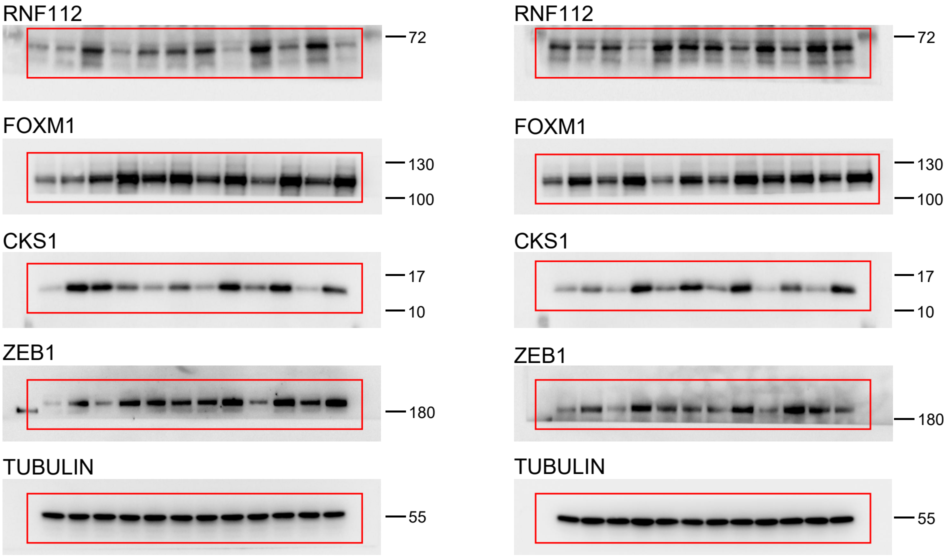

Supplement: Supplemental data [file jciinsight-8-166698-s270.pdf]
